# Anti-windup strategies for biomolecular control systems facilitated by model reduction theory for sequestration networks

**DOI:** 10.1126/sciadv.adl5439

**Published:** 2024-08-21

**Authors:** Maurice Filo, Ankit Gupta, Mustafa Khammash

**Affiliations:** Department of Biosystems Science and Engineering, ETH Zürich, 4058 Basel, Switzerland.

## Abstract

Robust perfect adaptation, a system property whereby a variable adapts to persistent perturbations at steady state, has been recently realized in living cells using genetic integral controllers. In certain scenarios, such controllers may lead to “integral windup,” an adverse condition caused by saturating control elements, which manifests as error accumulation, poor dynamic performance, or instabilities. To mitigate this effect, we here introduce several biomolecular anti-windup topologies and link them to control-theoretic anti-windup strategies. This is achieved using a novel model reduction theory that we develop for reaction networks with fast sequestration reactions. We then show how the anti-windup topologies can be realized as reaction networks and propose intein-based genetic designs for their implementation. We validate our designs through simulations on various biological systems, including models of patients with type I diabetes and advanced biomolecular proportional-integral-derivative (PID) controllers, demonstrating their efficacy in mitigating windup effects and ensuring safety.

## Teaser

Biomolecular protection circuits are designed and computationally validated to enhance control and ensure safety in biosystems.

## INTRODUCTION

Control theory has long been fundamental to the advancement of engineering systems, providing principles and methods to guide system behavior toward desired outcomes. More recently, these principles have been adopted within the growing field of synthetic biology, leading to the creation of sophisticated, biomolecular feedback controllers capable of executing complex tasks (*1*–*4*). In particular, integral feedback controllers (IFCs) play a unique role among these controllers due to their ability to enable robust perfect adaptation (RPA) (*5*) in the system. RPA is similar to homeostasis, a recurrent principle in biology, but it is even more stringent. In essence, RPA means that a specific variable in the system is always regulated to maintain a fixed steady-state value, referred to as the setpoint, regardless of any constant disturbances or uncertainties that might be present. To this end, the antithetic integral feedback (AIF) controller was introduced to provide a chemical reaction network (CRN) that mathematically realizes integral feedback control via a sequence of chemical reactions (*6*). This biomolecular controller proved capable of delivering RPA not only in deterministic settings but also amid the noise inherent in stochastic settings at the population level. Furthermore, it was also shown that the AIF controller indeed represents the minimal design which is both necessary and sufficient for achieving RPA in noisy environments (*7*, *8*). Building upon the theoretical groundwork, several genetic implementations of integral and quasi-IFCs were realized in various settings including in vivo (*7*, *9*, *10*, *11*, *12*), in vitro (*13*), and optogenetically in silico (*14*).

Following the introduction of the AIF controller, several studies were undertaken to elucidate its dynamic performance, stability, inherent trade-offs, and tuning (*15*–*20*). To circumvent the limitations of the AIF controller, a new generation of more advanced controllers was developed. These controllers augment the AIF motif with supplementary circuitry that realizes proportional-integral-derivative (PID) controllers (*21*–*25*). This advanced class of controllers has been demonstrated to enhance dynamic performance and reduce noise, embodied as cell-to-cell variability, all while preserving the RPA property. Alternative strategies have also been explored to enhance the performance the AIF controller. These include using molecular buffering (*26*) to enhance stability, integrating ultrasensitive modules (*27*, *28*) to amplify controller gain and reduce steady-state error when the effect of dilution (*7*, *20*) is substantial, and distributing controller components across multiple cellular populations (*29*–*31*) to improve modularity and reduce burden induced by shared resources.

Despite their promise, there are still challenges that limit the full potential of biomolecular IFCs. Among these challenges, one of the most noteworthy is integral windup (*32*)—a phenomenon where the integral term in the controller accumulates an error over time, causing the controller to overshoot (or undershoot) the setpoint when the actuation (or sensing) is saturating. This problem can lead to extended periods of poor performance and even system instability. It becomes especially relevant in the context of biomolecular systems where saturation is often encountered due to positivity of molecular concentrations, promoter saturation, and limited resources such as enzyme concentrations and energy molecules (*33*–*36*). Integral windup is not a challenge exclusive to biomolecular systems. Its origins and subsequent mitigation strategies can be traced back to the 1930s (*37*) in industrial applications. Actuators, with their saturating upper and lower operational limits, have long been recognized as major contributors to integral windup manifesting as poor dynamic performance or even instability. In particular, a positivity constraint on actuators is nothing but a special case of actuator saturation where the lower bound is inherently zero. This specific form of saturation, associated with the zero bound, has been acknowledged for decades as a potential catalyst for instability arising from integral windup. Classic scenarios include valves incapable of being more than fully open or fully closed (*38*–*40*) or heaters incapable of cooling (*41*). This phenomenon is universal, reinforcing the pervasive relevance and importance of the integral windup issue across diverse domains. This is especially more pronounced in biology where challenges imposed by promoter saturation, rate-limiting steps, and shared limited resources (*33*, *34*, *42*, *43*) in gene regulatory networks, receptor-ligand saturation (*44*, *45*) in signaling pathways, and enzyme saturation (*46*) in metabolic pathways are common. For instance, IFCs show considerable promise in therapeutic applications where precise regulation of biological variables is crucial. Instabilities in such settings manifest as deviations from tolerable ranges, resulting in severe consequences. This is illustrated through a simulation study of glucose regulation in patients with diabetes, conducted using a US Food and Drug Administration (FDA)–approved mathematical model (refer to Numerical simulations) (*47*, *48*). To this end, when used in any practical application, IFCs should always be accompanied by anti-windup mechanisms (*37*).

**Fig. 1.**
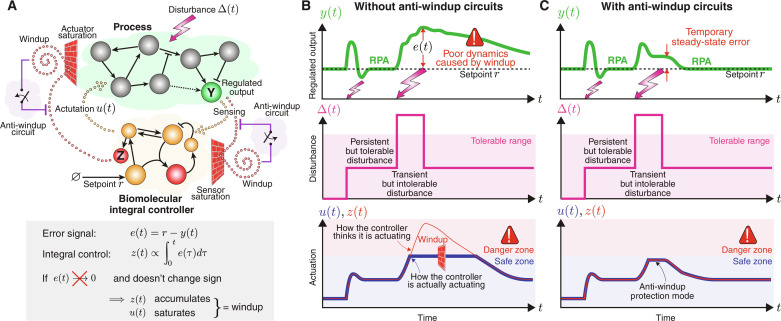
Integral windup. (**A**) A graphical portrayal of integral windup in a biomolecular system. The closed-loop network comprises an arbitrary process in a feedback configuration with a controller network involving integral feedback control. The controller’s objective is to drive the regulated output **Y** to a prescribed setpoint *r* at steady state, despite constant disturbances and uncertainties—a property referred to as RPA. The controller operates by sensing the output **Y**, computing the integral of the error *e*(*t*) ≜ *r* − *y*(*t*), and feeding the integral control action back into the process. At steady state, $\overset{\cdot }{z}=0$ and thus the error converges to zero. However, saturations in the actuator and/or sensor might obstruct this process, preventing the error from reducing to zero. This results in the integral of the error accumulating, leading to windup. This causes certain molecular species, denoted as **Z**, to increase uncontrollably, symbolized by the spiral “winding up” due to bouncing off of a saturation wall. Therefore, equipping the controller with anti-windup circuitry becomes indispensable. (**B**) A typical behavior in the absence of anti-windup circuits. Tolerable disturbances Δ can be promptly rejected by the controller, ensuring RPA. However, when the disturbance exceeds the manageable threshold, actuation *u* hits its saturation limit, and a control species **Z** starts to accumulate, triggering windup. Even when the disturbance subsides, the error takes an extended period to return to zero because *z*(*t*) is substantially beyond the safe zone, resulting in a prolonged “unwinding” process. (**C**) Anti-windup circuits offer a solution by stepping in only when necessary, preventing the controller from reaching a dangerous state. This intervention introduces a smaller steady-state error, helps avoid the windup process, and ensures that, once the severe disturbance subsides, the error can quickly return to zero.

For a better understanding of integral windup within the framework of biomolecular controllers, consider the visual illustration depicted in Fig. 1A. This schematic portrays a closed-loop network consisting of an arbitrary process to be regulated and a feedback controller network incorporating an IFC. In this setting, the output variable of interest is species **Y**, which is sensed by the controller network. Subsequently, the controller network operates on the process, aiming to robustly navigate the levels of **Y** toward a specific target setpoint despite constant disturbances and uncertainties in the process. In some cases, the designated setpoint may demand a degree of actuation that exceeds the capacity of the actuator, due to unavoidable saturation constraints. This leads to a situation where the controller persists in attempting to achieve an unattainable level of actuation and, as a result, certain controller species accumulates. In mathematical terms, consider the IFC represented by a variable denoted by $z\propto \int_{0}^{t}‍e\left(\tau \right)d\tau$ , where *e*(*t*) ≜ *r* − *y*(*t*) is the error signal denoting the current deviation of the output from the desired setpoint *r*. If the control action *u*(*t*) reaches saturation, thereby inhibiting the controller’s ability to drive the error to zero, then the integral continues to grow and thus leading to windup. Note that a similar scenario may also happen when the sensor saturates. Therefore, it becomes essential to design a biomolecular protection circuit implementing an anti-windup strategy. This circuit should be capable of intervening to mitigate such phenomena when they occur while remaining passive when the system is operating within a safe regime. In other words, the controller should be capable of operating in two modes: a normal mode where integral feedback facilitates RPA and a protection mode where aiming to achieve RPA is forfeited as a necessary compromise to uphold safety measures.

Figure 1B graphically demonstrates how windup could occur due to an intense transient disturbance beyond the system’s tolerance. This can lead to a saturation in the control action *u*, resulting in an accumulation in the control variable *z*. Consequently, it causes a persistent error *e* that lingers long after the disturbance has subsided, thus taking an extended period to return to zero. Figure 1C depicts the impact of implementing an anti-windup strategy. Essentially, this strategy prevents the accumulation of the control variable *z* beyond a defined safe threshold by “forgetting” to integrate when operating in the danger zone. As a result, it incurs a nonzero steady-state error but expedites the return to zero error once the disturbance recedes. In a sense, windup could be conceptualized as a “trauma” inflicted on the system due to a severe disturbance. The ramifications of this trauma tend to linger, taking a substantial amount of time to fade even after the cause of the trauma has subsided. In this context, an anti-windup strategy can be seen as a therapeutic intervention, helping the controller to move past its “traumatic” experience by promoting a form of forgetfulness toward the disruptive event.

Various anti-windup strategies have been proposed in the literature of traditional control systems (*37*, *49*, *50*). These strategies modify the integrator’s behavior within the controller upon detecting saturation, effectively preventing excessive error accumulation and its detrimental consequences. Nonetheless, implementing these strategies in the context of biomolecular systems poses unique challenges, primarily due to the inherent complexity and constrained structure of nonlinear CRNs. Here, we address this challenge by proposing CRN-based biomolecular realization of three anti-windup topologies. We start by phenomenologically modeling the dynamics of each topology to motivate their effectiveness in preventing windup. We then present realizations of these topologies as CRNs. We propose genetic realizations of the anti-windup circuitry—a step forward toward their implementation in living cells.

### Notation

Uppercase bold letters, e.g., **Y**, are reserved for species names. Their corresponding lowercase letters, e.g., *y*(*t*), represent their deterministic concentrations as time-varying signals, where *t* is the time. Over-bars, e.g., $\bar{y}≜\underset{t\to \infty }{\text{lim}}y\left(t\right)$ , represent steady-state values when they exist. A tilde, e.g., $\tilde{y}\left(t\right)≜y\left(t\right)-\bar{y}$ , represents the deviation from the steady-state value, and a hat, e.g., $\hat{y}\left(s\right)$ , represents the Laplace transform of $\tilde{y}\left(t\right)$ , where *s* is the Laplace variable. The *s* and *t* variables are suppressed when they are clear from context. $R_{+}$ and $R_{-}$ denote the set of nonnegative and nonpositive real numbers, respectively. **e***_i_* is a vector, of the appropriate size, whose entries are all zeros except the *i*th entry being 1. Let *f* be a function, then range(*f*) denotes its range. Let *g* be another function, then *f* ∘ *g* denotes the composition of the two functions, that is, (*f* ∘ *g*)(*x*) = *f*[*g*(*x*)]. If *z* is a scalar or a time varying signal, then *z*^+^ ≜ max (*z*, 0) and *z*^−^ ≜ max (−*z*, 0). ‖.‖ denotes the 𝐜^2^ norm.

## RESULTS

We begin by laying out the theoretical foundation, including the introduction of key definitions pertaining to the process to be controlled. We also provide concrete examples to clarify the definitions. We subsequently close the loop using the classical IFC suffering from possible actuator and sensor saturations and extend this discussion to the biomolecular AIF controller, drawing a rigorous mathematical link to the classical IFC. We then demonstrate how windup may arise with AIF control even in the absence of molecular saturations due to the positive nature of the AIF motif. We show how this observation fits the classical IFC framework. Further, we delve into the occurrence of windup due to molecular saturations and present three different topologies, complemented by suitable CRN realizations, that effectively implement anti-windup strategies. Potential genetic implementations are also proposed. Last, we demonstrate the efficacy of our anti-windup designs using three numerical simulations, including an FDA-approved model for glucose-insulin response in patients with diabetes (type I) (*47*, *48*).

### Open-loop system

**Fig. 2.**
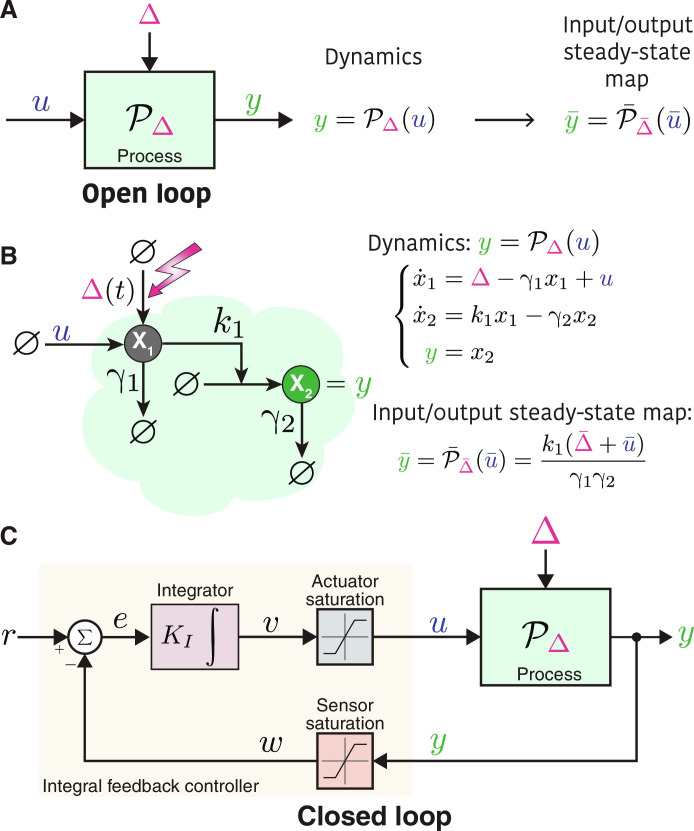
Classical integral feedback control. (**A**) Process or open-loop system. The input and output of the process are respectively denoted by *u* and *y*, while Δ denotes a disturbance or uncertainty. The dynamics of the process are described by a (nonlinear) dynamic operator P_Δ_, and its input/output steady-state map for a constant disturbance or uncertainty $\bar{\Delta }$ is denoted by $P_{\bar{\Delta }}$ . (**B**) Gene expression as an example of a simple process. The process is composed of two molecular species **X**_**1**_ and **X**_**2**_ representing the mRNA and proteins whose degradation rates are denoted by γ_1_ and γ_2_, respectively. Note that the translation rate is denoted by *k*_1_, and Δ represents a disturbance entering the dynamics as a basal transcription rate which could be due to a mutation in the promoter or constitutive expression from a duplicate gene. The input to this open-loop system is an induced transcription rate denoted by *u*, while the output is the protein concentration *y* = *x*_2_. The dynamics and input/output steady-state map are given here. (**C**) Closing the loop with the classical IFC. Ideally, the integral controller senses the output *y* and computes the integral of the error *e*(*t*) ≜ *r* − *y*, which signifies the deviation of the output from the setpoint *r*, multiplies this integral by a gain *K_I_*, and feeds the result back to the process through the actuator. In more practical scenarios, however, sensor and actuator saturation may occur, impeding the faithful transmission of signals between the controller and the process. The equations describing the dynamics of the closed-loop system are presented in Eq. 9.

Consider a general process $P_{\Delta }$ to be controlled, depicted in Fig. 2A, which is modeled as a nonlinear dynamical system with input and output signals *u* and *y*, respectively. The process is parameterized by Δ which can be thought of as an external disturbance or uncertainty. For simplicity, we consider processes that are single-input single-output throughout the paper, that is, *u*, *y* ∈ R; however, this can be easily generalized to multiple-input multiple-output systems. An example of such process $P_{\Delta }$ is given by the following nonlinear state-space model

$$P_{\Delta }:\left\{\begin{array}{l} \overset{\cdot }{x}=f_{\Delta }\left(x,\;u\right);x\left(0\right)=x_{0}\\ y=g_{\Delta }\left(x,\;u\right) \end{array}\right.$$ (1)

where ***x*** ∈ $R^{n}$ represents the internal state vector and ***x***_0_ represents the initial condition. Without loss of generality, we will proceed under the assumption that initial conditions are zero, unless specified differently. Let ${\bar{P}}_{\bar{\Delta }}$ denote the steady-state input/output map of the process. For the nonlinear state-space realization given in Eq. 1, the steady-state input/output map is given implicitly as the following set of nonlinear algebraic equations

$$\bar{y}={\bar{P}}_{\bar{\Delta }}\left(\bar{u}\right)\;⟺\;\left\{\begin{array}{c} 0=f_{\bar{\Delta }}\left(\bar{x},\;\bar{u}\right)\\ \bar{y}=g_{\bar{\Delta }}\left(\bar{x},\;\bar{u}\right) \end{array}\right.$$ (2)

The objective here is to design a feedback controller that endows the output *y* with RPA, that is, it steers *y* to a prescribed steady-state value or setpoint $\bar{y}=r$ , despite the presence of constant disturbances or uncertainties and regardless of the initial conditions.

Let us define four important concepts that are solely related to the controlled process and disturbance represented as P_Δ_. In the following definitions, we denote by U and D as the set of feasible inputs and disturbances, respectively.

#### Definition 1 (supporting input)

A supporting input, if it exists, for a given desired setpoint *r* and a disturbance $\bar{\Delta }$ is an input value that will result in a steady-state output value equal to the setpoint *r*. That is, a supporting input $\bar{u}$ satisfies ${\bar{P}}_{\bar{\Delta }}\left(\bar{u}\right)=r$ and thus $\bar{u}\in {\bar{P}}_{\bar{\Delta }}^{-1}\left(r\right)$.

#### Definition 2 (admissible setpoint)

A given setpoint *r* is admissible if it admits a feasible supporting input. That is, for a given setpoint *r* and disturbance or uncertainty $\bar{\Delta },\exists$ , a feasible supporting input $\bar{u}\in U$ such that ${\bar{P}}_{\bar{\Delta }}\left(\bar{u}\right)=r$.

#### Definition 3 (set of admissible setpoints)

For a given process $P_{\Delta }$ and a set of feasible inputs U , the set of admissible setpoints, denoted by $R\left(P_{\Delta },U\right)$ , is the set of all possible admissible setpoints. That is

$$R\left(P_{\Delta },U\right)≜\left\{r\in \mathbb{R}:\;\exists \bar{u}\in U\;\text{with}\;{\bar{P}}_{\bar{\Delta }}\left(\bar{u}\right)=r\right\}$$

#### Definition 4 (set of admissible disturbances)

For a given admissible setpoint *r*, the set of admissible disturbances, denoted by $D_{r}\left(P_{\Delta },U,D\right)$ , is the range of all feasible disturbances that do not destroy the admissibility of the desired setpoint *r*. That is

$$D_{r}\left(P_{\Delta },U,D\right)≜\left\{\bar{\Delta }\in D:\exists \bar{u}\in U\;\text{with}\;{\bar{P}}_{\bar{\Delta }}\left(\bar{u}\right)=r\right\}$$

Note that we drop the arguments of R and $D_{r}$ whenever they are clear from context. Before we provide examples, we make three remarks on the introduced definitions.

#### Remark 1

Admissibility is a concept that depends solely on the process to be controlled, the actuation mechanism (e.g., production, removal, and saturation) and disturbance or uncertainty; it is completely independent of the controller structure. Hence, if for a given actuation mechanism and disturbance, a desired setpoint *r* ∉ R is not admissible, it means that there is absolutely no controller (regardless of its structure) that can deliver such a setpoint.

#### Remark 2

Admissibility is an algebraic steady-state property since it is ultimately related to the solvability of a set of algebraic equations. Hence, admissibility is a concept that is independent of stability, and, as such, if a setpoint is admissible, the dynamics might still be unstable.

#### Remark 3

Throughout the paper, we have, for the sake of simplicity, presumed that for any given desired setpoint *r*, the corresponding supporting input $\bar{u}≜P_{\bar{\Delta }}^{-1}\left(r\right)$ , if it exists, is unique. This assumption can be readily relaxed because the exact value of the supporting input is not of primary importance, as long as it is admissible and results in a stable closed-loop fixed point with an output coordinate $\bar{y}=r$.

#### Example 1 (gene expression)

Consider the simple model for gene expression given in Fig. 2B. The actuation and disturbance here take the form of production rates *u* and Δ, respectively. Both are assumed to have no saturation. Consequently, only nonnegative inputs and disturbances are feasible, that is, $U=D={\mathbb{R}}_{+}$ . The steady-state input/output map is affine in the input $\bar{u}$ as depicted in Fig. 2B. As a result, for a given desired setpoint $\bar{y}=r$ , the supporting input is $\bar{u}=\frac{\gamma_{1}\gamma_{2}r}{k_{1}}-\bar{\Delta }$ . Requiring the supporting input to be feasible ( $\bar{u}\geq 0$ ) yields a condition on the setpoint given by $r\geq \frac{k_{1}\bar{\Delta }}{\gamma_{1}\gamma_{2}}$ . Consequently, the set of admissible setpoints is given by

$$R=\left[\frac{k_{1}\bar{\Delta }}{\gamma_{1}\gamma_{2}},+\infty \right]$$ (3)

If there is no basal transcription ( $\bar{\Delta }=0$ ), then all nonnegative setpoints are admissible. For a given setpoint *r*, the set of admissible disturbances is calculated by searching for the disturbances that preserve feasibility of the supporting input $\left(\bar{u}\geq 0\right)$ to obtain

$$D_{r}=\left[0,\frac{\gamma_{1}\gamma_{2}}{k_{1}}r\right]$$ (4)

#### Example 2 (stable unimolecular networks)

Consider a general unimolecular network of *L* species, denoted by ***X*** ≜ {**X**_**1**_, **X**_**2**_, ⋯, **X**_**L**_}, reacting among each other via *K* reaction channels. Let *S*_Δ_ and λ_Δ_(.), respectively, denote the stoichiometry matrix and propensity function of the network in the absence of an external actuation, parameterized by a disturbance or uncertainty Δ. For unimolecular reactions, the propensity function is affine in the species concentrations, that is, λ_Δ_(***x***) ≜ *W*_Δ_***x***+***b***_Δ_, where *W*_Δ_ is a *K* × *L* matrix and ***b***_Δ_ is a *K* × 1 vector with nonnegative entries. Without loss of generality, let the output of interest be **X**_**L**_ and the actuated input be **X**_**1**_ where the actuation takes the form of a production reaction, that is, $\emptyset \overset{u\left(t\right)}{\to }\;X_{1}$ and so $U={\mathbb{R}}_{+}$ . The dynamics are thus given by

$$P_{\Delta }:\left\{\begin{array}{c} \overset{\cdot }{x}=S_{\Delta }W_{\Delta }x+S_{\Delta }b_{\Delta }+e_{1}u\\ y=e_{L}^{T}x \end{array}\right.$$ (5)

Let us assume that the dynamics are Hurwitz stable, which is equivalent to assuming that the eigenvalues of *S*_Δ_*W*_Δ_ have strictly negative real parts over a range of disturbances or uncertainties Δ. Then, the steady-state input/output map $\bar{y}={\bar{P}}_{\bar{\Delta }}\left(\bar{u}\right)$ is calculated by setting the time derivative in Eq. 5. to zero to obtain

$$\begin{array}{c} \bar{y}={\bar{P}}_{\bar{\Delta }}\left(\bar{u}\right)=\alpha_{\bar{\Delta }}\bar{u}+\beta_{\bar{\Delta }},\\ \text{with}\left\{\begin{array}{c} \alpha_{\bar{\Delta }}≜-e_{L}^{T}{\left(S_{\bar{\Delta }}W_{\bar{\Delta }}\right)}^{-1}e_{1}\\ \beta_{\bar{\Delta }}≜-e_{L}^{T}{\left(S_{\bar{\Delta }}W_{\bar{\Delta }}\right)}^{-1}S_{\bar{\Delta }}b_{\bar{\Delta }} \end{array}\right. \end{array}$$ (6)

where $\alpha_{\bar{\Delta }}$ is the “steady-state gain” of the network in the absence of basal expression ( $b_{\bar{\Delta }}=0$ ), and $\beta_{\bar{\Delta }}$ is the “basal offset” of the network that reflects the propagation of the basal expression rates $b_{\bar{\Delta }}$ . Note that $\alpha_{\bar{\Delta }},\beta_{\bar{\Delta }}\geq 0$ . This arises from the fact that $S_{\bar{\Delta }}W_{\bar{\Delta }}$ is a Metzler and Hurwitz matrix which means that all entries of its inverse are nonpositive (*51*). The steady-state input/output map is an affine function. As a result, for a given desired setpoint $\bar{y}=r$ , the supporting input is $\bar{u}=\left(r-\beta_{\bar{\Delta }}\right)/\alpha_{\bar{\Delta }}$ . For the setpoint to be admissible, the supporting input needs to be feasible, that is, $\bar{u}\in U={\mathbb{R}}^{+}$ ; hence, the setpoint is required to satisfy $r\geq \beta_{\bar{\Delta }}$ . Consequently, the set of admissible setpoints is given by

$$R=\left[\beta_{\bar{\Delta }},+\infty \right]$$ (7)

This means that for the setpoint to be admissible, it needs to be higher than the basal offset dictated by the non-actuated process. For a given setpoint *r*, the set of admissible disturbances is

$$D_{r}=\left\{\bar{\Delta }\in D:0\leq \beta_{\bar{\Delta }}\leq r\right\}$$ (8)

Example 1 is a special case where $\alpha_{\bar{\Delta }}=k_{1}/\gamma_{1}\gamma_{2}$ and $\beta_{\bar{\Delta }}=k_{1}\bar{\Delta }/\gamma_{1}\gamma_{2}$.

### Closing the loop with the classical integral controller

According to the internal model principle (*52*), RPA can be achieved using IFCs. To this end, consider the closed-loop system depicted in Fig. 2C, where the controlled process is now in a feedback interconnection with an integral controller. Ideally, the actuator should faithfully transmit the controller’s output to the controlled process (i.e., *u* = *v*), and the sensor should faithfully transmit the output to the summation junction (i.e., *w* = *y*) to compute the deviation (or error) *e* of the sensed output *w* from the desired setpoint *r*. However, practically, the actuator and sensor may saturate beyond certain thresholds. Even worse, they may deform the actuation and sensed signals. Sensor and actuator saturations are modeled by the saturation blocks depicted in Fig. 2C. Integral feedback control is achieved by the integrator module which computes the time integral of the error to yield the feedback signal *v*(*t*) to the actuator. The full dynamics of the closed-loop system are given by

$$\begin{array}{cc} \begin{array}{c} \text{Controlled}\;\text{process} \end{array} & y=P_{\Delta }\left(u\right)\;\;\;\;\;\;e.g.,\left\{ \begin{array}{cc} \overset{\cdot }{x} & =f_{\Delta }\left(x,u\right)\\ y & =g_{\Delta }\left(x,u\right) \end{array}\right.\\ \text{Error} & e=r-w\\ \begin{array}{c} \mathrm{Integrator}\\ \mathrm{Saturations} \end{array} & \begin{array}{c} \overset{\cdot }{v}=K_{I}e\\ \left\{\begin{array}{c} u=\text{sat}\left(v;u_{\text{min}},u_{\text{max}}\right)\\ \begin{array}{cc} w & =\text{sat}\left(y;y_{\text{min}},y_{\text{max}}\right) \end{array} \end{array}\right. \end{array} \end{array}$$ (9)

where [*u*_min_, *u*_max_] and [*y*_min_, *y*_max_] represent the actuator and sensor ranges, respectively, and sat(.) is the saturation function defined, for any *z*, *a*, *b* ∈ R with *a* < *b*, as

$$\text{sat}\left(z;a,b\right)≜\left\{\begin{array}{ccc} z & \text{if} & a\leq z\leq b\\ a & \text{if} & z<a\\ b & \text{if} & z>b \end{array}\right.$$ (10)

#### Ideal setting

In the ideal setting, we have the following assumptions.

#### Assumption 1 (full admissibility)

All setpoints are admissible by the controlled process for all disturbances or uncertainties, i.e., $R=U=D_{r}=\mathbb{R},\forall r\in \mathbb{R}$.

#### Assumption 2 (no saturations)

The sensor and actuator do not exhibit any saturations, i.e., *u*_min_ = *y*_min_ = − ∞ and *u*_max_ = *y*_max_ = ∞.

Under Assumptions 1 and 2, we have

$$\forall r,\bar{\Delta }\in \mathbb{R},\exists \bar{u}={\bar{P}}_{\bar{\Delta }}^{-1}\left(r\right),\;u=v,\text{and}\;w=y$$ (11)

and, as a result, the dynamics given in Eq. 9 boils down to

$$\left\{\begin{array}{c} y=P_{\Delta }\left(u\right)\\ \overset{\cdot }{u}=K_{I}\left(r-y\right) \end{array}\right.⟹\left(\bar{u},\bar{y}\right)=\left[{\bar{P}}_{\bar{\Delta }}^{-1}\left(r\right),r\right]$$ (12)

This guarantees that the output *y* will converge at steady state to the setpoint *r*, independent of $\bar{\Delta }$ , as long as the closed-loop system remains stable, that is, RPA is achieved.

#### Non-ideal setting

The fixed point of the dynamics of the non-ideal setting can be computed by setting the time derivatives in Eq. 9 to zero to obtain the following set of algebraic equations

$$\left\{\begin{array}{l} \bar{y}={\bar{P}}_{\bar{\Delta }}\left(\bar{u}\right)\\ \bar{u}=\text{sat}\left(\bar{v};u_{\text{min}},u_{\text{max}}\right)\\ \bar{w}=\text{sat}\left(\bar{y};y_{\text{min}},y_{\text{max}}\right)\\ \bar{w}=r \end{array}\right.$$ (13)

The following lemma characterizes the existence of the fixed point.

#### Lemma 1

Consider the closed-loop system depicted in Fig. 2C, where the actuator and sensor ranges are given as [*u*_min_, *u*_max_] and [*y*_min_, *y*_max_], respectively. For a given desired setpoint *r* and a steady-state disturbance or uncertainty $\bar{\Delta }$ , the closed-loop fixed point exists with a feasible supporting input $\bar{u}\in U$ if and only if

• The setpoint is admissible, i.e.,

r∈R

• The sensor does not saturate at steady state, i.e., *r* ∈ [*y*_min_, *y*_max_].

• The actuator does not saturate at steady state, i.e., ${\bar{P}}_{\bar{\Delta }}^{-1}\left(r\right)\in \left[u_{\text{min}},u_{\text{max}}\right]$.

Furthermore, the fixed point is given by

$$\left(\bar{y},\bar{u},\bar{v},\bar{w}\right)=\left[r,{\bar{P}}_{\bar{\Delta }}^{-1}\left(r\right),{\bar{P}}_{\bar{\Delta }}^{-1}\left(r\right),r\right]$$

The proof can be found in section S3A.

Lemma 1 essentially says that if the desired setpoint is inadmissible by the process, or if either the sensor or actuator saturates, then the integral controller fails and the closed-loop dynamics exhibit no equilibrium. The only way that integral control fails in achieving RPA is by losing stability of the closed loop whether this instability is due to nonexistence of a fixed point or the existence of an unstable or unreachable fixed point.

**Fig. 3.**
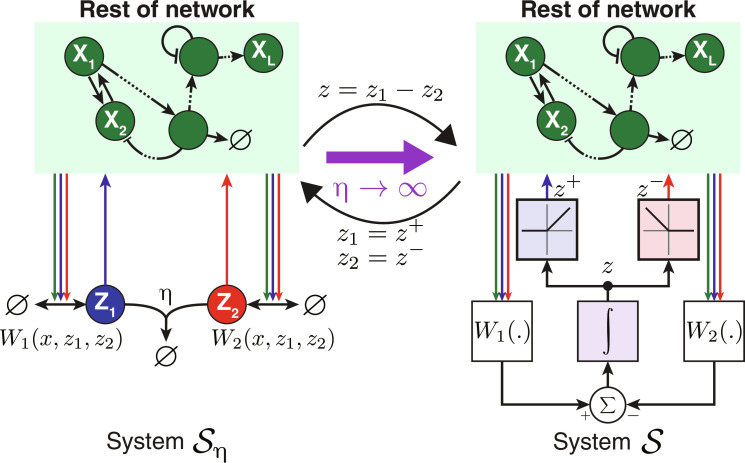
Model reduction for CRNs with fast sequestration reactions. The left schematic illustrates the full system S_η_, where η denotes a sequestration rate. In contrast, the right schematic portrays the reduced system S which accurately reflects the dynamics of S_η_ as η → ∞. The system S_η_ consists of two species, **Z**_**1**_ and **Z**_**2**_. Their production and removal rates are compactly represented by the functions *W*_1_ and *W*_2_, respectively. The rest of the network (green box) is arbitrary and remains unchanged in the reduced model. Essentially, when the sequestration rate is fast, the two species operate as a subtraction mechanism, given by *W*_1_ − *W*_2_ which is subsequently integrated in time to produce *Z* = ∫ (*W*_1_ − *W*_2_). The positive and negative portions of *Z* are isolated and relayed to the rest of the network in place of **Z**_**1**_ and **Z**_**2**_, respectively. Refer to Box 1 for the complete mathematical description.

To use Lemma 1 in a biological setting, we need to extend it into the realm of CRNs. To this end, we present in Box 1 and Fig. 3 two limit theorems that investigate the dynamics and fixed-point behaviors of complex CRNs involving fast sequestration reactions. The theorems presented offer a useful model reduction tool that is valid in both deterministic and stochastic settings. These results will be instrumental throughout this study in bridging CRN motifs with established control-theoretic architectures.

Box 1. **A model reduction result for CRNs with a fast sequestration reaction**

considers the two dynamical systems, S_η_ and S, depicted pictorially in the Fig. 3. The deterministic dynamics of each of the two systems are described by the following ordinary differential equations

$$\begin{array}{c} \begin{array}{ll} S_{\eta } & :\left\{\begin{array}{cccc} {\overset{\cdot }{x}}^{\left(\eta \right)} & =F\left[x^{\left(\eta \right)},z_{1}^{\left(\eta \right)},z_{2}^{\left(\eta \right)}\right]; & x^{\left(\eta \right)}\left(0\right) & =x_{0}\\ {\overset{\cdot }{z}}_{1}^{\left(\eta \right)} & =W_{1}\left[x^{\left(\eta \right)},z_{1}^{\left(\eta \right)},z_{2}^{\left(\eta \right)}\right]-\eta z_{1}^{\left(\eta \right)}z_{2}^{\left(\eta \right)}; & z_{1}^{\left(\eta \right)}\left(0\right) & =a\\ {\overset{\cdot }{z}}_{2}^{\left(\eta \right)} & =W_{2}\left[x^{\left(\eta \right)},z_{1}^{\left(\eta \right)},z_{2}^{\left(\eta \right)}\right]-\eta z_{1}^{\left(\eta \right)}z_{2}^{\left(\eta \right)}; & z_{2}^{\left(\eta \right)}\left(0\right) & =b, \end{array}\right.\begin{array}{c} S:\begin{array}{c} \left\{\begin{array}{ccc} \overset{\cdot }{x} & =F\left(x,z^{+},z^{-}\right); & \begin{array}{cc} x\left(0\right) & =x_{0} \end{array}\\ \overset{\cdot }{z} & =W_{1}\left(x,z^{+},z^{-}\right)-W_{2}\left(x,z^{+},z^{-}\right); & \begin{array}{cc} z\left(0\right) & =a-b, \end{array} \end{array}\right.\\ \text{with}\;z^{+}≜\text{max}\left(z,0\right)\;\text{and}\;z^{-}≜\text{max}\left(-z,0\right). \end{array} \end{array} \end{array} \end{array}$$

We make the following assumption.

**Assumption 3.** The three functions $W_{1},W_{2}:{\mathbb{R}}_{+}^{L+2}\to \mathbb{R}$ , and $F:{\mathbb{R}}_{+}^{L+2}\to {\mathbb{R}}^{L}$ are assumed to be globally Lipschitz on their domains, and their form is such that the solution of S_η_ lies in ${\mathbb{R}}_{+}^{L+2}$.

The following theorem states the convergence of the dynamics, as η → ∞, of the full system S_η_ to those of the reduced system S over any compact time interval.

**Theorem 1.** Under Assumption 3 and as η → ∞, S_η_ converges to S in the following senses: ∀T > 0 we have

$$\begin{array}{c} \underset{\eta \to \infty }{\text{lim}}\underset{t\in \left[0,T\right]}{\text{sup}}||\left[x^{\left(\eta \right)}\left(t\right),\;z_{1}^{\left(\eta \right)}\left(t\right)-z_{2}^{\left(\eta \right)}\left(t\right)\right],-,\left[x\left(t\right),\;z\left(t\right)\right]||=0\;\text{and}\;\underset{\eta \to \infty }{\text{lim}}\int_{0}^{T}‍||\left[x^{\left(\eta \right)}\left(t\right),\;z_{1}^{\left(\eta \right)}\left(t\right),\;z_{2}^{\left(\eta \right)}\left(t\right)\right],-,\left[x\left(t\right),\;z^{+}\left(t\right),\;z^{-}\left(t\right)\right]||dt=0. \end{array}$$

A numerical demonstration of Theorem 1 is provided in fig. S7.

**Remark 4.** In the limit as η → ∞, the concentration of at least one of the two species **Z**_**1**_ or **Z**_**2**_ is zero at any time. Hence, the dynamics may enter a low-copy regime where stochastic fluctuations become more relevant. To this end, we prove in section S2 that Theorem 1 is also valid in the stochastic setting. We also provide numerical validations in fig. S8.

The following theorem, on the other hand, states the convergence of the fixed points.

**Theorem 2.** Under Assumption 3 and as η → ∞, the limit point $\left(\bar{x},{\bar{z}}_{1},{\bar{z}}_{2}\right)$ of any convergent subsequence of nonnegative fixed points of system S_η_ can be transformed to a fixed point of system S given by $\left(\bar{x},{\bar{z}}_{1}-{\bar{z}}_{2}\right)$.

The following corollary characterizes the relationship between the existence of the fixed points of systems S_η_, as η → ∞ and S.

**Corollary 1.** Under Assumption 3, if system S admits a unique fixed point $\left(\bar{x},\bar{z}\right)$ , then all convergent subsequences of nonnegative fixed points of system S_η_ have a single limit point as η → ∞ given by $\left(\bar{x},{\bar{z}}^{+},{\bar{z}}^{-}\right)$ . However, if system S admits no fixed points, then there is no convergent subsequence of nonnegative fixed points of system S_η_*.*

The proofs of Theorems 1 and 2 and Corollary 1 can be found in section S1.

### AIF control

Owing to the importance of RPA in biology, the AIF controller was introduced in (*6*) to realize integral control as a CRN which endows the system with the desirable RPA properties. In this section, we establish a connection between the AIF controller and the classical IFC of Fig. 2C.

**Fig. 4.**
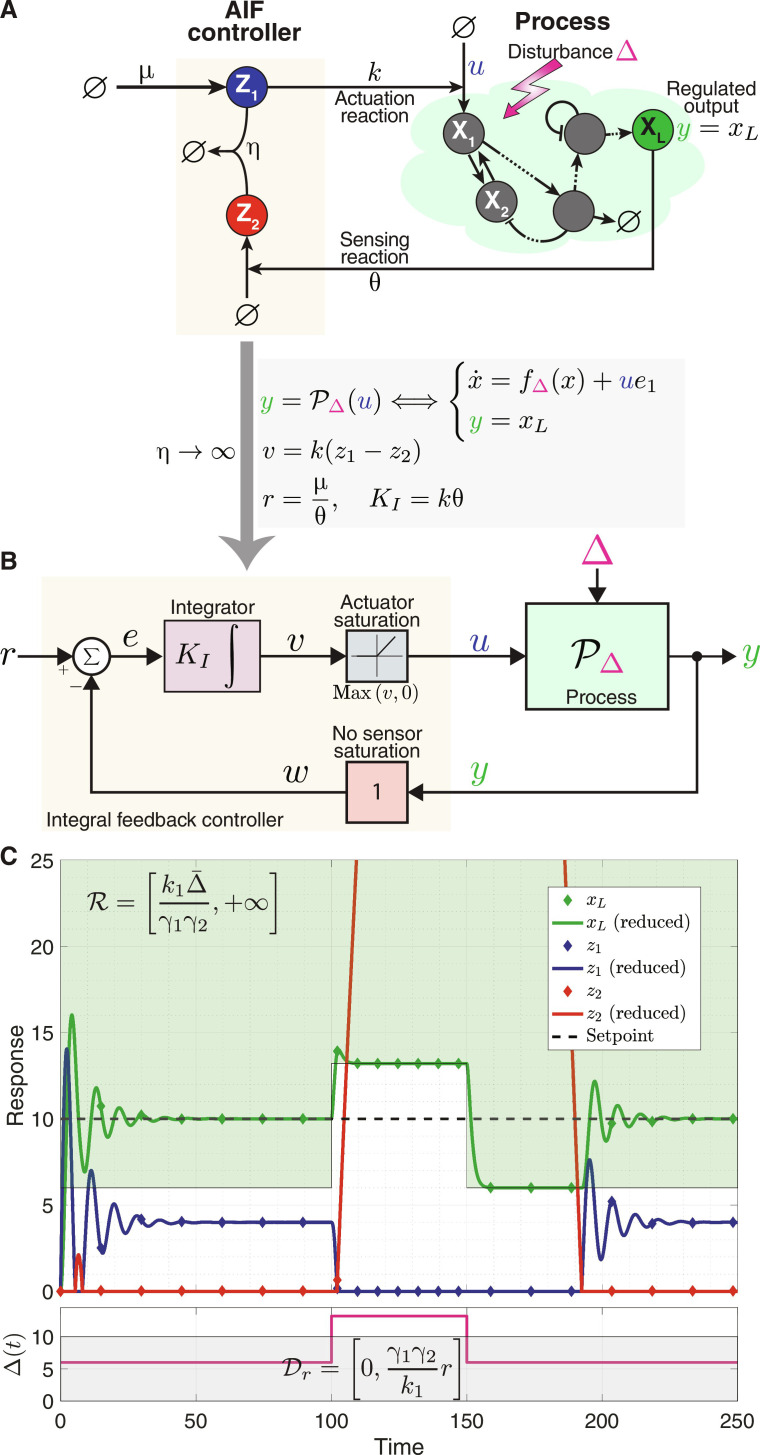
AIF control. (**A**) An arbitrary process regulated by the AIF controller. The process comprises *L* species **X**_**1**_ through **X**_**L**_, with **X**_**L**_ being the regulated output. The AIF controller comprises two species, **Z**_**1**_ and **Z**_**2**_, which sequester each other at a rate η. Sensing is realized via the catalytic production of species **Z**_**2**_ by the output **X**_**L**_ at a rate θ*x_L_*, whereas actuation is realized via the production of the input species **X**_**1**_ at a rate *u* = **k*z*_1_. The setpoint is encoded in the constitutive production of **Z**_**1**_ at a rate μ. (**B**) Block diagram describing the exact closed-loop dynamics in the limit as η → ∞. By defining the setpoint *r* and integral gain **K*_*I*_*, and an intermediate variable *v*, this diagram is generated. The diagram demonstrates that, in the strong sequestration limit, the AIF controller is exactly the classical IFC depicted in Fig. 2C, excluding sensor saturation but including a one-sided actuator saturation due to the inherent positivity of the AIF motif. (**C**) Integral windup induced by the positivity of the AIF controller. In this simulation example, the process is considered to be the gene expression model presented in Fig. 2B. The green and gray shaded areas represent the set of admissible setpoints C and disturbances D*_r_*, respectively (see Example 1 for more details). As the simulation demonstrates, the AIF controller maintains RPA as long as disturbances remain within admissible limits. However, if the disturbance shift to an inadmissible level, then the AIF controller falls short, exhibiting poor dynamic performance even after the disturbance returns to an admissible level—a clear demonstration of integral windup. Numerical values: μ = 10, η = 100, *k* = θ = γ_1_ = γ_2_ = *k*_1_ = 1 and Δ is varied.

The basic AIF controller is depicted in the closed-loop network of Fig. 4A, where the objective is to endow the regulated output **X**_**L**_ with RPA. The closed-loop network is constituted of the AIF controller, composed of two species **Z**_**1**_ and **Z**_**2**_, connected in a feedback configuration with an arbitrary network or process, composed of *L* species **X**_**1**_, **X**_**2**_,⋯, **X**_**L**_. For generality, we adopt the biomolecular control paradigm introduced in (*22*), where we assume that the controller can only interact with the process species **X**_**1**_ and **X**_**L**_ for actuation and sensing, respectively. The controller network involves four reaction channels listed in Table 1.

**Table 1.** Reactions describing the basic AIF controller.

Setpoint Reaction	$\emptyset \overset{\mu }{\to }$ Z_1_
Sensing Reaction	**X**_**L**_ $\overset{\theta }{\to }$ **X**_**L**_ + **Z**_**2**_
Comparison Reaction	**Z**_**1**_ + **Z**_**2**_ $\overset{\eta }{\to }\emptyset$
Actuation Reaction	**Z**_**1**_ $\overset{k}{\to }$ **Z**_**1**_ + **X**_**1**_

The closed-loop dynamics are thus given by

$$\begin{array}{c} \overset{\text{Process}}{y=P_{\Delta }\left(u\right)}⟺\left\{\begin{array}{l} \overset{\cdot }{x}=f_{\Delta }\left(x\right)+ue_{1}\\ y=x_{L} \end{array}\right.\\ \overset{\text{AIF}\;\text{controller}}{u=C_{\eta }\left(y,\mu \right)}⟺\left\{\begin{array}{l} {\overset{\cdot }{z}}_{1}=\mu -\eta z_{1}z_{2}\\ {\overset{\cdot }{z}}_{2}=\theta y-\eta z_{1}z_{2}\\ u=kz_{1} \end{array}\right. \end{array}$$ (14)

Note that since the actuation by the controller is carried out via a production reaction only which is assumed to have no saturation limit, the set of feasible inputs is $U={\mathbb{R}}_{+}$ . The integral action can be seen by looking at the dynamics of *z* ≜ *z*_1_ − *z*_2_ given by

$$\overset{\cdot }{z}=\mu -\theta y\Rightarrow z\left(t\right)=\theta \int_{0}^{t}‍\left[r-y\left(\tau \right)\right]d\tau$$ (15)

where *r* ≜ μ/θ denotes the setpoint. As a result, as long as closed-loop stability is maintained, the regulated output converges to the setpoint $\bar{y}={\bar{x}}_{L}=r$ regardless of the initial conditions and process uncertainties/disturbances Δ. As such, the AIF controller endows the regulated output species **X**_**L**_ with RPA. In practice, **Z**_**1**_ and **Z**_**2**_ undergo dilution, which results in a leaky integral controller and imperfect adaptation. However, studies have shown that when the dilution rate is small relative to other network rates, its impact is negligible and the integral controller’s performance remains effective (*7*, *20*).

To reveal the connection between the AIF controller and the classical IFC depicted in Fig. 2C, we examine the case where the sequestration rate is large enough. In the limit as η → ∞, applying Theorem 1 [with *W*_1_(***x***, *z*_1_, *z*_2_) ≜ μ and *W*_2_(***x***, *z*_1_, *z*_2_) ≜ θ*x_L_*] yields a reduced model for the controller given by

$$u=C_{\infty }\left(y,\mu \right)\;⟺\;\left\{\begin{array}{l} \overset{\cdot }{z}=\mu -\theta y\\ u=k\text{max}\left(z,0\right) \end{array}\right.$$ (16)

Introducing the intermediate variables *v* ≜ *kz*, *w* ≜ *y*, and *e* ≜ *r* − *w* and the integral gain *K_I_* ≜ *k*θ allows us to draw the block diagram of the closed-loop system depicted in Fig. 4B. This block diagram allows us to directly compare the AIF controller with the classical IFC depicted in Fig. 2C. The AIF controller is essentially the same as the classical IFC where the sensor has no saturation limits, that is, [*y*_min_, *y*_max_] = [−∞, +∞], while the actuator saturates only from below at zero, that is, [*u*_min_, *u*_max_] = [0, +∞]. This is intuitive since the actuation is carried out via the production of **X**_**1**_, and production cannot be negative. As a result, if the desired setpoint *r* requires a supporting input $\bar{u}<0$ , then the closed-loop fixed point with the reduced controller C_∞_ will cease to exist. However, for the full model with large η, the closed-loop fixed point may not cease to exist but instead suffer from a negative component *z*_1_ < 0 (see Corollary 1) rendering it unreachable (recall that the positive orthant is invariant under the dynamics of CRNs), and as a result, the dynamics become unstable. One can think of the AIF controller R_η_ with finite η as an integral controller coupled with “dynamic saturation” from below at zero, as compared to the case with infinite η where the saturation max(*z*,0) becomes static.

Note that if one has the luxury of changing the actuation mechanism or even adding more actuation mechanisms, then the issue of nonexistent or unreachable fixed point can be circumvented. For instance, by actuating via both production and degradation of **X**_**1**_, the control action in Eq. 14 becomes *u* = *kz*_1_ − *F*(*z*_2_, *x_L_*)*x*_1_, and the set of feasible inputs and actuator range become U=ℝ . Notably, for *F*(*z*_2_, *x_L_*) = γ*z*_2_ or *F*(*z*_2_, *x_L_*) = γ*x_L_*, we obtain (filtered) proportional-integral controllers [see (*22*)].

We close this section, by providing a numerical demonstration depicted in Fig. 4C where the gene expression model presented in Fig. 2B is controlled by the AIF controller. The plot shows that RPA is achieved as long as the disturbance $\Delta \in D_{r}$ is admissible. However, when Δ transitions to an inadmissible level, although transiently, integral windup manifests. In this scenario, *z*_1_ attempts to go negative, but of course cannot, due to the positivity of the system. Consequently, *z*_2_ accumulates and remains high for an extended period, even after the disturbance reverts to an admissible level. This results in poor dynamic performance induced by integral windup.

### AIF control with actuation/sensing saturation

**Fig. 5.**
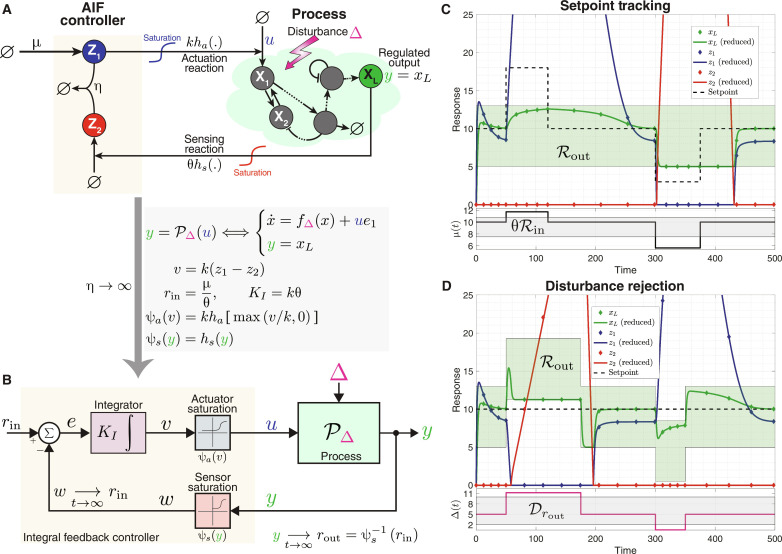
AIF control with actuator and sensor saturation. (**A**) Closed-loop network. The network structure remains the same as in Fig. 4A, except the actuation and sensing rates are now represented as *u* = *kh_a_*(*z*_1_) and θ*h_s_*(*y*), respectively, to model nonlinear, monotonically increasing, and saturating functions. The input setpoint is designated as *r*_in_ ≜ μ/θ, while the output setpoint, *r*_out_, is determined by the sensing function *h_s_* as per Eq. 19. (**B**) Block diagram describing the exact closed-loop dynamics in the limit as η → ∞. This diagram is similar to that in Fig. 4B, with the actuator and sensor saturation modules replaced by the functions ψ*_a_* and ψ*_s_*, respectively. (**C** and **D**) Integral windup induced by actuator/sensor saturation. The gene expression model from Fig. 2B is used here as the process. (C) tests setpoint tracking capabilities when the output setpoint, adjusted via μ, becomes transiently inadmissible. In contrast, (D) evaluates disturbance rejection properties by maintaining a fixed setpoint while varying the disturbance Δ until it becomes transiently inadmissible. The green shaded areas denote the set of admissible output setpoints C_out_. The gray shaded areas represent the set of admissible input setpoints (scaled by θ) and disturbances in (C) and (D), respectively (refer to Example 3 for further details). The simulations reveal that the AIF controller maintains RPA effectively as long as the setpoint and disturbance stay within admissible limits. However, when either transitions to inadmissible levels, windup emerges manifesting as substantial accumulation of either *z*_1_ or *z*_2_. Numerical values: *k*_1_ = γ_1_ = γ_2_ = 1, η = 100, θ = 15, κ*_a_* = κ*_s_* = 5, *k* = 8. In (C), Δ = 5 is fixed while μ is varied; whereas in (D), μ = 10 is fixed while Δ is varied.

Consider now the AIF controller where catalytic production reactions may saturate as depicted in the closed-loop network of Fig. 5A. The controller network here involves the same four reaction channels described in Table 1, but the propensity functions of the actuation and sensing reactions are now replaced with *kh_a_*(*z*_1_) and θ*h_s_*(*x_L_*), respectively, where *h_a_* and *h_s_* are nonlinear functions that may introduce saturation. Examples of such functions are Michaelis-Menten or Hill-type functions. The closed-loop dynamics are thus given by

$$\begin{array}{c} \overset{\text{Process}}{y=P_{\Delta }\left(u\right)}⟺\left\{\begin{array}{l} \overset{\cdot }{x}=f_{\Delta }\left(x\right)+ue_{1}\\ y=x_{L} \end{array}\right.\\ \overset{\text{AIF}\;\text{controller}}{u=C_{\eta }\left(y,\mu \right)}⟺\left\{\begin{array}{l} {\overset{\cdot }{z}}_{1}=\mu -\eta z_{1}z_{2}\\ {\overset{\cdot }{z}}_{2}=\theta h_{s}\left(y\right)-\eta z_{1}z_{2}\\ u=kh_{a}\left(z_{1}\right) \end{array}\right. \end{array}$$ (17)

The integral action can still be seen by looking at the dynamics of *z* ≜ *z*_1_ − *z*_2_ given by

$$\overset{\cdot }{z}=\mu -\theta h_{s}\left(y\right)⟹z\left(t\right)=\theta \int_{0}^{t}‍\left\{r_{\text{in}}-h_{s}\left[y\left(\tau \right)\right]\right\}d\tau$$ (18)

where, *r*_in_ ≜ μ/θ. Observe that, assuming closed-loop stability, $h_{s}\left(\bar{y}\right)=r_{\text{in}}$ . As a result, the sensing function *h_s_* determines the relationship between the input and output setpoints denoted by *r*_in_ and *r*_out_, respectively. Note that in the ideal setting of Fig. 4, *h_s_* is the identity function, and thus, the input and output setpoints match, that is, *r*_in_ = *r*_out_ = *r*. However, in general, the input and output setpoints satisfy the following algebraic equation

$$h_{s}\left(r_{\text{out}}\right)=r_{\text{in}}$$ (19)

or $r_{\text{out}}=h_{s}^{-1}\left(r_{\text{in}}\right)$ when *h_s_* is invertible. To this end, we refine the concept of admissible setpoints into two distinct categories: admissible input setpoints, denoted as C_in_, and admissible output setpoints, denoted as C_out_. These can be mapped to each other using Eq. 19 for monotonically increasing functions *h_s_*, i.e., C_in_ ≜ *h_s_*(C_out_).To reveal the connection with the classical IFC depicted in Fig. 2C, we examine, once again the case where the sequestration rate is large enough. In the limit as η → ∞, applying Theorem 1 [with *W*_1_(***x***, *z*_1_, *z*_2_) ≜ μ and *W*_2_(***x***, *z*_1_, *z*_2_) ≜ θ*h_s_*(*x_L_*)] yields a reduced model for the controller given by

$$u=C_{\infty }\left(y,\mu \right)\;⟺\;\left\{\begin{array}{l} \overset{\cdot }{z}=\mu -\theta h_{s}\left(y\right)\\ u=kh_{a}\left[\text{max}\left(z,0\right)\right] \end{array}\right.$$ (20)

Introducing the intermediate variables *v* ≜ *kz*, *w* ≜ *h_s_*(*y*), and *e* ≜ *r* − *w* and the integral gain *K_I_* ≜ kθ allows us to draw the block diagram of the closed-loop system depicted in Fig. 5B. A direct comparison with the classical IFC, depicted in Fig. 2C, reveals that the difference here is embodied in the actuator and sensor modules that are now replaced with the nonlinear functions ψ*_a_* and ψ*_s_* define in Fig. 5A. These functions not only act as saturation components but also deform the signals. The following lemma slightly generalizes Lemma 1 to the case where the saturation blocks of Fig. 2C are replaced with the monotonically increasing functions ψ*_a_* and ψ*_s_* shown in Fig. 5B.

#### Lemma 2

Consider the closed-loop system depicted in the block diagram of Fig. 5B, where ψ*_a_* and ψ*_s_* are strictly monotonically increasing functions that may saturate. For a given desired output setpoint *r*_out_ and a steady-state disturbance or uncertainty $\bar{\Delta }$ , the closed-loop fixed point exists with a feasible supporting input $\bar{u}\in U$ if and only if

• The output setpoint is admissible, i.e.,

$$r_{\text{out}}\in R_{\text{out}}$$

• The sensor does not saturate at steady state, i.e., *r*_in_ ∈ range(ψ*_s_*).

• The actuator does not saturate at steady state, i.e., ${\bar{P}}_{\bar{\Delta }}^{-1}\left(r_{\text{out}}\right)\in \text{range}\left(\psi_{a}\right)$.

Furthermore, the fixed point is given by

$$\left\{\begin{array}{ll} \bar{w}=r_{\text{in}}, & \bar{y}=\psi_{s}^{-1}\left(r_{\text{in}}\right),\\ \bar{u}={\bar{P}}_{\bar{\Delta }}^{-1}\circ \psi_{s}^{-1}\left(r_{\text{in}}\right), & \bar{v}=\psi_{a}^{-1}\circ {\bar{P}}_{\bar{\Delta }}^{-1}\circ \psi_{s}^{-1}\left(r_{\text{in}}\right) \end{array}\right.$$

The proof can be found in section S3B.

Therefore, as per Corollary 1, for the full model with large η, the nonnegative closed-loop fixed point will also not exist if any of the conditions of Lemma 2 are violated. However, the closed-loop fixed point may suffer from a negative component rendering it unreachable, and as a result, the dynamics become unstable.

Two numerical simulations are depicted in Fig. 5 (C and D) where the gene expression model presented in Fig. 2B is controlled by the AIF controller that involves actuator and sensor saturation with

$$h_{a}\left(z_{1}\right)=\frac{z_{1}/\kappa_{a}}{1+z_{1}/\kappa_{a}}\;\text{and}\;h_{s}\left(y\right)=\frac{y/\kappa_{s}}{1+y/\kappa_{s}}$$ (21)

Figure 5C demonstrates setpoint tracking, provided the desired input setpoint, tuned by μ, is admissible (i.e., *r*_in_ ∈ C_in_). Similarly, Fig. 5D demonstrates disturbance rejection when the disturbance is admissible (i.e., Δ ∈ D*_r_*). The sets of admissible input/output setpoints and disturbances, C_in_/C_out_ and D_*r*_out__, respectively, are calculated in Example 3, by modifying Example 1 to factor in the effects of saturation.

#### Example 3 (gene expression with saturation)

Consider the simple model for gene expression given in Example 1; however, replace the actuation with $u=k\frac{z_{1}/\kappa_{a}}{1+z_{1}/\kappa_{a}}$ . As a result, actuation saturation constrains the feasible inputs to the range $U=\left[0,k\right]$ . The steady-state input/output map remains unchanged as given in Fig. 2B, resulting in an identical supporting input: $\bar{u}=\frac{\gamma_{1}\gamma_{2}}{k_{1}}r_{\text{out}}-\bar{\Delta }$ . To ensure the supporting input’s feasibility ( $0\leq \bar{u}\leq k$ ), the set of admissible output setpoints is thus given by

$$R_{\text{out}}=\left[\frac{k_{1}\bar{\Delta }}{\gamma_{1}\gamma_{2}},\frac{k_{1}\left(k+\Delta \right)}{\gamma_{1}\gamma_{2}}\right]$$ (22)

Subsequently, the set of admissible input setpoints *r*_in_ ∈ C_in_ can be mapped from C_out_ using Eq. 19, i.e., C_in_ = *h_s_*(C_out_). Recalling that *r*_in_ ≜ μ/θ, we can outline the gray shaded area in Fig. 5C which is given by

$$\mu \in \theta R_{\text{in}}=\left\{\theta h_{s}\left(\frac{k_{1}\bar{\Delta }}{\gamma_{1}\gamma_{2}}\right),\theta h_{s}\left[\frac{k_{1}\left(k+\Delta \right)}{\gamma_{1}\gamma_{2}}\right]\right\}$$ (23)

Last, for a given output setpoint *r*_out_, the set of admissible disturbances can be computed by determining the disturbances that preserve the feasibility of the supporting input $\left(\bar{u}\in U\right)$ , resulting in

$$D_{r_{\text{out}}}=\left[\frac{\gamma_{1}\gamma_{2}}{k_{1}}r_{\text{out}}-k,\frac{\gamma_{1}\gamma_{2}}{k_{1}}r\right]$$ (24)

Intuitively, when the (input/output) setpoint exceeds the admissible range, species **Z**_**1**_ accumulates in a futile attempt to increase actuation *u*, which is already saturated. Conversely, when the output setpoint falls below the admissible range, species **Z**_**1**_ essentially tries to become negative to reduce actuation *u* and, as a result, lingers at zero and thus inflicting a buildup of **Z**_**2**_. These observations apply to both Fig. 5 (C and D).

### Anti-windup strategies

In this section, we introduce various anti-windup strategies designed to mitigate the unwanted effects of windup. We begin by describing these designs from a phenomenological perspective. We then present their CRN realizations. We propose genetic implementations of these anti-windup schemes.

#### Anti-windup topologies

**Fig. 6.**
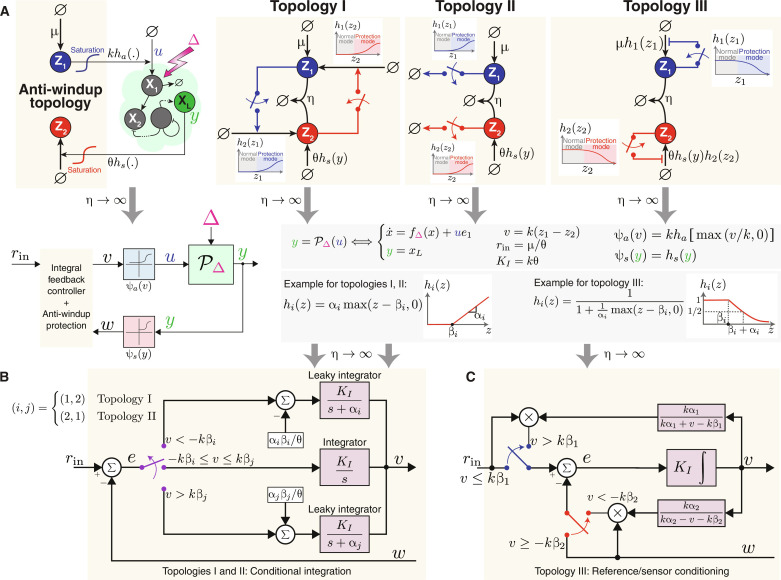
Anti-windup strategies. (**A**) Three anti-windup topologies. The leftmost diagram depicts a closed-loop network regulated by one of three anti-windup topologies. In all topologies, the controller operates normally as an AIF controller ensuring RPA, as long as **Z**_**1**_ and **Z**_**2**_ levels stay below a threshold. Otherwise, the anti-windup mechanisms (red and blue switches) activate, shifting the controller to protection mode. Topology I increases production of **Z**_**1**_ in response to high **Z**_**2**_ and vice versa. Topology II degrades **Z**_**1**_ and **Z**_**2**_ when their levels are high. Topology III reduces production rates of **Z**_**1**_ and **Z**_**2**_ when their concentrations exceed a threshold. The switches are represented by monotonic functions *h*_1_ and *h*_2_. For topologies I and II, these functions are zero until the threshold and then increase. For topology III, they are one until the threshold and then decrease. Mathematical examples of these functions, realizable as CRNs, are provided in the lower gray box (see Fig. 7). Introducing the intermediate variable *v*, input setpoint *r*_in_, integral gain *K_I_*, and functions ψ*_a_* and ψ*_s_*, as shown in the top gray box, generates the block diagram in the bottom left, depicting the reduced dynamics similar to Fig. 5B with added anti-windup dynamics. (**B**) Compact block diagram for topologies I and II. These topologies use a conditional integration approach, where the controller integrates when *v* is within the safe range [−*k*β*_i_*, *k*β*_j_*] and transitions to a leaky integrator outside this range to counter windup. Topology I is associated with (*i*, *j*) = (1,2), while topology II with (2,1). (**C**) Block diagram for topology III, implementing reference/sensor conditioning. This reduces the input reference or sensor signal when *v* is outside the safe range [−*k*β_2_, *k*β_1_]. Specifically, high positive *v* scales down the input setpoint *r*_in_, while high negative *v* scales down the sensed signal *w*.

The objective here is to present three distinct strategies to alleviate the effects of windup by augmenting the basic AIF motif with extra anti-windup circuitry. These strategies are illustrated as three topologies in Fig. 6A. Although the three topologies are mechanistically different, they share the same concept: The anti-windup circuitry is only activated to halt further growth when either of the controller species, **Z**_**1**_ or **Z**_**2**_, increases excessively. Note that although the three topologies perform comparably in computational terms, the particular choice of topologies is application specific and depends on practical considerations of the available genetic parts.

The anti-windup circuitry for all three topologies are symmetric across **Z**_**1**_ and **Z**_**2**_ (blue and red switches in Fig. 6A); thus, we will only explain the anti-windup operation on **Z**_**1**_ for brevity. In the case of topology I, anti-windup is accomplished by having **Z**_**2**_ produce **Z**_**1**_ whenever its concentration reaches a high level. As a result, the produced **Z**_**1**_ species sequester the overabundant **Z**_**2**_ species and thus effectively prevent it from growing any further. Mathematically, this is represented by the monotonically increasing function *h*_1_(*z*_2_), denoting an additional production rate of **Z**_**1**_, which exhibits a switch-like behavior (depicted by the red switch in Fig. 6A, topology I). In situations where **Z**_**2**_ concentration remains low, the switch is off, allowing the circuit to function purely as an integral controller. Conversely, when **Z**_**2**_ concentration exceeds a specified threshold, the switch is activated, transitioning the circuit into protection mode. While this mode relinquishes the pure integral control function, it safeguards the system from the detrimental effects of excessive growth. An example of such a function, *h*_1_, is given in the gray box of Fig. 6A. Here, the production rate remains zero for *z*_2_ ≤ β_1_. However, once the threshold β_1_ is exceeded, the rate becomes linear in *z*_2_ with a positive slope α_1_.

In the case of topology II, anti-windup is accomplished by having **Z**_**1**_ degrade when its concentration reaches a high level. This strategy is represented by the monotonically increasing function *h*_1_(*z*_1_), denoting a degradation rate of **Z**_**1**_, which exhibits a switch-like behavior (depicted by the blue switch in Fig. 6A, topology II).

In topology III, the anti-windup mechanism is achieved by having **Z**_**1**_ repressing its own production once its concentration surpasses a threshold. Mathematically, this scenario is depicted by the monotonically decreasing function *h*_1_(*z*_1_), which attenuates the maximum production rate μ whenever *z*_1_ exceeds the threshold. An example of such a function, *h*_1_, is given in the gray box of Fig. 6A. Here, the production rate persists at μ for *z*_1_ < β_1_. However, upon crossing the threshold β_1_, the rate decays with **Z**_**1**_. Note that this topology encompasses the anti-windup circuit introduced in (*53*).

To connect these anti-windup strategies with classical control-theoretic methodologies, we once again look at the asymptotic limit of a large sequestration rate η. By invoking the same intermediate variables as before (repeated in the gray box of Fig. 6A for convenience), we can construct the block diagrams of the controllers illustrated in Fig. 6 (B and C). The derivations once again rely on Theorem 1, and the details can be found in section S4. Both topologies I and II embody the same control-theoretic principle similar to “conditional integration”; whereas topology III applies a concept similar to “reference/sensor conditioning” (*37*, *50*, *54*).

In the case of conditional integration shown in Fig. 6B, the controller operates as a pure integral controller, with *r*_in_ being the input setpoint, as long as *v* ≜ *k*(*z*_1_ − *z*_2_) ∈ [−*k*β*_i_*, *k*β*_j_*]. However, as soon as *v* departs from this range, the controller behaves like a “leaky integrator,” represented by a transfer function of the form of $\frac{K_{I}}{s+\alpha }$ . This essentially incorporates a forgetting factor that “forgets” the need to integrate the entire history of the error signal. While leaky integrators introduce a steady-state error, they reciprocate with a stabilizing effect (*16*). The steady-state error can be shown to be proportional to the deviation of *v* from the integrator regime [−*k*β*_i_*, *k*β*_j_*]. To see this, we resort to the dynamics of *v* described in the block diagram of Fig. 6B that can be explicitly written as

$$\left\{\begin{array}{ccc} \overset{\cdot }{v}=K_{I}\left(e-\frac{\alpha_{i}\beta_{i}}{\theta }\right)-\alpha_{i}v & \text{for} & v<-k\beta_{i}\\ \overset{\cdot }{v}=K_{I}e & \text{for} & -k\beta_{i}\leq v\leq k\beta_{j}\\ \overset{\cdot }{v}=K_{I}\left(e+\frac{\alpha_{j}\beta_{j}}{\theta }\right)-\alpha_{j}v & \text{for} & v>k\beta_{j} \end{array}\right.$$ (25)

Recalling that *K_I_* ≜ *k*θ, the error at steady state, when it exists, can be written as

$$\bar{e}\;=\;\left\{\begin{array}{ccc} \frac{\alpha_{i}}{K_{I}}\left(\bar{v}+k\beta_{i}\right) & \text{ for} & \bar{\;v}<-k\beta_{i}\\ 0 & \text{ for} & -k\beta_{i}\leq \bar{v}\leq k\beta_{j}\\ \frac{\alpha_{j}}{K_{I}}\left(\bar{v}-k\beta_{j}\right) & \text{ for} & \bar{v}>k\beta_{j} \end{array}\right.$$ (26)

and hence the steady-state error is indeed proportional to the deviation of *v* from the integrator regime.

In the case of reference/sensor conditioning depicted in Fig. 6C, the controller operates as a pure integral controller, with *r*_in_ being the input setpoint, as long as *v* ≜ *k*(*z*_1_ − *z*_2_) ∈ [−*k*β_2_, *k*β_1_]. However, as soon as *v* ventures outside this range, either the reference or sensor signal undergoes a dynamic modification or “conditioning” based on *v* and thus steering a conditioned version of the error toward zero. This strategy essentially gives up trying to track the desired setpoint when necessary to prevent windup. More specifically, if *v* > *k*β_1_, then the input reference *r*_in_ is conditioned by reducing it by a factor of *k*α_1_/(*k*α_1_ + *v* − *k*β_1_). Hence, this conditioning becomes more pronounced the further *v* exceeds the integrator operating regime [−*k*β_2_, *k*β_1_]. Similarly, if *v* < *k*β_2_, then the sensor signal *w* is conditioned by reducing it by a factor of *k*α_2_/(*k*α_2_ − *v* − *k*β_2_). As a result, the conditioning is more substantial the further *v* descends from the integrator operating regime.

#### Sequestration-based switches

**Fig. 7.**
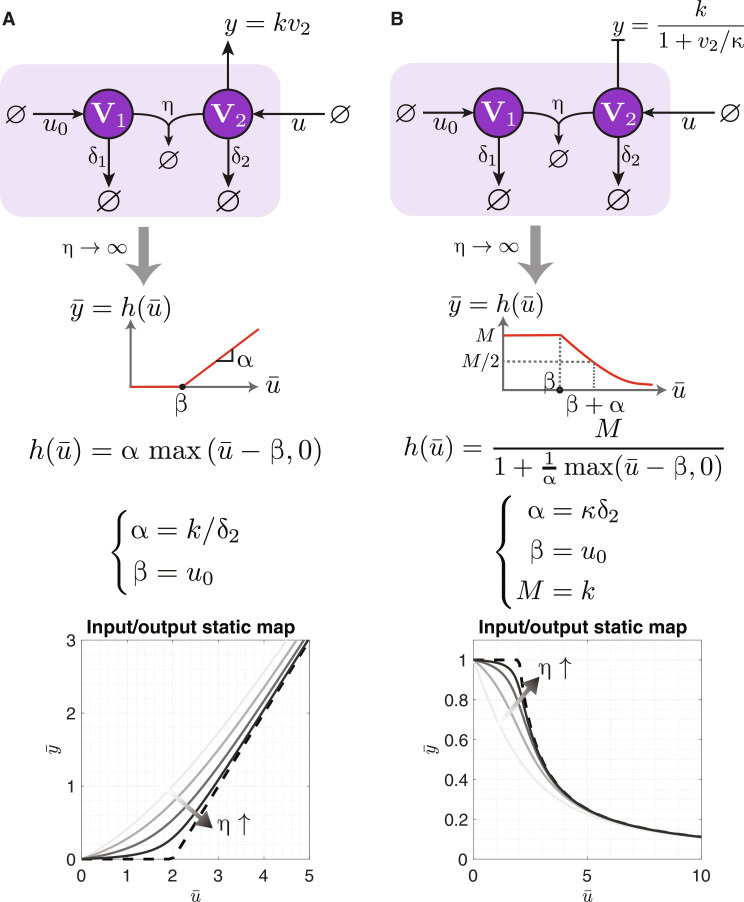
Biomolecular switching using sequestration motifs. The two sequestration networks in (**A**) and (**B**) consist of two species **V**_**1**_ and **V**_**2**_, which sequester each other at a rate η while degrading separately at rates δ_1_ and δ_2_. The input *u* corresponds to the production rate of **V**_**2**_, and the thresholds are determined by the production rate *u*_0_ of **V**_**1**_. The two networks differ in the role of **V**_**2**_ which acts as an activator in (A) and a repressor in (B). The bottom plots numerically confirm these designs.

To realize the three anti-windup strategies using CRNs, we first need to realize the switches depicted in Fig. 6A, which are mathematically represented as the monotonic functions *h*_1_ and *h*_2_. Specifically, we consider the functional forms shown in the gray box of Fig. 6A and reproduced in Fig. 7 for convenience. The sequestration networks depicted in Fig. 7 (A and B) deliver a steady-state response that approximates the desired functions. This approximation becomes exact in the limit of strong sequestration.

The two sequestration networks consist of two species **V**_**1**_ and **V**_**2**_ which sequester each other at a rate of η while separately degrading at rates δ_1_ and δ_2_, respectively. Here, the input, represented as *u*, signifies the production rate of **V**_**2**_, while the thresholds are determined by the production rate *u*_0_ of **V**_**1**_. The distinguishing feature between the two networks lies in the output: In Fig. 7A, **V**_**2**_ serves as an activator; conversely, in Fig. 7B, **V**_**2**_ functions as a repressor. Accordingly, the output is represented by the activation and repression rates, respectively. Once again, Theorems 1 and 2 are invoked here, and the derivations can be found in section S5. These designs are also numerically verified in the plots shown at the bottom of Fig. 7. Notably, decreasing the degradation rates yields a steeper slope leading to an ultrasensitive response (*27*, *55*)—a feature that is not required here for anti-windup.

#### CRN realizations of anti-windup topologies

**Fig. 8.**
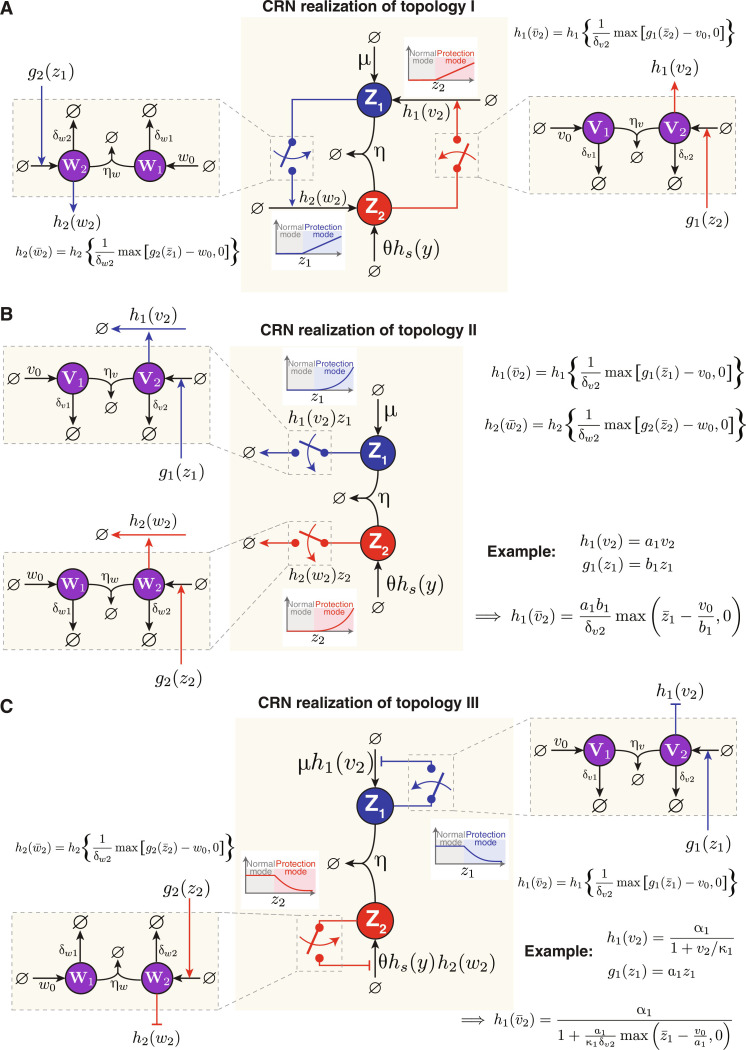
CRN realizations of three anti-windup topologies. (**A**) Topology I, (**B**) topology II, and (**C**) topology III. In each topology, sequestration networks, which consist of species (**V**_**1**_, **V**_**2**_) and (**W**_**1**_, **W**_**2**_) that sequester each other, are used to implement switch-like behaviors that intervene to prevent windup when necessary. Topology I uses activator switches and so *h*_1_ and *h*_2_ are monotonically increasing beyond the threshold. Topology II uses a similar mechanism, but the switches catalyze the self-degradations of **Z**_**1**_ and **Z**_**2**_. Here, *h*_1_(*v*_2_) and *h*_2_(*w*_2_) represent degradation rates that are monotonically increasing beyond the thresholds. The corresponding propensity functions, however, are given by *h*_1_(*v*_2_)*z*_1_ and *h*_2_(*w*_2_)*z*_2_, respectively. As a result, if *h*_1_ and *h*_2_ are linear functions beyond the threshold, then the corresponding propensities are quadratic. Last, topology III introduces repressor switches, and so, *h*_1_(*v*_2_) and *h*_2_(*w*_2_) monotonically decrease beyond the thresholds. Note that while the functions *h*_1_, *h*_2_, *g*_1_, and *g*_2_ are kept intentionally general in this depiction, specific examples are given in (B) and (C). In (B), the functions are presented in their linear form, while in panel (C), *h*_1_ takes on a Hill form.

Next, we leverage the sequestration-based switches to fully embody the three anti-windup topologies depicted in Fig. 6A as CRNs. Figure 8 (A to C) each represents a CRN corresponding to anti-windup topologies I, II, and III, respectively. Given the symmetry of the anti-windup schemes in **Z**_**1**_ and **Z**_**2**_, we describe the anti-windup operation on **Z**_**1**_ only.

For the CRN realization of topology I, depicted in Fig. 8A, the red switch is implemented using the sequestration network from Fig. 7A. This network consists of two species, **V**_**1**_ and **V**_**2**_, which sequester each other at a rate of η*_v_* and degrade separately at rates δ_*v*1_ and δ_*v*2_. The production rate of **V**_**2**_, represented as *g*_1_(*z*_2_), serves as the input to the switch and is a monotonically increasing function of *z*_2_. Its output, denoted by *h*_1_(*v*_2_), is a monotonically increasing function of the activator **V**_**2**_, which plays a role in the production rate of **Z**_**1**_. Therefore, with reference to Fig. 7A and section S5, assuming a high sequestration rate η*_v_*, $h_{1}\left({\bar{v}}_{2}\right)$ can be written as

$$h_{1}\left({\bar{v}}_{2}\right)=h_{1}\left\{\frac{1}{\delta_{v2}}\text{max}\left[g_{1}\left({\bar{z}}_{2}\right)-v_{0},0\right]\right\}$$ (27)

where *v*_0_ is the constant production rate of **V**_**1**_, serving as the threshold. Note that *h*_1_ and *g*_1_ are retained as arbitrary monotonically increasing functions for generality; however, linear representations of these functions would align exactly with the depiction in Fig. 6A, as shown in the example of Fig. 8B.

For the CRN realization of topology II, the blue switch is implemented using a similar sequestration network. The primary differences lie in the production rate of **V**_**2**_, which is now a function of *z*_1_ rather than *z*_2_, and **V**_**2**_ now catalyzes the degradation of **Z**_**1**_ at a rate *h*_1_(*v*_2_). Thus, the degradation propensity of **Z**_**1**_ is represented by *h*_1_(*v*_2_)*z*_1_. With reference to Fig. 7A and section S5, assuming a high sequestration rate η*_v_*, the degradation propensity at steady state can be expressed as

$$h_{1}\left({\bar{v}}_{2}\right){\bar{z}}_{1}=h_{1}\left\{\frac{1}{\delta_{v2}}\text{max}\left[g_{1}\left({\bar{z}}_{1}\right)-v_{0},0\right]{\bar{z}}_{1}\right\}$$ (28)

where the propensity now grows quadratically beyond the threshold *v*_0_ (see fig. S1B).

Last, for the CRN realization of topology III, the blue switch is realized using the sequestration network from Fig. 7B. The main distinction from topology II lies in **V**_**2**_, which now represses **Z**_**1**_ at a rate *h*_1_(*v*_2_). This rate is a monotonically decreasing function of *v*_2_, for instance, *h*(*v*_2_) = α_1_/(1 + *v*_2_/κ_1_).

#### Genetic realizations of anti-windup topologies

Genetic realizations of biomolecular integral controllers have already been successfully built and tested in both bacterial and mammalian cells (*1*). Therefore, we will not delve into the genetic realization of integral controllers here. Instead, our focus will be on proposing genetic realizations of anti-windup circuitry. The genetic implementations proposed in this study rely on inteins. An intein is a short protein segment that can autocatalytically excise itself from a protein structure and simultaneously rejoin the remaining segments, known as exteins (*56*). Split inteins, a subcategory of inteins, are divided into two parts commonly referred to as IntN and IntC. When active, these split inteins can heterodimerize and independently carry out protein splicing reactions, which involve the irreversible creation and destruction of peptide bonds in a strict one-to-one stoichiometric ratio. Leveraging their ability to exchange, cleave, or ligate amino acid sequences, split inteins provide the necessary foundation to realize the sequestration reactions that are crucial to the genetic realization of the anti-windup CRNs depicted in Fig. 8. The flexible and small-sized nature of split inteins (*57*), their existence in numerous orthogonal pairs (*58*), and their applicability across diverse life forms (*59*, *60*) make them particularly attractive for constructing genetic circuits. They have previously been used to build biomolecular integral controllers (*9*).

**Fig. 9.**
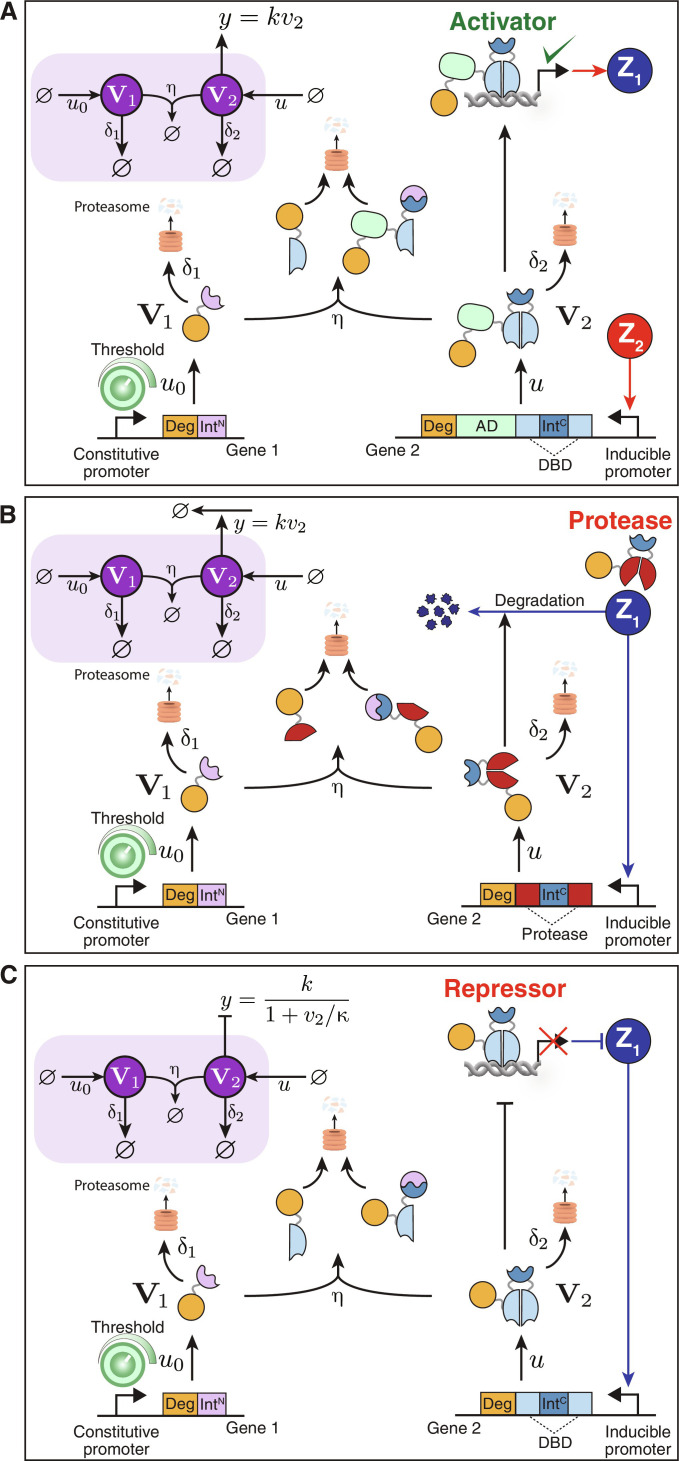
Intein-based genetic realizations of anti-windup topologies. (**A**) to (**C**) illustrate the genetic realizations for topologies I, II, and III, respectively. Each realization is achieved using two genes, one driven by a constitutive promoter and another by an inducible promoter. The proteins expressed by these genes, **V**_**1**_ and **V**_**2**_, engage in an intein-splicing reaction and are subsequently degraded. The function of **V**_**2**_ varies across the topologies, acting as an activator in (A), a protease in (B), and a repressor in (C). The strength of the constitutive promoter or plasmid copy number can be manipulated to adjust the anti-windup threshold. AD, activation domain; DBD, DNA binding domain; IntC/IntN, intein C/N; Deg, degradation tag. The protein domains are color-coded to match the genes from which they are expressed.

Figure 9 (A to C) depicts three genetic realizations compatible with topologies I, II, and III from Fig. 8, respectively. Given that the anti-windup circuitry is symmetric with respect to **Z**_**1**_ and **Z**_**2**_, our explanation here will focus on the genetic realization for one of them only. As depicted in Fig. 9A, the circuit is composed of two genes that express the proteins **V**_**1**_ and **V**_**2**_. The first gene is driven by a constitutive promoter and includes a degradation tag fused to IntN. The second gene, on the other hand, is driven by an inducible promoter activated by **Z**_**2**_. It consists of a DNA binding domain linked via an IntC and is fused to an activation domain and a degradation tag. Upon expression of the two genes, the corresponding proteins **V**_**1**_ and **V**_**2**_ engage in an intein-splicing reaction and are independently degraded by the proteasome. It is important to note that before splicing, **V**_**2**_ functions as an activator, driving the expression of **Z**_**2**_. However, its function is dismantled when it undergoes the splicing reaction with **V**_**1**_. The threshold of this anti-windup circuit can be adjusted by modifying the strength of the constitutive promoter or its plasmid copy number. It can also be tuned by inducers if the constitutive promoter is replaced by an inducible promoter. In contrast, the anti-windup circuit in Fig. 9C is the same as that in Fig. 9A but with only two differences: Gene 2 lacks an activation domain and its promoter is activated by **Z**_**1**_ instead of **Z**_**2**_. Consequently, **V**_**2**_ acts as a repressor rather than an activator, making this circuit configuration appropriate for implementing topology III. Last, the anti-windup circuit in Fig. 9B is similar to that in Fig. 9C, except for a single distinction: The DNA binding domain is replaced by a protease. This modification causes **V**_**2**_ to act as a protease that degrades **Z**_**1**_ rather than repressing its production.

### Numerical simulations

**Fig. 10.**
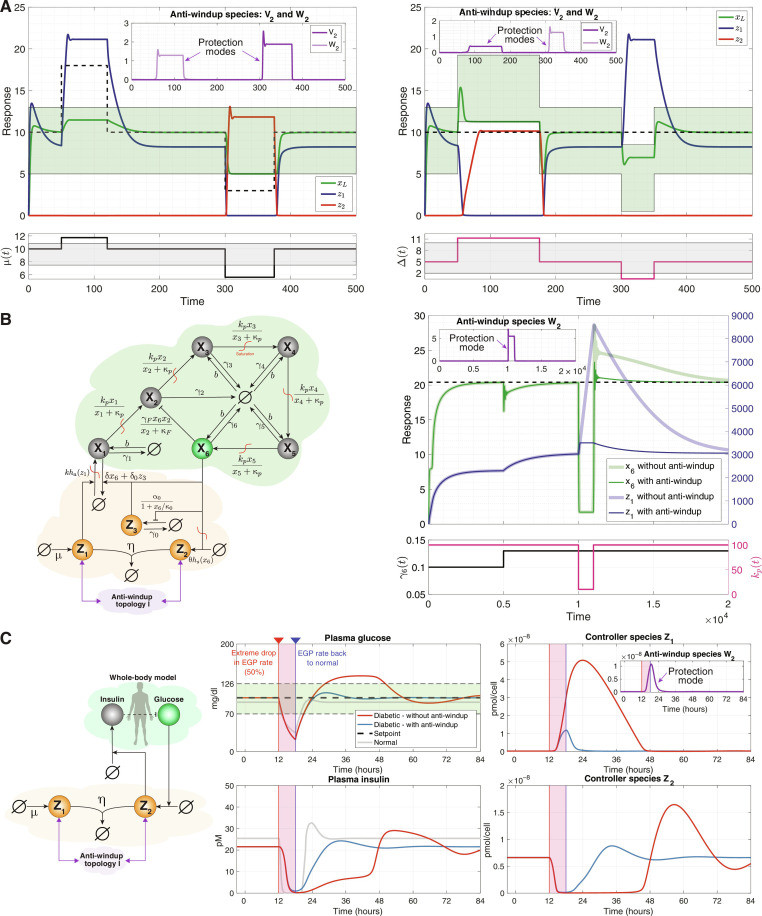
Simulations of anti-windup circuitry with three processes of increasing complexity. (**A**) The first simulation revisits the gene expression process, implementing the CRN realization of topology I to mitigate windup effects observed in Fig. 5 (C and D). Anti-windup parameters are η*_v_* = η*_w_* = 100, *v*_0_ = 10, *w*_0_ = 20. (**B**) The second simulation illustrates compatibility with biomolecular PID controllers [third-order PID (*22*)] with saturations to regulate a complex process of six species. The plots show improved dynamic performance of the output **X**_**6**_. Parameter values from (*22*) are used, with additional parameters introducing saturations, basal expressions, and anti-windup circuitry: μ = 6, *k* = 15, θ = 300, κ*_a_* = κ*_s_* = κ*_p_* = 1000, *b* = 0.2, *v*_0_ = *w*_0_ = 3500, and η*_v_* = η*_w_* = η = 10^4^. (**C**) The final simulation uses a whole-body model of patients with type I diabetes (*47*, *48*, *61*) as the controlled process. Windup effects are tested under a severe transient disturbance in the EGP rate. The gray response represents nondiabetic individuals not controlled by our circuits, while the blue and red responses represent patients with diabetes regulated by the AIF controller with and without anti-windup circuitry, respectively. With anti-windup circuitry, glucose levels return smoothly to the healthy range. Without it, **Z**_**1**_ accumulation inhibits **Z**_**2**_ and insulin, causing prolonged high glucose levels. Parameters are primarily from (*10*), except for the scaling of μ and θ by 100 to increase the controller’s speed. In addition, η*_v_* = η*_w_* = 10^4^ μmol^−1^ hour^−1^, and *v*_0_ = 100*w*_0_ = 0.01 μmol/hour. The inset plots across all panels show the dynamics of the anti-windup species which become active when thresholds are exceeded. We have δ_*v*_1__ = δ_*v*_2__ = δ_*w*_1__ = δ_*w*_2__ = 1, and *h*_1_, *h*_2_, *g*_1_, *g*_2_ from Fig. 8 are all identity functions.

To verify the effectiveness of our proposed anti-windup circuits, we undertake three numerical simulations using three processes of increasing complexity. Figure 10 depicts the simulations results for topology I only; however, similar results for topologies II and III can be found in figs. S4 and S5, respectively.

The first simulation, shown in Fig. 10A, revisits the gene expression process outlined in Example 3—a process that previously demonstrated windup effects as shown in Fig. 5 (C and D). Here, we use the CRN realization of topology I, shown in Fig. 8A, to mitigate these windup effects. As depicted in Fig. 10A, our anti-windup strategy successfully eliminates windup effects in both setpoint tracking (left) and disturbance rejection (right) scenarios. The inset figures track the dynamics of the anti-windup circuits **V**_**2**_ and **W**_**2**_, revealing that they remain dormant until the preset thresholds of **Z**_**1**_ and **Z**_**2**_ are surpassed. Upon reaching these thresholds, they become active and shift the controller into a protection mode that sacrifices RPA (which is anyway unattainable) for safety, thereby preventing **Z**_**1**_ and **Z**_**2**_ from reaching excessive levels that would cause windup and deteriorate dynamic performance.

Next, we illustrate that our proposed anti-windup topologies are compatible with biomolecular PID controllers. In this scenario, we consider a more complex process controlled by a third-order PID controller (*22*), as represented in the closed-loop network of Fig. 10B. This process consists of six species, **X**_**1**_ through **X**_**6**_, with **X**_**1**_ being the input species and **X**_**6**_ as the regulated output species of interest. Each species **X**_**i**_ degrades at a rate γ*_i_* and catalytically produces **X**_**i+1**_ for *i* = 1,2, ⋯,5. In addition, **X**_**6**_ actively degrades **X**_**2**_. This process is taken from (*22*, *23*) but with two modifications: Catalytic production reactions are changed to potentially saturating Hill functions rather than linear functions, and a basal expression rate *b* for all the species is added. The simulation results presented in Fig. 10B involve two different disturbances: a persistent but admissible disturbance in γ_6_, which the controller successfully rejects, and a transient but inadmissible disturbance in *k_p_* which is the maximum production rate of all species. The first disturbance could represent a mutation in the relevant gene’s coding sequence or the activation of another pathway that sequesters the output protein. The second disturbance could model a severe, transient metabolic burden that suppresses the production of all species within the regulated network. This intense, albeit brief, disturbance triggers windup as **Z**_**1**_ builds up to high levels, leading to poor dynamic performance even after the disturbance has passed. However, with the implementation of the anti-windup circuitry, the accumulation of **Z**_**1**_ is circumvented, resulting in superior dynamic performance after. Once again, the inset figure displays the dynamics of the relevant anti-windup species **W**_**2**_, which only activates during windup, transitioning the controller into a protection mode.

In the final simulation example, we use a highly complex process pertaining to a mathematical whole-body model of patients with type I diabetes (*47*, *48*, *61*). This model gave rise to the first computer simulator approved by the FDA as an alternative to preclinical trials and animal testing. Note that a prior simulation study demonstrated an effective control of this model using the AIF controller (*10*), where insulin production is the control action, and blood glucose level is the output required to exhibit RPA. We retain the same AIF closed-loop network and parameter values from (*10*), with one exception: We increase the parameters μ and θ by a factor of 100 to speed up the response while maintaining the same setpoint at 100 mg/dl—a value within the healthy range of [70,126] mg/dl. To test for windup effects, we introduce a severe but transient disturbance in the model. Specifically, the parameter *k*_*p*_1__ is reduced by 50% for a duration of 6 hours, simulating a marked change in endogenous glucose production (EGP), after which the EGP rate returns to normal. The gray curves in Fig. 10C correspond to the response of the model of a nondiabetic person not controlled by our circuits. These curves show that insulin secretion abruptly drops to zero to counteract the introduced severe disturbance and successfully returns the glucose level to the healthy range. It is worth noting that even in a model of a nondiabetic individual, plasma glucose transiently drops to a substantially low level, emphasizing the intensity of the applied disturbance. When we use the AIF controller without the anti-windup circuitry to control the model of a patient with diabetes, we get the response depicted in red. The accumulation of **Z**_**1**_ to high levels in response to the inadmissible disturbance is followed by a lengthy recovery time—a clear sign of windup. This leads to a prolonged period of low insulin secretion as the actuator species **Z**_**2**_ remains near zero, resulting in dangerously elevated glucose levels for around 20 hours. However, the implementation of our anti-windup topology I effectively mitigates this issue. The anti-windup species **W**_**2**_, shown in the inset figure, intervenes during the severe disturbance, preventing **Z**_**1**_ from accumulating and, therefore, avoiding windup. Consequently, glucose levels never overshoot the healthy range.

## DISCUSSION

The phenomenon of windup presents a substantial obstacle for any control system that aim to robustly regulate a target variable through integral feedback control. This challenge is especially evident in biomolecular and biomedical contexts where the limitations of actuators, sensors, and changes in processes may result in extended periods of poor performance or even loss of stability. With this in mind, our research focused on designing and evaluating biomolecular anti-windup strategies capable of mitigating windup effects and enhancing the robustness and reliability of biomolecular control systems.

In this study, we introduced three anti-windup topologies specifically adapted for biomolecular control systems that harness the RPA-achieving properties of the AIF controller and its PID extensions. These topologies were carefully designed to account for biomolecular constraints such as positivity, promoter saturation, or resource limitations. We initially introduced the topologies using a high-level phenomenological representation involving switches. Subsequently, a sequestration reaction–based CRN was introduced to realize the behavior of the switches. Sequestration motifs are a common fixture in biomolecular designs to realize numerous functionalities including subtraction modules (*62*), biomolecular input-output linear systems (*63*), biomolecular integral control (*6*), biomolecular derivative operators (*64*, *65*, *66*), PID controllers as CRNs (*22*, *67*, *68*, *69*), band-pass filters (*70*), achieving ultrasensitive response (*27*), nonlinear activation functions for biomolecular neural networks (*71*), and stabilization near unstable equilibria (*72*), among others. This is predominantly attributed to the capability of sequestration reactions to perform comparison operations. In our work, we harnessed this characteristic to realize biomolecular anti-windup strategies, further underlining the essential role of sequestration motifs in biomolecular circuit design.

By leveraging Theorems 1 and 2, we established a connection between these biomolecular topologies and classical control-theoretic concepts. This association was made by calculating transfer functions and block diagrams that are exact in the limit of strong sequestration rates. This approach differs from previous approximation methods that relied on linearizations around the fixed point (*22*, *23*, *66*, *69*). Specifically, in this study, we exploit timescale separation which has previously been used to derive reduced order models describing the dynamics of biomolecular controllers (*20*, *73*–*76*). It is noteworthy to point out that in (*73*), a theorem similar to Theorem 1 is presented. Samaniego *et al.* (*73*) establish that if there exists some time *T* > 0 before which the concentrations of the sequestration pair do not cross, then simple sequestration networks reduce to subtraction operations for all *t* ≥ *T*. In contrast, Theorem 1 establishes a different model reduction result that is valid for more general networks involving a fast sequestration reaction and under less restrictive conditions. The model reduction results in Theorems 1 and 2 are valid for the dynamics over any compact time interval and for the fixed points, and they require no assumptions except Lipschitz continuity. In a sense, the two approaches complement each other since (*73*) addresses *t* ≥ *T* assuming that there exists a *T* that satisfies certain conditions; whereas Theorem 1 addresses *t* ∈ [0, *T*] for any *T* ≥ 0.

The promising potential of our methods to mitigate windup was illustrated both theoretically and through numerical simulations. Our simulations spanned three increasingly complex processes, thereby demonstrating the versatility and robustness of the proposed anti-windup topologies. From a basic gene expression process modeling transcription saturation to a highly intricate whole-body model of a patient with type I which is FDA approved, our topologies succeeded in preventing windup and maintaining effective control. We also put forth genetic designs capable of implementing the anti-windup strategies, based on inteins which were previously used to successfully build integral controllers. Thanks to their flexibility and small size, inteins hold considerable potential for use in various biomolecular circuits that involve sequestration reactions, such as those in our anti-windup designs.

One primary limitation of the introduced biomolecular anti-windup strategies involves the potential saturation of the anti-windup circuitry itself. This issue can arise from saturation of relevant promoters or constraints due to limited shared resources. As demonstrated in the numerical simulations (particularly the inset figures in Fig. 10), the anti-windup species remain inactive as long as the controller operates within the preset thresholds of normal integral mode. Only when these thresholds are surpassed do the anti-windup species become active to mitigate windup. As such, the overall system benefits from the favourable property of low burden imposed by the added anti-windup circuitry, as long as no windup occurs. It is worth mentioning that, when properly tuned, the burden imposed by the anti-windup circuitry is minimal but not zero, as the molecules implementing the mechanism are still produced even in the absence of windup. However, if not properly tuned, then this intervention itself may saturate, compromising the effectiveness of the augmented anti-windup circuitry. Nevertheless, even in such a case, the dynamic range of the integrator is expanded compared to a system without anti-windup circuitry, thereby still reducing the impact of windup. In scenarios where the imposed burden is excessive, it may be necessary to distribute the AIF controller and the anti-windup circuitry across multiple cellular populations, following the approach suggested in (*29*, *30*). It is important to note that limited shared resources may lead to saturations affecting various network components such as reference, sensing, actuation, and anti-windup reactions and may also couple the dynamics among these components. A simulation study is presented in figs. S2 and S3 to demonstrate that, even with additional burden imposed by resource sharing, the anti-windup reactions ensure well-behaved dynamics. It is also analytically shown in the section S6 that the symmetry of the sequestration motif used to realize the switches in the anti-windup circuitry provides two crucial properties: (i) It enhances the robustness of the threshold parameter against the influence of shared resources, and (ii) it facilitates tuning the threshold which is managed through a production reaction analogous to the setpoint tuning in the AIF controller [see (*27*) and Box 1 for a similar discussion on threshold tuning]. These properties are particularly useful as the threshold is an important parameter that separates the normal mode from the anti-windup protection mode.

For the effective implementation of the anti-windup switches depicted in Fig. 6A via sequestration reactions, strong sequestrations are paramount. While milder sequestrations can still curtail windup, they introduce steady-state errors even under normal operations (refer to fig. S6). To address this, designers should ensure that sequestration reactions occur more rapidly than other reactions. This is expected to be observed with inteins, where intein-splicing reactions are considerably faster than gene expression processes (*77*).

Moving forward, the proposed anti-windup topologies represent a promising advancement toward more robust biomolecular control systems. The genetic implementation of these topologies could offer powerful tools for a range of applications, from gene regulation to personalized therapeutics. Moreover, the broader approach of designing safe control systems with consideration for the unique features and constraints of biomolecular processes holds potential to forge previously unidentified paths in the design of robust and effective biomolecular control systems. A potential avenue for future exploration could involve examining the impacts of windup phenomena and the corresponding anti-windup strategies in a stochastic context.

## MATERIALS AND METHODS

All simulations were performed using MATLAB. The MATLAB code for generating these simulations is available at the following Github repository https://github.com/Maurice-Filo/Biomolecular-Antiwindup-Circuits. Deterministic dynamics were simulated using the ODE23s solver, while the stochastic simulations in the Supplementary Materials were conducted using the Gillespie algorithm (*78*). The simulation of the glucose response in Fig. 10C was carried out using the SimBiology toolbox in MATLAB. Proofs of the theorems and lemmas are provided in the Supplementary Materials.

## Supplementary Materials

### This PDF file includes:

Supplementary Text Figs. S1 to S8 References
