# Supplementary material for: Anti-windup strategies for biomolecular control systems facilitated by model reduction theory for sequestration networks
Source: Sci Adv. 2024 Aug 21;10(34):eadl5439. doi: 10.1126/sciadv.adl5439 (PMC11338268; doi:10.1126/sciadv.adl5439)
Supplement: Supplementary file 1 — Supplementary Text Figs. S1 to S8 References [file sciadv.adl5439_sm.pdf]

Science Advances
AAAS

Supplementary Materials for

Anti-windup strategies for biomolecular control systems facilitated by model reduction theory for sequestration networks

Maurice Filo *et al.*

Corresponding author: Mustafa Khammash, mustafa.khammash@bsse.ethz.ch

Sci. Adv. **10**, eadl5439 (2024)
DOI: 10.1126/sciadv.adl5439

This PDF file includes:

Supplementary Text
Figs. S1 to S8
References

S1 The Model Reduction result for sequestration-based CRNs (deterministic setting)

In this section we prove a novel model reduction result for a deterministic Chemical Reaction Network (CRN) that includes a *fast* sequestration reaction of the form

$$\mathbf{Z}_1 + \mathbf{Z}_2 \xrightarrow{\eta} \varnothing,$$

with mass-action kinetics. Here $\mathbf{Z}_1$ and $\mathbf{Z}_2$ are the biomolecular species that sequester or annihilate each other at rate η and we are interested in the limiting dynamics as $\eta \to \infty$. As mentioned in the main paper, the ODEs and initial conditions for the pre-limiting system S_η and the limiting system $\mathcal{S}$ are given by

$$\mathcal{S}_\eta : \begin{cases} \dot{x}^{(\eta)} = F\left(x^{(\eta)}, z_1^{(\eta)}, z_2^{(\eta)}\right); & x^{(\eta)}(0) = x_0 \\ \dot{z}_1^{(\eta)} = W_1\left(x^{(\eta)}, z_1^{(\eta)}, z_2^{(\eta)}\right) - \eta z_1^{(\eta)} z_2^{(\eta)}; & z_1^{(\eta)}(0) = a \\ \dot{z}_2^{(\eta)} = W_2\left(x^{(\eta)}, z_1^{(\eta)}, z_2^{(\eta)}\right) - \eta z_1^{(\eta)} z_2^{(\eta)}; & z_2^{(\eta)}(0) = b, \end{cases} \qquad \mathcal{S} : \begin{cases} \dot{x} = F(x, z^+, z^-); & x(0) = x_0 \\ \dot{z} = W_1(x, z^+, z^-) - W_2(x, z^+, z^-); & z(0) = a - b, \end{cases} \tag{S1}$$

where $x_0 \in \mathbb{R}_+^L,\ a, b, \eta \in \mathbb{R}_+$ and the following notation is adopted

$$z^+ \triangleq \max(z, 0) \quad \text{and} \quad z^- \triangleq \max(-z, 0). \tag{S2}$$

The three functions W_1, W_2 and F meet the following criterion which is stated as Assumption 3 in the main text.

Assumption S1 *The three functions $W_1, W_2 : \mathbb{R}_+^{L+2} \to \mathbb{R}$ and $F : \mathbb{R}_+^{L+2} \to \mathbb{R}^L$ are assumed to be* globally Lipschitz *on their domains and their form is such that the solution of $\mathcal{S}_\eta$ lies in $\mathbb{R}_+^{L+2}$.*

Our main model reduction result in the deterministic setting is given by Theorem 1 in the main text, which we state here again for convenience. A numerical validation of this result in presented in Figure S7.

Theorem S1 *Under Assumption S1 and as $\eta \to \infty$, $\mathcal{S}_\eta$ converges to $\mathcal{S}$ in the following senses: $\forall T > 0$ we have*

$$\begin{aligned} &\lim_{\eta\to\infty} \sup_{t\in[0,T]} \left\| \left(x^{(\eta)}(t), z_1^{(\eta)}(t) - z_2^{(\eta)}(t)\right) - \left(x(t), z(t)\right) \right\| = 0, \\ \text{and} \qquad &\lim_{\eta\to\infty} \int_0^T \left\| \left(x^{(\eta)}(t), z_1^{(\eta)}(t), z_2^{(\eta)}(t)\right) - \left(x(t), z^+(t), z^-(t)\right) \right\| dt = 0, \end{aligned} \tag{S3}$$

where $\left(x^{(\eta)}(t), z_1^{(\eta)}(t), z_2^{(\eta)}(t)\right)$ is the unique solution to system $\mathcal{S}_\eta$ and $(x(t), z(t))$ is the unique solution to system $\mathcal{S}$.

Observe that the system $\mathcal{S}_\eta$, operates on two distinct timescales: a *fast* timescale of order η, where only the state components $z_1^{(\eta)}(t)$ and $z_2^{(\eta)}(t)$ evolve, and a *slow* timescale of order 1, where all the state components evolve. When the system is perturbed by reactions at the slow timescale, the fast sequestration induces rapid changes in $z_1^{(\eta)}(t)$ and $z_2^{(\eta)}(t)$ until a new equilibrium is reached, rendering the sequestration mechanism no longer fast (i.e. the rate is order 1). This phenomenon is called the *boundary layer effect* in singular perturbation theory. Due to the swift yet transient fluctuations it induces in $z_1^{(\eta)}(t)$ and $z_2^{(\eta)}(t)$, the convergence of the full dynamics $X^{(\eta)}(t) \triangleq \left(x^{(\eta)}(t), z_1^{(\eta)}(t), z_2^{(\eta)}(t)\right)$ to the dynamics of the reduced system $\mathcal{S}$ only occurs weakly, in the sense of L_1 or time-integral convergence, as indicated by the second convergence relation in Equation (S3). However, if we only consider the dynamics of the difference $z_1^{(\eta)}(t) - z_2^{(\eta)}(t)$ (along with $x^{(\eta)}(t)$), then the rapid fluctuations in $z_1^{(\eta)}(t)$ and $z_2^{(\eta)}(t)$ cancel out and we obtain a strong uniform convergence (over compact time-intervals) of the dynamics of $\mathcal{S}_\eta$ to the dynamics of the reduced model $\mathcal{S}$, as indicated by the first convergence relation in Equation (S3).

It may be tempting to cast the equations for $\mathcal{S}_\eta$ in the standard singular perturbation form, and apply model reduction results from this theory, such as the well-known Tikhonov's Theorem (see Theorem 11.1 in [82]). However this does not work

in our setting because of some subtle issues that we now discuss. Let us introduce the variable transformation $(z_1, z_2) \mapsto (z, y)$ where

$$z = \frac{z_1 - z_2}{2} \quad \text{and} \quad y = \frac{z_1 + z_2}{2}.$$

Then we can equivalently express the dynamics of $\mathcal{S}_\eta$ in terms of variables $(x^{(\eta)}, z^{(\eta)}, y^{(\eta)})$ as

$$\begin{aligned} \dot{x} &= F(x, y+z, y-z) \\ \dot{z} &= \frac{W_1(x, y+z, y-z) - W_2(x, y+z, y-z)}{2} \\ \frac{1}{\eta}\dot{y} &= \frac{W_1(x, y+z, y-z) + W_2(x, y+z, y-z)}{2\eta} - y^2 + z^2, \end{aligned}$$

where we drop the superscript $^{(\eta)}$ for convenience. This is the standard singular perturbation form with positive parameter $\epsilon := 1/\eta$ which multiplies the time-derivative for the fast variable y in the third ODE above (see Chapter 11 in [82]). Notice that in the limit $\epsilon \to 0$, this ODE degenerates into the algebraic relation $g(x, z, y) := -y^2 + z^2 = 0$ which essentially implies that $y = \pm z$ or simply $y = |z|$ as y cannot be negative (since it is the average concentration of $\mathbf{Z}_1$ and $\mathbf{Z}_2$). The absolute value function $|z|$ introduces non-differentiability at $z = 0$ in the ODEs for the slow variables x and z, violating one of the main assumptions of the Tikhonov's Theorem. Even if we ignore this issue, another issue arises due to the dynamics of the boundary layer system which describes the deviation of the fast variable y from its quasi steady-state $|z|$. Letting $\widetilde{y} = y - |z|$, this boundary layer system is given by

$$\dot{\widetilde{y}} = g(x, z, \widetilde{y} + |z|) = -(\widetilde{y} + |z|)^2 + z^2 = -\widetilde{y}(\widetilde{y} + 2|z|).$$

It is easy to check that this system is not uniformly exponentially stable, as required by the Tiknonov's Theorem.

These issues highlight the challenges in proving the model reduction result (i.e. Theorem S1). A primary theoretical contribution of this study is to present a tailored strategy for effectively addressing these challenges within the context of sequestration-based CRNs. The preceding discussion highlights a key issue arising when $z = 0$ or $z_1 = z_2$. To address this issue, we segment each trajectory into two distinct types: one for dynamics until it reaches an ϵ neighborhood of $z_1 = z_2$, and the other for dynamics until it exits a 2ϵ neighborhood of $z_1 = z_2$. We establish an error bound for each segment type, comparing the pre-limiting and reduced models. Leveraging the alternating nature of these segment types until the final time $T > 0$ is reached, we suitably combine the error estimates to prove Theorem S1. In order to facilitate readability, we divide the proof of Theorem S1 into several steps which we now briefly describe:

- We first analytically compute the trajectories of the (z_1, z_2) subsystem evolving under the sequestration reaction and show that the steady-state is essentially the positive and negative parts of the initial difference between z_1 and z_2. Moreover, irrespective of the initial state, the deviation between the variables and their steady-states is the same at all times for both z_1 and z_2 (see Lemma S1 and Remark S1).
- We then show boundedness of the trajectory over compact time-intervals using Gronwall's inequality (see Lemma S2).
- Next we segment the trajectory into two types, as indicated above, and estimate the error accumulated by each type (see Propositions S1 and S2). The two errors are then combined into a single bound in Lemma S3. The proof of these propositions relies on Lemma S2 and also we make explicit use of the analytical computation in Lemma S1 to cancel out the boundary layer effects.
- Finally, we aggregate the errors bounds we obtain from Lemma S3 over all the segments to prove Theorem S1.

Henceforth, for any two numbers a and b, we denote their minimum by $a \wedge b$.

Lemma S1 *Let $a, b \in \mathbb{R}_+$ and let $\Psi_t(a, b) \triangleq \big(z_1(t), z_2(t)\big)$ be the solution of the following initial value problem*

$$\begin{cases} \dot{z}_1 = -z_1 z_2; & z_1(0) = a \\ \dot{z}_2 = -z_1 z_2; & z_2(0) = b. \end{cases}$$

Then for any $t \geq 0$, we can express $\Psi_t(a,b)$ as

$$\Psi_t(a,b) = \begin{cases} \left(\frac{a(a-b)}{a-be^{-(a-b)t}}, \frac{b(a-b)e^{-(a-b)t}}{a-be^{-(a-b)t}}\right) & \text{if} \quad a > b \\ \left(\frac{a(b-a)e^{-(b-a)t}}{b-ae^{-(b-a)t}}, \frac{b(b-a)}{b-ae^{-(b-a)t}}\right) & \text{if} \quad a < b \\ \left(\frac{a}{1+at}, \frac{a}{1+at}\right) & \text{if} \quad a = b. \end{cases}$$

Remark S1 *Note that as $t \to \infty$, we get*

$$\Phi(a,b) \triangleq \lim_{t\to\infty} \Psi_t(a,b) = \left((a-b)^+, (a-b)^-\right).$$

Moreover for any $t \geq 0$, we have

$$\Psi_t(a,b) - \Phi(a,b) = \begin{cases} \frac{b(a-b)e^{-(a-b)t}}{a-be^{-(a-b)t}}\,(1,1) & \text{if} \quad a > b \\ \frac{a(b-a)e^{-(b-a)t}}{b-ae^{-(b-a)t}}\,(1,1) & \text{if} \quad a < b \\ \frac{a}{1+at}\,(1,1) & \text{if} \quad a = b, \end{cases} \tag{S4}$$

and so the two components of $\Psi_t(a,b) - \Phi(a,b)$ are identical for all $t \geq 0$.

Proof. We shall first prove Lemma S1 in the case $a > b$, hence $\alpha := (a-b) > 0$. Observe that

$$\frac{d(z_1 - z_2)}{dt} = \dot{z}_1 - \dot{z}_2 = 0 \qquad \Longrightarrow \qquad z_1(t) - z_2(t) = z_1(0) - z_2(0) = \alpha.$$

Therefore we can write $z_2(t) = z_1(t) - \alpha$. Substituting this for z_2 we obtain

$$\dot{z}_1 = -z_1(z_1 - \alpha), \tag{S5}$$

which implies that

$$-\alpha \frac{dz_1}{z_1(z_1-\alpha)} = \left(\frac{1}{z_1} - \frac{1}{z_1-\alpha}\right) dz_1 = \alpha dt.$$

Integrating both sides and using $\big(z_1(0), z_2(0)\big) = (a,b)$ we get

$$\log\left(\frac{z_1(t)}{z_1(t)-\alpha}\right) = \log\left(\frac{a}{b}\right) + \alpha t,$$

which upon exponentiation gives us

$$\frac{z_1(t)}{z_1(t)-\alpha} = \left(\frac{a}{b}\right) e^{\alpha t}.$$

Solving for $z_1(t)$ and setting $z_2(t) = z_1(t) - \alpha$ proves the lemma in the case $a > b$. The case $a < b$ follows by symmetry and so we now consider the case $a = b$. It is immediate that in this case $z_1(t) = z_2(t)$ for all $t \geq 0$, and solving the ODE (S5) yields

$$z_1(t) = z_2(t) = \frac{a}{1+at}.$$

This completes the proof of this lemma. □

The following lemma establishes that for any given $\eta > 0$, the solution of the system $\mathcal{S}_\eta$ remains bounded over finite time periods.

Lemma S2 *Let $X^{(\eta)}(t) \triangleq \left(x^{(\eta)}(t), z_1^{(\eta)}(t), z_2^{(\eta)}(t)\right)$ be the solution of system $\mathcal{S}_\eta$. Then, $\forall T > 0$, $\exists M_0(T) > 0$ such that*

$$\sup_{t\ \in[0,T]} \sup_{\eta} \|X^{(\eta)}(t)\| \leq M_0(T).$$

Proof. From (S1) we see that $X^{(\eta)}(t)$ satisfies

$$X^{(\eta)}(t) = X^{(\eta)}(0) + \int_0^t \left(F\left(X^{(\eta)}(s)\right), W_1\left(X^{(\eta)}(s)\right) - \eta z_1^{(\eta)}(s) z_2^{(\eta)}(s), W_2\left(X^{(\eta)}(s)\right) - \eta z_1^{(\eta)}(s) z_2^{(\eta)}(s) \right) ds.$$

Since the components of $X^{(\eta)}(t)$ are always positive the following inequality holds componentwise

$$X^{(\eta)}(t) \le X^{(\eta)}(0) + \int_0^t \left(F\left(X^{(\eta)}(s)\right), W_1\left(X^{(\eta)}(s)\right), W_2\left(X^{(\eta)}(s)\right) \right) ds.$$

As functions F, W_1 and W_2 are Lipchitz on $\mathbb{R}_+^{L+2}$, there exists a constant $M \ge 0$ such that

$$\left\| \left(F\left(X^{(\eta)}(t)\right), W_1\left(X^{(\eta)}(t)\right), W_2\left(X^{(\eta)}(t)\right) \right) \right\| \le M \left\| X^{(\eta)}(t) \right\| + \left\| \left(F(0), W_1(0), W_2(0) \right) \right\|, \quad \forall t \in [0, T],$$

where the triangle inequality is employed. This allows us to obtain

$$\begin{aligned} \left\| X^{(\eta)}(t) \right\| &\le \left\| X^{(\eta)}(0) \right\| + \int_0^t \left(M \left\| X^{(\eta)}(s) \right\| + \left\| \left(F(0), W_1(0), W_2(0) \right) \right\| \right) ds \\ &\le \|(x_0, a, b)\| + \left\| \left(F(0), W_1(0), W_2(0) \right) \right\| t + M \int_0^t \left\| X^{(\eta)}(s) \right\| ds. \end{aligned}$$

Applying Gronwall's inequality proves Lemma S2 with $M_0(T) \triangleq \left(\|(x_0, a, b)\| + \left\| \left(F(0), W_1(0), W_2(0) \right) \right\| T \right) e^{MT}$. □

Next, we examine two specific time instants of particular importance. The first time instant, represented by $\sigma_\eta(\epsilon)$, marks the first moment when z_1 and z_2 approach within an ϵ-close neighborhood. The second time instant, denoted by $\mu_\eta(\epsilon)$, indicates the first moment when z_1 and z_2 depart from each other's 2ϵ-neighborhood. It should be noted that while 2ϵ is selected here for simplicity, any value $k\epsilon$ could be chosen, provided $k > 1$.

Proposition S1 *Let $X^{(\eta)}(t) \triangleq \left(x^{(\eta)}(t), z_1^{(\eta)}(t), z_2^{(\eta)}(t) \right)$ be the solution of system $\mathcal{S}_\eta$, but with initial condition $X^{(\eta)}(0)$ satisfying $\lim_{\eta\to\infty} X^{(\eta)}(0) = (x_0, \epsilon, 0)$ for some $x_0 \in \mathbb{R}_+^L$ and $\epsilon > 0$. Furthermore, let $\left(x(t), z(t) \right)$ be the solution of system $\mathcal{S}$, but with initial condition $\left(x(0), z(0) \right) = (x_0, \epsilon)$. Define*

$$\mu_\eta(\epsilon) \triangleq \inf \left\{ t \ge 0 : \left| z_1^{(\eta)}(t) - z_2^{(\eta)}(t) \right| \ge 2\epsilon \right\}.$$

Then we have

$$\liminf_{\eta\to\infty} \mu_\eta(\epsilon) > 0, \tag{S6}$$

and for any $T > 0$, there exists two constants $C_1, C_2 > 0$ such that

$$\limsup_{\eta\to\infty} \frac{\sup_{t\in[0,\mu_\eta(\epsilon)\wedge T]} \left\| \left(x^{(\eta)}(t), z_1^{(\eta)}(t) - z_2^{(\eta)}(t) \right) - \left(x(t), z(t) \right) \right\|}{(\mu_\eta(\epsilon) \wedge T) e^{C_1(\mu_\eta(\epsilon)\wedge T)}} \le C_1 \epsilon \tag{S7}$$

$$\limsup_{\eta\to\infty} \frac{\int_0^{\mu_\eta(\epsilon)\wedge T} \left\| X^{(\eta)}(t) - \left(x(t), z^+(t), z^-(t) \right) \right\| dt}{\mu_\eta(\epsilon) \wedge T} \le C_2 \epsilon. \tag{S8}$$

Remark S2 *By symmetry, the proposition also holds with initial conditions $\lim_{\eta\to\infty} X^{(\eta)}(0) = (x_0, 0, \epsilon)$ for $\mathcal{S}_\eta$ and $\left(x(0), z(0) \right) = (x_0, -\epsilon)$ for $\mathcal{S}$. It will be clear in the proof that the proposition also holds if $\lim_{\eta\to\infty} X^{(\eta)}(0) = (x_0, a, b) \in \mathbb{R}_+^{L+2}$ and $\left(x(t), z(t) \right) = (x_0, a - b)$, where either $a \le \epsilon$ and $b = 0$ or $a = 0$ and $b \le \epsilon$.*

Proof. Without loss of generality we can assume that $X^{(\eta)}(0) = (x_0, \epsilon, 0)$ for each η. Observe that

$$\frac{d}{dt}\left(z_1^{(\eta)}(t) - z_2^{(\eta)}(t)\right) = W_1\left(X^{(\eta)}(t)\right) - W_2\left(X^{(\eta)}(t)\right),$$

and since $z_1^{(\eta)}(0) - z_2^{(\eta)}(0) = \epsilon$, the definition of $\mu_\eta(\epsilon)$ implies that

$$2\epsilon = \left|\epsilon + \int_0^{\mu_\eta(\epsilon)} \left[W_1\left(X^{(\eta)}(t)\right) - W_2\left(X^{(\eta)}(t)\right)\right] dt\right|.$$

Using Lemma S2 and the Lipchitz conditions on functions W_1 and W_2, we can find a constant $W_0 > 0$ satisfying

$$\sup_{t\in[0,T]} \sup_{\eta} \left|W_1\left(X^{(\eta)}(t)\right) - W_2\left(X^{(\eta)}(t)\right)\right| \le W_0.$$

This ensures that $\mu_\eta(\epsilon) \ge \epsilon/W_0$ and proves (S6). Next, define a function $\bar{F}$ by transforming the function F as follows

$$\bar{F}(x, z_1, z_2) \triangleq F\left(x, \frac{z_1 + z_2}{2}, \frac{z_2 - z_1}{2}\right).$$

Also define the functions $\bar{W}_1$ and $\bar{W}_2$ by similarly transforming functions W_1 and W_2, respectively. Let

$$\chi^{(\eta)}(t) \triangleq \left(x^{(\eta)}(t), v^{(\eta)}(t), w^{(\eta)}(t)\right), \quad \text{where} \begin{cases} v^{(\eta)}(t) \triangleq z_1^{(\eta)}(t) - z_2^{(\eta)}(t) \\ w^{(\eta)}(t) \triangleq z_1^{(\eta)}(t) + z_2^{(\eta)}(t). \end{cases}$$

One can see that $\chi^{(\eta)}(t)$ satisfies the following system of ODEs

$$\begin{cases} \dot{x} = \bar{F}(x, v, w); & x(0) = x_0 \\ \dot{v} = \bar{W}_1(x, v, w) - \bar{W}_2(x, v, w); & v(0) = \epsilon \\ \dot{w} = \bar{W}_1(x, v, w) + \bar{W}_2(x, v, w) - \frac{1}{2}\eta(w^2 - v^2); & w(0) = \epsilon. \end{cases}$$

Moreover $\big(x(t), z(t)\big)$ satisfies the following system of ODEs

$$\begin{cases} \dot{x} = \bar{F}(x, z, |z|); & x(0) = x_0 \\ \dot{z} = \bar{W}_1(x, z, |z|) - \bar{W}_2(x, z, |z|); & z(0) = \epsilon. \end{cases}$$

Note that $v^{(\eta)}(0) = \epsilon$, and we can express $\mu_\eta(\epsilon)$ as

$$\mu_\eta(\epsilon) = \inf\left\{t \ge 0 : \left|v^{(\eta)}(t)\right| \ge 2\epsilon\right\}.$$

As before, we can find a constant W_0 such that for any η we have

$$\sup_{t\in[0,T]} \left|W_1\left(X^{(\eta)}(t)\right) + W_2\left(X^{(\eta)}(t)\right)\right| \le W_0.$$

This ensures that in the time interval $[0, \mu_\eta(\epsilon))$, the differential inequality

$$\dot{w} \le W_0 + 2\eta\epsilon^2 - \frac{1}{2}\eta w^2; \quad w(0) = \epsilon,$$

is satisfied by $w^{(\eta)}(t)$. This means that in this time interval, we have $w^{(\eta)}(t) \le \gamma(t)$ where $\gamma(t)$ satisfies

$$\dot{\gamma} = W_0 + 2\eta\epsilon^2 - \frac{1}{2}\eta\gamma^2; \quad \gamma(0) = \epsilon.$$

Solving this initial value problem, we obtain

$$\gamma(t) = c\left[\frac{(\epsilon+c)+(\epsilon-c)e^{-\eta ct}}{(\epsilon+c)-(\epsilon-c)e^{-\eta ct}}\right], \qquad \text{with } c = 2\epsilon\sqrt{1+\frac{W_0}{2\epsilon^2\eta}}.$$

This shows that

$$\limsup_{\eta\to\infty} \frac{1}{\mu_\eta(\epsilon)\wedge T}\int_0^{\mu_\eta(\epsilon)\wedge T} |w^{(\eta)}(t)|dt \leq 2\epsilon. \tag{S9}$$

One can see that

$$\Big(x^{(\eta)}(t), v^{(\eta)}(t)\Big) - \Big(x(t), z(t)\Big) =$$
$$\int_0^t \left(\bar{F}\left(x^{(\eta)}(s), v^{(\eta)}(s), w^{(\eta)}(s)\right) - \bar{F}\Big(x(s), z(s), |z(s)|\Big), \bar{W}\left(x^{(\eta)}(s), v^{(\eta)}(s), w^{(\eta)}(s)\right) - \bar{W}\Big(x(s), z(s), |z(s)|\Big)\right) ds,$$

where $\bar{W}(x,v,w) = \bar{W}_1(x,v,w) - \bar{W}_2(x,v,w)$. This integral relation along with Lipchitz conditions on functions $\bar{F}$ and $\bar{W}$ implies that there exists a constant $M > 0$ such that

$$\left\|\Big(x^{(\eta)}(t), v^{(\eta)}(t)\Big) - \Big(x(t), z(t)\Big)\right\| \leq M\int_0^t \left(\left\|x^{(\eta)}(s) - x(s)\right\| + \left|v^{(\eta)}(s) - z(s)\right| + \left|w^{(\eta)}(s) - |z(s)|\right|\right) ds.$$

But $\left|w^{(\eta)}(s) - |z(s)|\right| \leq \left|w^{(\eta)}(s) - |v^{(\eta)}(s)|\right| + \left||v^{(\eta)}(s)| - |z(s)|\right| \leq \left|w^{(\eta)}(s)\right| + \left|v^{(\eta)}(s) - z(s)\right|$, because $w^{(\eta)}(s) \geq \left|v^{(\eta)}(s)\right|$ and $\Big||a| - |b|\Big| \leq \Big|a - b\Big|$ for any $a, b \in \mathbb{R}$. For any $t \in [0, \mu_\eta(\epsilon)\wedge T]$ and large η this allows us to write

$$\begin{aligned}\left\|\Big(x^{(\eta)}(t), v^{(\eta)}(t)\Big) - \Big(x(t), z(t)\Big)\right\| &\leq M\int_0^t \left(\left\|x^{(\eta)}(s) - x(s)\right\| + \left|v^{(\eta)}(s) - z(s)\right| + \left|w^{(\eta)}(s) - |z(s)|\right|\right) ds \\ &\leq M\int_0^t \left|w^{(\eta)}(s)\right| ds + 2M\int_0^t \left\|\Big(x^{(\eta)}(s), v^{(\eta)}(s)\Big) - \Big(x(s), z(s)\Big)\right\| ds \\ &\leq 2M\epsilon(\mu_\eta(\epsilon)\wedge T) + 2M\int_0^t \left\|\Big(x^{(\eta)}(s), v^{(\eta)}(s)\Big) - \Big(x(s), z(s)\Big)\right\| ds,\end{aligned}$$

where the last inequality follows from (S9). Using Gronwall's inequality we can conclude that with $C_1 = 2M$, we have

$$\limsup_{\eta\to\infty} \sup_{t\in[0,\mu_\eta(\epsilon)\wedge T]} \frac{\left\|\Big(x^{(\eta)}(t), v^{(\eta)}(t)\Big) - \Big(x(t), z(t)\Big)\right\|}{(\mu_\eta(\epsilon)\wedge T)e^{C_1\mu_\eta(\epsilon)\wedge T}} \leq C_1\epsilon, \tag{S10}$$

which proves (S7). Combining this with (S9) and $\left|w^{(\eta)}(s) - |z(s)|\right| \leq \left|w^{(\eta)}(s)\right| + \left|v^{(\eta)}(s) - z(s)\right|$ we can easily show (S8). This completes the proof of this proposition. □

Let the functions $\Psi_t, \Phi : \mathbb{R}_+^2 \to \mathbb{R}_+^2$ be as in Lemma S1 and Remark S1, respectively. We extend these functions to functions from $\mathbb{R}_+^2$ to $\mathbb{R}_+^{L+2}$ by defining

$$\widehat{\Psi}_t(x,a,b) \triangleq (x, \Psi_t(a,b)) \qquad \text{and} \qquad \widehat{\Phi}(x,a,b) \triangleq (x, \Phi(a,b)).$$

Recall that $X^{(\eta)}(t) = \left(x^{(\eta)}(t), z_1^{(\eta)}(t), z_2^{(\eta)}(t)\right)$ is a solution to the system $\mathcal{S}_\eta$, and define another process $Y^{(\eta)}(t)$ as

$$Y^{(\eta)}(t) \triangleq X^{(\eta)}(t) - \left[\widehat{\Psi}_{\eta t}\left(X^{(\eta)}(0)\right) - \widehat{\Phi}\left(X^{(\eta)}(0)\right)\right]. \tag{S11}$$

Note that $Y^{(\eta)}(t)$ has the form $Y^{(\eta)}(t) = \left(x^{(\eta)}(t), y_1^{(\eta)}(t), y_2^{(\eta)}(t)\right)$, where

$$\left(y_1^{(\eta)}(t), y_2^{(\eta)}(t)\right) = \left(z_1^{(\eta)}(t), z_2^{(\eta)}(t)\right) - \left[\Psi_{\eta t}\left(z_1^{(\eta)}(0), z_2^{(\eta)}(0)\right) - \Phi\left(z_1^{(\eta)}(0), z_2^{(\eta)}(0)\right)\right]. \tag{S12}$$

For time t bounded away from 0, $\widehat{\Psi}_{\eta t}\left(X^{(\eta)}(0)\right) - \widehat{\Phi}\left(X^{(\eta)}(0)\right)$ would be close to $(0,0,0)$; whereas for t near 0, $X^{(\eta)}(t) - \widehat{\Psi}_{\eta t}\left(X^{(\eta)}(0)\right)$ would be close to $(0,0,0)$. As it turns out, process $Y^{(\eta)}(t)$ has better convergence properties (as $\eta \to \infty$) in comparison to process $X^{(\eta)}(t)$. We shall use the results in Katzenberger [83], to prove the following proposition which will play a key role in the proof of Theorem 1.

Proposition S2 *Suppose that* $\lim_{\eta\to\infty}\left(x^{(\eta)}(0), z_1^{(\eta)}(0) - z_2^{(\eta)}(0)\right) = (x_0, \alpha)$ *for some* $(x_0,\alpha) \in \mathbb{R}_+^L \times \mathbb{R}$, *and let* $\Big(x(t), z(t)\Big)$ *be the solution of system* $\mathcal{S}$ *with initial condition* (x_0, α). *Fix a small* $\epsilon > 0$, *and define* $\sigma_\eta(\epsilon)$ *as*

$$\sigma_\eta(\epsilon) \triangleq \inf\left\{t \geq 0 : \left|z_1^{(\eta)}(t) - z_2^{(\eta)}(t)\right| \leq \epsilon\right\}. \tag{S13}$$

Then we have the following

(A) $\lim_{\eta\to\infty} \sigma_\eta(\epsilon) = \sigma(\epsilon) \triangleq \inf\{t \geq 0 : |z(t)| \leq \epsilon\}$.

(B) If $\sigma(\epsilon) \in (0,\infty)$ *then*

$$\Big(z^+(\sigma(\epsilon)), z^-(\sigma(\epsilon))\Big) = \begin{cases} (\epsilon, 0) & \text{if} \quad \alpha > 0 \\ (0, \epsilon) & \text{if} \quad \alpha < 0. \end{cases}$$

and for any $T > 0$

$$\limsup_{\eta\to\infty} \sup_{t\in[\delta,\sigma_\eta(\epsilon)\wedge T]} \left\| X^{(\eta)}(t) - \big(x(t), z^+(t), z^-(t)\big)\right\| = 0, \tag{S14}$$

where δ *is any positive number less that* $\sigma(\epsilon)$.

Proof. Without loss of generality we can assume that there is a $\theta \in \mathbb{R}_+$ such that $X^{(\eta)}(0) = (x_0, \theta + \alpha, \theta)$ for all η. Clearly the assertions of this proposition become trivial when $|\alpha| \leq \epsilon$, since $\sigma(\epsilon) = 0$, and so we can assume that $|\alpha| > \epsilon$.

We shall prove the proposition under the assumption $\alpha > \epsilon$. The other case $\alpha < -\epsilon$ follows by symmetry. We first consider the case $\theta > 0$. Define $\Gamma = \{(x, z, 0) \in \mathbb{R}_+^{L+2} : x \in \mathbb{R}_+^L \text{ and } z > 0\}$ and $U_\Gamma = \{(x, z_1, z_2) \in \mathbb{R}_+^{L+2} : x \in \mathbb{R}_+^L \text{ and } z_1 > z_2 \geq 0\}$. Observe that U_Γ is an open set in $\mathbb{R}_+^{L+2}$ containing Γ. Moreover Γ is an $(L-1)$-dimensional continuous manifold and the function $\widehat{\Phi}$ is continuously differentiable on Γ. In what follows, we denote the vector or matrix of zeros by $\mathbf{0}$. Letting $G(x, z_1, z_2) \triangleq (\mathbf{0}, -z_1 z_2, -z_1 z_2)$ and $W(x, z_1, z_2) \triangleq \Big(F(x, z_1, z_2), W_1(x, z_1, z_2), W_2(x, z_1, z_2)\Big)$, we can see that $X^{(\eta)}(t)$ satisfies

$$\dot{X}^{(\eta)} = W\left(X^{(\eta)}(t)\right) + \eta G\left(X^{(\eta)}(t)\right); \qquad X^{(\eta)}(0) = (x_0, \theta + \alpha, \theta).$$

Also at any $(x, z, 0) \in \Gamma$, the Jacobian matrix $\partial G(x, z, 0)$ of function G can be computed as

$$\partial G(x, z, 0) = z \begin{bmatrix} \mathbf{0} & \mathbf{0} & \mathbf{0} \\ \mathbf{0} & -1 & 0 \\ \mathbf{0} & -1 & 0 \end{bmatrix},$$

and it has exactly one negative eigenvalue. For any $t \in [0, \sigma_\eta(\epsilon)]$, we have $Y^{(\eta)}(t) \in U_\Gamma$ for large η due to (S4). Let $\widetilde{Y}^{(\eta)}(t)$ denote the trajectory of $Y^{(\eta)}(t)$ stopped at time $\sigma_\eta(\epsilon)$, i.e.

$$\widetilde{Y}^{(\eta)}(t) = Y^{(\eta)}(t \wedge \sigma_\eta(\epsilon)) \qquad \text{for} \qquad t \geq 0.$$

From Theorem 6.3 in [83] we can conclude that the sequence $(\widetilde{Y}^{(\eta)}, \sigma_\eta(\epsilon))$ converges in the Skorohod topology on $D_{\mathbb{R}_+^2}[0,\infty) \times [0,\infty]$ to $(\widetilde{Y}, \sigma(\epsilon))$, where

$$\sigma(\epsilon) = \inf\left\{t \geq 0 : |\widetilde{y}_1(t) - \widetilde{y}_2(t)| < \frac{\epsilon}{2}\right\}, \tag{S15}$$

and the trajectory $\widetilde{Y}(t) = \Big(\widetilde{x}(t), \widetilde{y}_1(t), \widetilde{y}_2(t)\Big)$ lies in the set Γ and satisfies

$$\widetilde{Y}(t) = \widetilde{Y}(0) + \int_0^{t\wedge\sigma(\epsilon)} \partial\widehat{\Phi}\Big(\widetilde{Y}(s)\Big) W\Big(\widetilde{Y}(s)\Big) ds. \tag{S16}$$

However $\widehat{\Phi}(x, z_1, z_2) = (x, z_1 - z_2, 0)$ on U_Γ, and hence the Jacobian matrix is simply

$$\partial\Phi(x, z, 0) = \begin{bmatrix} \mathbf{I} & \mathbf{0} & \mathbf{0} \\ \mathbf{0} & 1 & -1 \\ \mathbf{0} & 0 & 0 \end{bmatrix},$$

for any $(x, z, 0) \in \Gamma$, where $\mathbf{I}$ is the $L \times L$ identity matrix. Hence we can rewrite equation (S16) for $\widetilde{Y}(t) = \big(\widetilde{x}(t), \widetilde{y}_1(t), \widetilde{y}_2(t)\big)$ as

$$\begin{aligned} \widetilde{x}(t) &= \widetilde{x}(0) + \int_0^{t\wedge\sigma(\epsilon)} F\big(\widetilde{x}(s), \widetilde{y}_1(s), \widetilde{y}_2(s)\big) ds \\ \widetilde{y}_1(t) &= \widetilde{y}_1(0) + \int_0^{t\wedge\sigma(\epsilon)} \Big(W_1\big(\widetilde{x}(s), \widetilde{y}_1(s), \widetilde{y}_2(s)\big) - W_2\big(\widetilde{x}(s), \widetilde{y}_1(s), \widetilde{y}_2(s)\big)\Big) ds \\ \text{and} \quad \widetilde{y}_2(t) &= 0. \end{aligned}$$

Since $X^{(\eta)}(0) = (x_0, \theta + \alpha, \theta)$, we see that $\widetilde{Y}(0) = \big(\widetilde{x}(0), \widetilde{y}_1(0), \widetilde{y}_2(0)\big) = \widehat{\Phi}(x_0, \theta + \alpha, \theta) = (x_0, \alpha, 0)$. Hence $\widetilde{x}(0) = x_0$ and $\widetilde{y}_1(0) = \alpha$, and so we have $\big(\widetilde{x}(t), \widetilde{y}_1(t)\big) = \big(x(t), z(t)\big)$ in the time interval $[0, \sigma(\epsilon)]$, where $(x(t), z(t))$ is as in the statement of this proposition. As $\widetilde{y}_2(t) = 0$ until time $\sigma(\epsilon)$, we can express (S15) as

$$\sigma(\epsilon) = \inf\{t \geq 0 : 0 < z(t) < \epsilon\}. \tag{S17}$$

This proves part (A) of the proposition.

Suppose $\sigma(\epsilon) < \infty$ and since $\alpha > \epsilon$ we also have $\sigma(\epsilon) > 0$. Due to continuity of the trajectories $z(\sigma(\epsilon)) = \epsilon$. These facts along with the convergence $\Big(\widetilde{Y}^{(\eta)}, \sigma_\eta(\epsilon)\Big) \longrightarrow \Big(\widetilde{Y}, \sigma(\epsilon)\Big)$ in the Skorohod topology and (S4), imply that

$$\limsup_{\eta\to\infty} \sup_{t\in[\delta,\sigma_\eta(\epsilon)\wedge T]} \left\| X^{(\eta)}(t) - \big(x(t), z^+(t), z^-(t)\big) \right\| = \limsup_{\eta\to\infty} \sup_{t\in[\delta,\sigma_\eta(\epsilon)\wedge T]} \left\| Y^{(\eta)}(t) - \big(x(t), z^+(t), z^-(t)\big) \right\| = 0,$$

thereby proving part (B).

We now come to the case $\theta = z_2^{(\eta)}(0) = 0$. In this case we can set $Y^{(\eta)} = Z^{(\eta)}$ and use Theorem 6.2 in [83] to conclude the above convergence in the Skorohod topology. The rest of the arguments go through as before. This concludes the proof of this proposition. □

Lemma S3 *Fix a $T > 0$, $\epsilon_0 > 0$ and an $\epsilon \in (0, \epsilon_0)$. Suppose for some $(x_0, \alpha) \in \mathbb{R}_+^L \times \mathbb{R}$, the sequence of initial conditions $\Big\{X^{(\eta)}(0) = \Big(x^{(\eta)}(0), z_1^{(\eta)}(0), z_2^{(\eta)}(0)\Big)\Big\}$ satisfies*

$$\limsup_{\eta\to\infty} \left\| \Big(x^{(\eta)}(0), z_1^{(\eta)}(0) - z_2^{(\eta)}(0)\Big) - (x_0, \alpha) \right\| \leq \epsilon_0.$$

Define $\sigma_\eta(\epsilon)$ and $\mu_\eta(\epsilon)$ as

$$\sigma_\eta(\epsilon) \triangleq \inf\left\{t \geq 0 : \left|z_1^{(\eta)}(t) - z_2^{(\eta)}(t)\right| < \epsilon\right\} \quad \text{and} \quad \mu_\eta(\epsilon) \triangleq \inf\left\{t \geq \sigma_\eta(\epsilon) : \left|z_1^{(\eta)}(t) - z_2^{(\eta)}(t)\right| > 2\epsilon\right\}.$$

Letting $\big(x(t), z(t)\big)$ be the solution of system $\mathcal{S}$, we have the following

(A) There exists a constant $C_1 > 0$ such that

$$\limsup_{\eta\to\infty} \left[e^{-C_1(\mu_\eta(\epsilon)\wedge T)} \sup_{t\in[0,\mu_\eta(\epsilon)\wedge T]} \left\| \Big(x^{(\eta)}(t), z_1^{(\eta)}(t) - z_2^{(\eta)}(t)\Big) - \Big(x(t), z(t)\Big) \right\| \leq \epsilon_0 \right].$$

(B) There exists a constant $C_2 > 0$ such that

$$\limsup_{\eta\to\infty} \frac{1}{\mu_\eta(\epsilon)\wedge T} \int_0^{\mu_\eta(\epsilon)\wedge T} \left\| \Big(x^{(\eta)}(t), z_1^{(\eta)}(t), z_2^{(\eta)}(t)\Big) - \Big(x(t), z^+(t), z^-(t)\Big) \right\| dt \leq C_2\epsilon_0.$$

Moreover the constants C_1 and C_2 can be chosen to be independent of ϵ_0, ϵ and (x_0, α).

Proof. Without loss of generality we can assume that there exists a $\widetilde{x}_0 \in \mathbb{R}^L_+, \widetilde{\theta} > 0$ and $\widetilde{\alpha} \in \mathbb{R}$ such that $X^{(\eta)}(0) = (\widetilde{x}_0, \widetilde{\theta} + \widetilde{\alpha}, \widetilde{\theta})$ for each η and

$$\|(\widetilde{x}_0, \widetilde{\alpha}) - (x_0, \alpha)\| \leq \epsilon_0.$$

Let $\left(\widetilde{x}(t), \widetilde{z}(t)\right)$ be the solution of system $\mathcal{S}$ but with initial condition $(\widetilde{x}_0, \widetilde{\alpha})$. Due to the Lipchitz nature of functions F, W_1 and W_2 there exists a constant $M > 0$ such that for any $t \geq 0$

$$\left\| \left(\widetilde{x}(t), \widetilde{z}(t)\right) - \left(x(t), z(t)\right) \right\| \leq \epsilon_0 e^{Mt}. \tag{S18}$$

For convenience we assume that $\mu_\eta(\epsilon) \leq T$, but the proof works in the case $\mu_\eta(\epsilon) \geq T$ as well. Observe that

$$\begin{aligned}
&\sup_{t \in [0, \mu_\eta(\epsilon)]} \left\| \left(x^{(\eta)}(t), z_1^{(\eta)}(t) - z_2^{(\eta)}(t)\right) - \left(x(t), z(t)\right) \right\| \\
&\quad \leq \sup_{t \in [0, \mu_\eta(\epsilon)]} \left\| \left(x^{(\eta)}(t), z_1^{(\eta)}(t) - z_2^{(\eta)}(t)\right) - \left(\widetilde{x}(t), \widetilde{z}(t)\right) \right\| + \sup_{t \in [0, \mu_\eta(\epsilon)]} \left\| \left(\widetilde{x}(t), \widetilde{z}(t)\right) - \left(x\big(\mu_\eta(\epsilon)\big), z\big(\mu_\eta(\epsilon)\big)\right) \right\| \\
&\quad \leq \sup_{t \in [0, \mu_\eta(\epsilon)]} \left\| \left(x^{(\eta)}(t), z_1^{(\eta)}(t) - z_2^{(\eta)}(t)\right) - \left(\widetilde{x}(t), \widetilde{z}(t)\right) \right\| + \epsilon_0 e^{M \mu_\eta(\epsilon)},
\end{aligned} \tag{S19}$$

where the last inequality holds due to (S18). From Proposition S2 we know that

$$\limsup_{\eta \to \infty} \sup_{t \in [0, \mu_\eta(\epsilon)]} \left\| \left(x^{(\eta)}(t), z_1^{(\eta)}(t) - z_2^{(\eta)}(t)\right) - \left(\widetilde{x}(t), \widetilde{z}(t)\right) \right\| = 0,$$

and $\sigma_\eta(\epsilon) \to \sigma(\epsilon) \triangleq \inf\{t \geq 0 : |\widetilde{y}(t)| < \epsilon\}$. Without losing generality we can assume that $\sigma_\eta(\epsilon) = \sigma(\epsilon)$ and $\left(x^{(\eta)}(t), z_1^{(\eta)}(t) - z_2^{(\eta)}(t)\right) = \left(\widetilde{x}(t), \widetilde{z}(t)\right)$ for each η and $t \in [0, \sigma(\epsilon)]$. Let $\tau_\eta(\epsilon) \triangleq \mu_\eta(\epsilon) - \sigma(\epsilon)$, and for any $t \in [0, \tau_\eta(\epsilon)]$ define

$$\begin{aligned}
\left(\bar{x}^{(\eta)}(t), \bar{z}_1^{(\eta)}(t), \bar{z}_2^{(\eta)}(t)\right) &\triangleq \left(\widetilde{x}^{(\eta)}(t + \sigma(\epsilon)), \widetilde{z}_1^{(\eta)}(t + \sigma(\epsilon)), \widetilde{z}_2^{(\eta)}(t + \sigma(\epsilon))\right) \\
\left(\bar{x}(t), \bar{z}(t)\right) &\triangleq \left(\widetilde{x}(t + \sigma(\epsilon)), \widetilde{z}(t + \sigma(\epsilon))\right).
\end{aligned}$$

One can see that

$$\sup_{t \in [0, \mu_\eta(\epsilon)]} \left\| \left(x^{(\eta)}(t), z_1^{(\eta)}(t) - z_2^{(\eta)}(t)\right) - \left(\widetilde{x}(t), \widetilde{z}(t)\right) \right\| = \sup_{t \in [0, \tau_\eta(\epsilon)]} \left\| \left(\bar{x}^{(\eta)}(t), \bar{z}_1^{(\eta)}(t) - \bar{z}_2^{(\eta)}(t)\right) - \left(\bar{x}(t), \bar{z}(t)\right) \right\|.$$

Also note that $\lim_{\eta \to \infty} \left(\bar{x}^{(\eta)}(0), \bar{z}_1^{(\eta)}(0), \bar{z}_2^{(\eta)}(0)\right) = \left(\widetilde{x}(\sigma(\epsilon)), \epsilon, 0\right)$ or $(\widetilde{x}\left(\sigma(\epsilon)), 0, \epsilon\right)$. From Proposition S1, relation (S7) we see that there exists a constant $C > 0$ such that for large η we have

$$\sup_{t \in [0, \tau_\eta(\epsilon)]} \left\| \left(\bar{x}^{(\eta)}(t), \bar{z}_1^{(\eta)}(t) - \bar{z}_2^{(\eta)}(t)\right) - \left(\bar{x}(t), \bar{z}(t)\right) \right\| \leq C \epsilon \tau_\eta(\epsilon) e^{C \tau_\eta(\epsilon)}.$$

Using (S19) we obtain the following for large η:

$$\sup_{t \in [0, \mu_\eta(\epsilon)]} \left\| \left(x^{(\eta)}(t), z_1^{(\eta)}(t) - z_2^{(\eta)}(t)\right) - \left(x(t), z(t)\right) \right\| \leq C \epsilon \tau_\eta(\epsilon) e^{C \tau_\eta(\epsilon)} + \epsilon_0 e^{M \mu_\eta(\epsilon)}.$$

Let $\bar{M} \triangleq \max\{C, M\}$. Since $\epsilon \leq \epsilon_0, \tau_\eta(\epsilon) \leq \mu_\eta(\epsilon)$ and $1 + x \leq e^x$ for any $x \geq 0$, we get

$$\sup_{t \in [0, \mu_\eta(\epsilon)]} \left\| \left(x^{(\eta)}(t), z_1^{(\eta)}(t) - z_2^{(\eta)}(t)\right) - \left(x(t), z(t)\right) \right\| \leq \epsilon_0 e^{\bar{M} \mu_\eta(\epsilon)} \left(1 + \bar{M} \mu_\eta(\epsilon)\right) \leq \epsilon_0 e^{2 \bar{M} \mu_\eta(\epsilon)},$$

which proves part (A) of the proposition. The proof of part (B) is similar except that supremum must be replaced by time-integral, and instead of relation (S7) we use relation (S8) in Proposition S1. □

We now have all the tools to prove the main result of this section.

Proof.[Proof of Theorem S1] Let $X^{(\eta)}(t) = \left(x^{(\eta)}(t), z_1^{(\eta)}(t), z_2^{(\eta)}(t)\right)$ and $\left(x(t), z(t)\right)$ be as in the statement of this theorem. Without loss of generality we can assume that $X^{(\eta)}(0) = (x_0, a, b)$ for each η. We first consider the case $a \neq b$. Pick an $\epsilon > 0$. Let $\mu_\eta^{(0)}(\epsilon) = 0$ and for each $i = 1, 2, \dots$ define $\sigma_\eta^{(i)}(\epsilon)$ and $\mu_\eta^{(i)}(\epsilon)$ as

$$\sigma_\eta^{(i)}(\epsilon) \triangleq \inf\left\{t \geq \mu_\eta^{(i-1)}(\epsilon) : \left|z_1^{(\eta)}(t) - z_2^{(\eta)}(t)\right| < \epsilon\right\} \text{ and } \mu_\eta^{(i)}(\epsilon) \triangleq \inf\left\{t \geq \sigma_\eta^{(i)}(\epsilon) : \left|z_1^{(\eta)}(t) - z_2^{(\eta)}(t)\right| > 2\epsilon\right\}.$$

Let constants C_1 and C_2 be as in Lemma S3. Let $\epsilon_0 = \epsilon$ and for $i = 1, 2, \dots$ define

$$\epsilon_i \triangleq \epsilon_{i-1} e^{C_1\left(\mu_\eta^{(i)}(\epsilon)\wedge T - \mu_\eta^{(i-1)}(\epsilon)\wedge T\right)} = \epsilon e^{\sum_{j=1}^{i} C_1\left(\mu_\eta^{(j)}(\epsilon)\wedge T - \mu_\eta^{(j-1)}(\epsilon)\wedge T\right)} = \epsilon e^{C_1\left(\mu_\eta^{(i)}(\epsilon)\wedge T\right)}.$$

Using Proposition S1 for $i = 1$ and Lemma S3 part (A) for $i \geq 2$ we get that for each i we have

$$\sup_{t\in\left[0, \mu_\eta^{(i)}(\epsilon)\wedge T\right]} \left\|\left(x^{(\eta)}(t), z_1^{(\eta)}(t) - z_2^{(\eta)}(t)\right) - \left(x(t), z(t)\right)\right\| \leq \epsilon_i,$$

for large η. Note that $\epsilon_i < \epsilon e^{C_1 T}$. Letting $\eta \to \infty$ and then $i \to \infty$, and using Monotone Convergence Theorem implies that

$$\limsup_{\eta\to\infty} \sup_{t\in[0,T]} \left\|\left(x^{(\eta)}(t), z_1^{(\eta)}(t) - z_2^{(\eta)}(t)\right) - \left(x(t), z(t)\right)\right\| \leq \epsilon e^{C_1 T},$$

and letting $\epsilon \to 0$ proves the first convergence in Theorem S1.

From part (B) of Lemma S3 we know that for each $i = 1, 2, \dots$ we have

$$\int_{\mu_\eta^{(i-1)}(\epsilon)\wedge T}^{\mu_\eta^{(i)}(\epsilon)\wedge T} \left\|\left(x^{(\eta)}(t), z_1^{(\eta)}(t), z_2^{(\eta)}(t)\right) - \left(x(t), z^+(t), z^-(t)\right)\right\| dt \leq C_2\epsilon_{i-1}\left(\mu_\eta^{(i)}(\epsilon)\wedge T - \mu_\eta^{(i-1)}(\epsilon)\wedge T\right),$$

for large η. This shows that

$$\begin{aligned}
&\int_0^T \left\|\left(x^{(\eta)}(t), z_1^{(\eta)}(t), z_2^{(\eta)}(t)\right) - \left(x(t), z^+(t), z^-(t)\right)\right\| dt \\
&\quad = \sum_{i=1}^{\infty} \int_{\mu_\eta^{(i-1)}(\epsilon)\wedge T}^{\mu_\eta^{(i)}(\epsilon)\wedge T} \left\|\left(x^{(\eta)}(t), z_1^{(\eta)}(t), z_2^{(\eta)}(t)\right) - \left(x(t), z^+(t), z^-(t)\right)\right\| dt \\
&\quad \leq \sum_{i=1}^{\infty} C_2\epsilon_{i-1}\left(\mu_\eta^{(i)}(\epsilon)\wedge T - \mu_\eta^{(i-1)}(\epsilon)\wedge T\right) \\
&\quad \leq \epsilon C_2 e^{C_1 T} \sum_{i=1}^{\infty}\left(\mu_\eta^{(i)}(\epsilon)\wedge T - \mu_\eta^{(i-1)}(\epsilon)\wedge T\right) \leq \epsilon C_2 e^{C_1 T} T.
\end{aligned}$$

Letting $\eta \to \infty$ and then $\epsilon \to 0$ proves the second convergence in Theorem S1. This completes the proof. □

We now prove Theorem 2 in the main text, which we state again here as Theorem S2.

Theorem S2 *Under Assumption 3 and as $\eta \to \infty$, the limit point $(\bar{x}, \bar{z}_1, \bar{z}_2)$ of any convergent sub-sequence of non-negative fixed points of system $\mathcal{S}_\eta$ can be transformed to a fixed point of system $\mathcal{S}$ given by $(\bar{x}, \bar{z}_1 - \bar{z}_2)$.*

Proof. Let $\bar{X}^{(\eta)} \triangleq \left(\bar{x}^{(\eta)}, \bar{z}_1^{(\eta)}, \bar{z}_2^{(\eta)}\right)$ denote a non-negative fixed point of system $\mathcal{S}_\eta$, with $\eta > 0$. Hence, we have

$$\begin{cases} F\left(\bar{X}^{(\eta)}\right) = 0 \\ \dfrac{1}{\eta}W_1\left(\bar{X}^{(\eta)}\right) - \bar{z}_1^{(\eta)}\bar{z}_2^{(\eta)} = 0 \\ \dfrac{1}{\eta}W_2\left(\bar{X}^{(\eta)}\right) - \bar{z}_1^{(\eta)}\bar{z}_2^{(\eta)} = 0 \end{cases} \iff \begin{cases} F\left(\bar{X}^{(\eta)}\right) = 0 \\ W_1\left(\bar{X}^{(\eta)}\right) - W_2\left(\bar{X}^{(\eta)}\right) = 0 \\ \dfrac{1}{\eta}W_2\left(\bar{X}^{(\eta)}\right) - \bar{z}_1^{(\eta)}\bar{z}_2^{(\eta)} = 0. \end{cases} \tag{S20}$$

Since the limit of $\bar{X}^{(\eta)}$ as $\eta \to \infty$ is not always assumed to exist, then we only consider convergent sub-sequences with limiting points. More precisely, for each $i = 1, 2, \cdots$, let $\left\{\eta_k^i\right\}_{k\in\mathbb{N}}$ be a sequence with $\eta_k^i > 0$ and $\lim_{k\to\infty} \eta_k^i = \infty$ such that $\bar{X}^i = \left(\bar{x}^i, \bar{z}_1^i, \bar{z}_2^i\right) \triangleq \lim_{k\to\infty} \bar{X}^{(\eta_k^i)}$ is a limiting fixed point along the convergent sub-sequence $\left\{\bar{X}^{(\eta_k^i)}\right\}_{k\in\mathbb{N}}$. Taking the limit of (S20) along said sub-sequence yields

$$\begin{cases} \lim_{k\to\infty} F\left(\bar{X}^{(\eta_k^i)}\right) = 0 \\ \lim_{k\to\infty} W_1\left(\bar{X}^{(\eta_k^i)}\right) - \lim_{k\to\infty} W_2\left(\bar{X}^{(\eta_k^i)}\right) = 0 \\ \lim_{k\to\infty} \frac{1}{\eta_k^i} W_2\left(\bar{X}^{(\eta_k^i)}\right) - \lim_{k\to\infty} \bar{z}_1^{(\eta_k^i)} \bar{z}_2^{(\eta_k^i)} = 0 \end{cases} \implies \begin{cases} F\left(\bar{X}^i\right) = 0 \\ W_1\left(\bar{X}^i\right) - W_2\left(\bar{X}^i\right) = 0 \\ \bar{z}_1^i \bar{z}_2^i = 0, \end{cases} \tag{S21}$$

which follows from the Lipschitz assumption on F, W_1 and W_2 that allows us to move the limits inside the functions and exploit the fact that $W_j\left(\bar{X}^{(\eta_k^i)}\right)$ for $j = 1, 2$ remain finite in the limit as $k \to \infty$. Now define $\bar{z}^i \triangleq \bar{z}_1^i - \bar{z}_2^i$. Then, invoking the non-negativity assumption of the fixed points, we have

$$\begin{cases} \bar{z}_1^i \bar{z}_2^i = 0 \\ \bar{z}_1^i, \bar{z}_2^i \geq 0 \end{cases} \implies \begin{cases} \bar{z}_1^i = \max(\bar{z}^i, 0) = \left(\bar{z}^i\right)^+ \\ \bar{z}_2^i = \max(-\bar{z}^i, 0) = \left(\bar{z}^i\right)^- . \end{cases} \tag{S22}$$

Substituting in (S21) yields

$$\begin{cases} F\left(\bar{x}^i, \left(\bar{z}^i\right)^+, \left(\bar{z}^i\right)^-\right) = 0 \\ W_1\left(\bar{x}^i, \left(\bar{z}^i\right)^+, \left(\bar{z}^i\right)^-\right) - W_2\left(\bar{x}^i, \left(\bar{z}^i\right)^+, \left(\bar{z}^i\right)^-\right) = 0, \end{cases} \tag{S23}$$

which are exactly the equations of the fixed points for system $\mathcal{S}$ and thus completing the proof. □

We now prove Corollary 1 in the main text, which we state again here as Corollary S1.

Corollary S1 *Under Assumption S1, if system $\mathcal{S}$ admits a unique fixed point $(\bar{x}, \bar{z})$, then all convergent sub-sequences of non-negative fixed points of system $\mathcal{S}_\eta$ have a single limit point as $\eta \to \infty$ given by $(\bar{x}, \bar{z}^+, \bar{z}^-)$. However, if system $\mathcal{S}$ admits no fixed points, then there is no convergent sub-sequence of non-negative fixed points of system $\mathcal{S}_\eta$.*

Proof. Theorem S2 establishes that all convergent sub-sequences, as $\eta \to \infty$, of non-negative fixed points of system $\mathcal{S}_\eta$ yield a subset of the fixed points of system $\mathcal{S}$. Hence, if there is only one fixed point for system $\mathcal{S}$, then there exists only one such convergent sub-sequence. The second part of the corollary is the contrapositive of Theorem S2. □

S2 The Model Reduction result for sequestration-based CRNs (stochastic setting)

We now consider the same model reduction problem in the case where the dynamics are given by a continuous-time Markov chain over the non-negative integer lattice. Suppose we have a well-stirred system consisting of L species. The state at any time can be described by a vector $x \in \mathbb{N}_0^L$. There are K reaction channels, and when the state is x the k-th reaction channel fires at rate $\lambda_k(x)$ and displaces the state to $(x + \zeta_k)$ for some $\zeta_k \in \mathbb{Z}^L$. We assume that for any $k = 1, \ldots, K$ and state $x \in \mathbb{N}_+^L$, if $\lambda_k(x) > 0$ then $(x + \zeta_k) \in \mathbb{N}_0^L$. This ensures that the trajectories of the CTMC lie in the non-negative integer orthant $\mathbb{N}_0^L$. We now augment the system with two more species $\mathbf{Z}_1$ and $\mathbf{Z}_2$ and the following five additional reactions:

Reaction	Propensity	Stoichiometry Vector
$\varnothing \longrightarrow \mathbf{Z}_1$	$W_{1+}(x, z_1, z_2)$	$(\mathbf{0}, 1, 0)$
$\mathbf{Z}_1 \longrightarrow \varnothing$	$W_{1-}(x, z_1, z_2)$	$(\mathbf{0}, -1, 0)$
$\varnothing \longrightarrow \mathbf{Z}_2$	$W_{2+}(x, z_1, z_2)$	$(\mathbf{0}, 0, 1)$
$\mathbf{Z}_2 \longrightarrow \varnothing$	$W_{2-}(x, z_1, z_2)$	$(\mathbf{0}, 0, -1)$
$\mathbf{Z}_1 + \mathbf{Z}_2 \longrightarrow \varnothing$	$\eta z_1 z_2$	$(\mathbf{0}, -1, -1)$

Observe that the functions W_j, for $j = 1, 2$ from (S1) are now decomposed into two non-negative components $W \triangleq W_{j+} - W_{j-}$ such that the dynamics remain in the non-negative orthant. We also suppose that all the propensity functions λ_k, for $k = 1, \cdots, K$ can depend on the copy-numbers of these two species $\mathbf{Z}_1$ and $\mathbf{Z}_2$. The random time-change representation for the overall dynamics is given by

$$
\begin{aligned}
X^{(\eta)}(t) &= X^{(\eta)}(0) + \sum_{k=1}^{K} Y_k \left(\int_0^t \lambda_k \left(X^{(\eta)}(s), Z_1^{(\eta)}(s), W_2^{(\eta)}(s) \right) ds \right) \zeta_k \\
Z_1^{(\eta)}(t) &= Z_1^{(\eta)}(0) + \sum_{\ell \in \{+,-\}} \ell Y_{K+1}^{\ell} \left(\int_0^t W_{1l} \left(X^{(\eta)}(s), Z_1^{(\eta)}(s), Z_2^{(\eta)}(s) \right) ds \right) - Y_{K+3} \left(\eta \int_0^t Z_1^{(\eta)}(s) Z_2^{(\eta)}(s) ds \right) \\
Z_2^{(\eta)}(t) &= Z_2^{(\eta)}(0) + \sum_{\ell \in \{+,-\}} \ell Y_{K+2}^{\ell} \left(\int_0^t W_{2\ell} \left(X^{(\eta)}(s), Z_1^{(\eta)}(s), Z_2^{(\eta)}(s) \right) ds \right) - Y_{K+3} \left(\eta \int_0^t Z_1^{(\eta)}(s) Z_2^{(\eta)}(s) ds \right),
\end{aligned}
$$

where $Y_1, \cdots, Y_K, Y_{K+1}^{\pm}, Y_{K+2}^{\pm}$ and Y_{K+3} are $K + 5$ independent unit-rate Poisson processes.

For large values of η, the reaction $\mathbf{Z}_1 + \mathbf{Z}_2 \longrightarrow \varnothing$ operates on a *faster* timescale than other reactions. We shall use the results in [84] to analyze the limiting behavior of the overall dynamics $\left(X^{(\eta)}(t), Z_1^{(\eta)}(t), Z_2^{(\eta)}(t) \right)_{t \geq 0}$ as $\eta \to \infty$. Let $Z^{(\eta)}(t) \triangleq Z_1^{(\eta)}(t) - Z_2^{(\eta)}(t)$ and note that the dynamics of the process $\left(X^{(\eta)}(t), Z^{(\eta)}(t) \right)_{t \geq 0}$ is unaffected by the fast reaction. Next, define a random measure on $\mathbb{N}_0^2 \times [0, \infty)$ by

$$
V^{(\eta)}(C \times [0, t]) = \int_0^t \mathbb{1}_C \left(Z_1^{(\eta)}(s), Z_2^{(\eta)}(s) \right) ds.
$$

For any $z \in \mathbb{Z}$, define an operator that acts on some bounded function f as

$$
\mathbb{C}^z f(z_1, z_2) = z_1 z_2 \Big(f(z_1 - 1, z_2 - 1) - f(z_1, z_2) \Big).
$$

This operator is the generator of the dynamics of species $\mathbf{Z}_1$ and $\mathbf{Z}_2$ due to the fast reaction $\mathbf{Z}_1 + \mathbf{Z}_2 \longrightarrow \varnothing$, when initial difference in their copy numbers is z. Note that the Markovian dynamics generated by this dynamics are ergodic with unique stationary distribution given by

$$
\pi_z(z_1, z_2) = \begin{cases} 1 & \text{if } z_1 = z, z_2 = 0 \text{ and } z \geq 0 \\ 1 & \text{if } z_1 = 0, z_2 = -z \text{ and } z < 0 \\ 0 & \text{otherwise.} \end{cases}
$$

For each $k = 1, \ldots, K$ and $j = 1, 2$ define

$$
\begin{aligned}
\widehat{\lambda}_k(x, z) &\triangleq \mathbb{E}_{\pi_z} \left[\lambda_k(x, Z_1, Z_2) \right] = \sum_{\mathbb{N}_0^2} \lambda_k(x, z_1, z_2) \pi_z(z_1, z_2) = \lambda_k \left(x, z^+, z^- \right) \\
\widehat{W}_{j\pm}(x, z) &\triangleq \mathbb{E}_{\pi_z} \left[W_{j\pm}(x, Z_1, Z_2) \right] = \sum_{\mathbb{N}_0^2} W_{j\pm}(x, z_1, z_2) \pi_z(z_1, z_2) = W_{j\pm} \left(x, z^+, z^- \right).
\end{aligned}
$$

As the Markov process corresponding to generator $\mathbb{C}^z$ is ergodic with stationary distribution π_z, we can expect that if we have $\left(X^{(\eta)}, Z^{(\eta)}, V^{(\eta)} \right) \to (X, Z, V)$ as $\eta \to \infty$, then the limiting occupation measure V has the form

$$
V(dy \times ds) = \pi_{Z(s)}(dy) ds. \tag{S24}
$$

Moreover since for each λ_k and $W_{j\pm}$ we have

$$
\int_0^t \lambda_k \left(X^{(\eta)}(s), Z_1^{(\eta)}(s), Z_2^{(\eta)}(s) \right) ds = \int_0^t \sum_{\mathbb{N}_0^2} \lambda_k \left(X^{(\eta)}(s), z_1, z_2 \right) V^{(\eta)} \Big(\{z_1, z_2\} \times ds \Big) \underset{\eta \to \infty}{\longrightarrow} \int_0^t \widehat{\lambda}_k \Big(X(s), Z(s) \Big) ds,
$$

and

$$
\int_0^t W_{j\pm} \left(X^{(\eta)}(s), Z_1^{(\eta)}(s), Z_2^{(\eta)}(s) \right) ds = \int_0^t \sum_{\mathbb{N}_0^2} W_{j\pm} \left(X^{(\eta)}(s), z_1, z_2 \right) V^{(\eta)} \Big(\{z_1, z_2\} \times ds \Big) \underset{\eta \to \infty}{\longrightarrow} \int_0^t \widehat{W}_{j\pm} \Big(X(s), Z(s) \Big) ds,
$$

the limiting process $(X(t), Z(t))_{t\geq 0}$ has the random time-change representation given by

$$X(t) = X(0) + \sum_{k=1}^{K} Y_k \left(\int_0^t \widehat{\lambda}_k \Big(X(s), Z(s) \Big) ds \right) \zeta_k \tag{S25}$$
$$\text{and} \quad Z(t) = Z(0) + \sum_{\ell \in \{+,-\}} \ell Y_{K+1}^{\ell} \left(\int_0^t \widehat{W}_{1\ell} \Big(X(s), Z(s) \Big) ds \right) - \sum_{\ell \in \{+,-\}} \ell Y_{K+2}^{\ell} \left(\int_0^t \widehat{W}_{2\ell} \Big(X(s), Z(s) \Big) ds \right).$$

We now state our main model reduction result in the stochastic setting, whose proof follows from Theorem 5.1 in [84]. A numerical validation of this result in presented in Figure S8.

Proposition S3 *Let the process* $\big(X^{(\eta)}, Z^{(\eta)}\big)_{t\geq 0}$ *and the occupation measure* $V^{(\eta)}$ *be as defined above. Suppose* $\big(X^{(\eta)}(0), Z^{(\eta)}(0)\big) \longrightarrow \big(X(0), U(0)\big)$ *as* $\eta \to \infty$*. We then have* $\big(X^{(\eta)}, Z^{(\eta)}, V^{(\eta)}\big) \longrightarrow (X, Z, V)$ *where* V *is given by* (S24) *and the process* $\big(X(t), Z(t)\big)_{t\geq 0}$ *is given by* (S25).

For a function $f : \mathbb{R}_+^L \times \mathbb{R} \to \mathbb{R}$ the convergence $\big(X^{(\eta)}(t), Z^{(\eta)}(t)\big)_{t\geq 0} \underset{\eta\to\infty}{\longrightarrow} \big(X(t), Z(t)\big)_{t\geq 0}$ implies that

$$\lim_{\eta\to\infty} \sup_{t\in[0,T]} \left| \mathbb{E}\left[f\left(X^{(\eta)}(t), Z_1^{(\eta)}(t) - Z_2^{(\eta)}(t) \right) \right] - \mathbb{E}\Big[f\Big(X(t), Z(t) \Big) \Big] \right| = 0.$$

for any $T > 0$. Since we also have convergence of the occupation measure we can also conclude that for any $h : \mathbb{R}_+^{L+2} \to \mathbb{R}$ we have

$$\lim_{\eta\to\infty} \int_0^T \left| \mathbb{E}\left[h\left(X^{(\eta)}(t), Z_1^{(\eta)}(t), Z_2^{(\eta)}(t) \right) \right] - \mathbb{E}\Big[h\Big(X(t), Z^+(t), Z^-(t) \Big) \Big] \right| dt = 0$$

for any $T > 0$.

S3 Proof of a couple of key Lemmas

S3.A Proof of Lemma 1

We first show the sufficiency of these conditions and then their necessity.

Sufficiency. Let $r \in [y_{\min}, y_{\max}]$, then the last two equations in (13) imply that $\bar{w} = \bar{y} = r$. If $r \in \mathcal{R}$ and the actuator does not saturate, then the supporting input exists and is given by $\bar{u} = \bar{\mathcal{P}}_{\bar{\Delta}}^{-1}(r) \in [u_{\min}, u_{\max}]$. This implies that $\bar{v}$ exists and is given by $\bar{v} = \bar{u} = \bar{\mathcal{P}}_{\bar{\Delta}}^{-1}(r)$.

Necessity. This can be straightforwardly established using the contra-positive, that is, if any of the conditions are not satisfied, then the fixed point does not exist with a feasible supporting input. In fact, if $r \notin \mathcal{R}$ then $\bar{u} \notin \mathbb{U}$. Otherwise, if $\bar{\mathcal{P}}_{\bar{\Delta}}^{-1}(r) \notin [u_{\min}, u_{\max}]$ and/or $r \notin [y_{\min}, y_{\max}]$, then it can be immediately seen from the second and third equations of (13) that $\bar{v}$ and/or $\bar{y}$ do not exist, respectively.

S3.B Proof of Lemma 2

The closed-loop dynamics describing the block diagram of Fig. 5(b) are given by the following set of differential equations.

$$\begin{array}{rll} \textbf{Controlled Process} & y = \mathcal{P}_\Delta(u) \quad \text{e.g.} & \begin{cases} \dot{x} = f_\Delta(x,u) \\ y = g_\Delta(x,u) \end{cases} \\ \textbf{Error} & e = r_{\text{in}} - w & \\ \textbf{Integrator} & \dot{v} = K_I e & \\ \textbf{Actuator} & u = \psi_a(v) & \\ \textbf{Sensor} & w = \psi_s(y). & \end{array} \tag{S26}$$

Thus the dynamics and the steady-state values, if they exist, satisfy

$$\begin{cases} y = \mathcal{P}_\Delta\big(\psi_a(v)\big) \\ \dot{v} = K_I\big(r_{\text{in}} - \psi_s(y)\big) \end{cases} \implies \begin{cases} \bar{\mathcal{P}}_{\bar{\Delta}}\big(\psi_a(\bar{v})\big) = \bar{y} \\ \psi_s(\bar{y}) = r_{\text{in}}. \end{cases}$$

Sufficiency. Let $r_{\text{in}} \in \text{range}(\psi_s)$, then there exists a $\bar{y} = r_{\text{out}}$ such that $\psi_s(r_{\text{out}}) = r_{\text{in}}$, and in fact it is unique since ψ_s is strictly monotonically increasing. To this end, we can write $\bar{y} = r_{\text{out}} = \psi_s^{-1}(r_{\text{in}})$. Let $r_{\text{out}} \in \mathcal{R}_{\text{out}}$, then the supporting input exists and is given by $\bar{u} = \bar{\mathcal{P}}_{\bar{\Delta}}^{-1}(r_{\text{out}}) = \bar{\mathcal{P}}_{\bar{\Delta}}^{-1} \circ \psi_s^{-1}(r_{\text{in}})$. Furthermore, if the actuator does not saturate, then $\bar{u} \in \text{range}(\psi_a)$ which implies that there exists a $\bar{v}$ such that $\psi_a(\bar{v}) = \bar{u}$. It is in fact unique since ψ_a is strictly monotonically increasing, and finally we can write $\bar{v} = \psi_a^{-1} \circ \bar{\mathcal{P}}_{\bar{\Delta}}^{-1} \circ \psi_s^{-1}(r_{\text{in}})$.

Necessity. This can be straightforwardly established using the contra-positive, that is, if any of the conditions are not satisfied, then the fixed point does not exist with a feasible supporting input. In fact, if $r_{\text{out}} \notin \mathcal{R}_{\text{out}}$ then $\bar{u} \notin \mathbb{U}$. Otherwise, if $\bar{\mathcal{P}}_{\bar{\Delta}}^{-1}(r_{\text{out}}) \notin \text{range}(\psi_a)$ and/or $r_{\text{in}} \notin \text{range}(\psi_s)$, then $\bar{v}$ and/or $\bar{y}$ do not exist, respectively.

S4 Anti-Windup Block Diagrams

Consider the closed-loop network depicted in Fig. 6(a) where the controller is given by either Topology I, II or III. We treat each topology separately.

Topology I. The controller dynamics can be expressed as

$$\begin{cases} \dot{z}_1 = \mu - \eta z_1 z_2 + h_1(z_2) \\ \dot{z}_2 = \theta h_s(y) - \eta z_1 z_2 + h_2(z_1) \\ u = k h_a(z_1). \end{cases} \tag{S27}$$

By invoking Theorem 1 as $\eta \to \infty$ (with $z \triangleq z_1 - z_2, W_1(x, z_1, z_2) \triangleq \mu + h_1(z_2)$ and $W_2(x, z_1, z_2) \triangleq \theta h_s(x_L) + h_2(z_1)$), we obtain the following reduced controller dynamics

$$\begin{cases} \dot{z} = \mu - \theta h_s(y) - \phi(z) \\ u = k h_a(z^+), \end{cases} \quad \text{with} \quad \begin{cases} z^+ \triangleq \max(z, 0) \\ z^- \triangleq \max(-z, 0) \\ \phi(z) \triangleq h_2(z^+) - h_1(z^-). \end{cases} \tag{S28}$$

If the functions h_1 and h_2 are given by

$$\begin{cases} h_1(z_2) = \alpha_1 \max(z_2 - \beta_1, 0) \\ h_2(z_1) = \alpha_2 \max(z_1 - \beta_2, 0), \end{cases} \tag{S29}$$

then $\phi(z)$ can be expressed as

$$\phi(z) = \begin{cases} \alpha_1(z + \beta_1) & \text{for} \quad z \leq -\beta_1 \\ 0 & \text{for} \quad -\beta_1 \leq z \leq \beta_2 \\ \alpha_2(z - \beta_2) & \text{for} \quad z \geq \beta_2, \end{cases} \tag{S30}$$

and is plotted in Fig. S1(a). Note that one can think of the function ϕ as a “forgetting function” because it causes the integral controller to “forget” the past error signals. Introducing the intermediate variables $v \triangleq kz, w \triangleq h_s(y)$ and $e \triangleq r_{\text{in}} - w$ and the integral gain $K_I \triangleq k\theta$ yields

$$\begin{aligned} &\dot{v} = K_I e - \psi(v), \quad \text{where} \quad \psi(v) = k\phi\left(\frac{v}{k}\right) \\ \implies &\psi(v) = \begin{cases} \alpha_1(v + k\beta_1) & \text{for} \quad v \leq -k\beta_1 \\ 0 & \text{for} \quad -k\beta_1 \leq v \leq k\beta_2 \\ \alpha_2(v - k\beta_2) & \text{for} \quad v \geq k\beta_2. \end{cases} \end{aligned} \tag{S31}$$

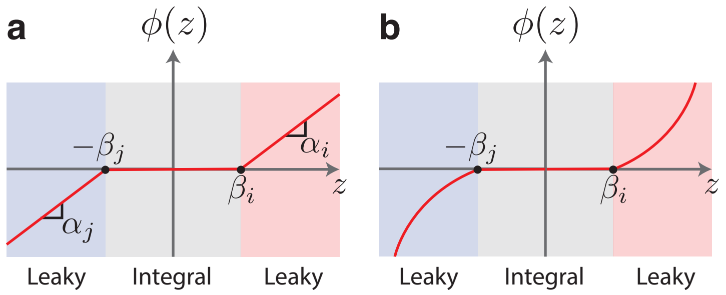

Figure S1: Forgetting functions $\phi(z)$. (a) Plot of $\phi(z)$ given in (S30). (b) Plot of $\phi(z)$ given in (S36).

The differential equations for v can thus be rewritten as

$$\begin{cases} \dot{v} = K_I e_1 - \alpha_1 v & \text{for} \quad v \le -k\beta_1 \\ \dot{v} = K_I e & \text{for} \quad -k\beta_1 \le v \le k\beta_2 \\ \dot{v} = K_I e_2 - \alpha_2 v & \text{for} \quad v \ge k\beta_2, \end{cases}$$
$$\text{where} \begin{cases} e_1 \triangleq e - \dfrac{\alpha_1\beta_1}{\theta} \\ e_2 \triangleq e + \dfrac{\alpha_2\beta_2}{\theta}. \end{cases} \tag{S32}$$

Taking the Laplace transforms to calculate the transfer function from the error e to v directly gives us the block diagram depicted in Fig. 6(b) with $(i, j) = (1, 2)$.

Topology II. The controller dynamics can be expressed as

$$\begin{cases} \dot{z}_1 = \mu - \eta z_1 z_2 - h_1(z_1) \\ \dot{z}_2 = \theta h_s(y) - \eta z_1 z_2 - h_2(z_2) \\ u = k h_a(z_1). \end{cases} \tag{S33}$$

By invoking Theorem 1 as $\eta \to \infty$ (with $z \triangleq z_1 - z_2, W_1(x, z_1, z_2) \triangleq \mu - h_1(z_1)$ and $W_2(x, z_1, z_2) \triangleq \theta h_s(x_L) - h_2(z_2)$), we obtain the following reduced controller dynamics

$$\begin{cases} \dot{z} = \mu - \theta h_s(y) - \phi(z) \\ u = k h_a(z^+), \end{cases} \quad \text{with} \quad \begin{cases} z^+ \triangleq \max(z, 0) \\ z^- \triangleq \max(-z, 0) \\ \phi(z) \triangleq h_1(z^+) - h_2(z^-). \end{cases} \tag{S34}$$

If the functions h_1 and h_2 are also given by (S29), then repeating the procedure applied for Topology I will yield an identical block diagram as depicted in Fig. 6(b), but with $(i, j) = (2, 1)$. However, if the functions h_1 and h_2 are given by

$$\begin{cases} h_1(z_1) = \alpha_1 \max(z_1 - \beta_1, 0) z_1 \\ h_2(z_2) = \alpha_2 \max(z_2 - \beta_2, 0) z_2, \end{cases} \tag{S35}$$

then $\phi(z)$ can be expressed as

$$\phi(z) = \begin{cases} \alpha_1 z(z - \beta_1) & \text{for} \quad z \ge \beta_1 \\ 0 & \text{for} \quad -\beta_2 \le z \le \beta_1 \\ -\alpha_2 z(z + \beta_2) & \text{for} \quad z \le \beta_2, \end{cases} \tag{S36}$$

and is plotted in Fig. S1(b). In this case, we do not draw the block diagram because the differential equations for v are not piece-wise linear anymore due to the quadratic terms present in $\phi(z)$. However, the underlying control-theoretic concept is very similar to that depicted in the block diagram of Fig. 6(b).

Topology III. The controller dynamics can be expressed as

$$\begin{cases} \dot{z}_1 = \mu h_1(z_1) - \eta z_1 z_2 \\ \dot{z}_2 = \theta h_s(y) h_2(z_2) - \eta z_1 z_2 \\ u = k h_a(z_1). \end{cases} \tag{S37}$$

By invoking Theorem 1 as $\eta \to \infty$ (with $z \triangleq z_1 - z_2, W_1(x, z_1, z_2) \triangleq \mu h_1(z_1)$ and $W_2(x, z_1, z_2) \triangleq \theta h_s(x_L) h_2(z_2)$), we obtain the following reduced controller dynamics

$$\begin{cases} \dot{z} = \mu h_1(z^+) - \theta h_s(y) h_2(z^-) \\ u = k h_a(z^+), \end{cases} \text{with} \begin{cases} z^+ \triangleq \max(z, 0) \\ z^- \triangleq \max(-z, 0). \end{cases} \tag{S38}$$

Introducing the intermediate variables $v \triangleq kz, w \triangleq h_s(y)$ and the integral gain $K_I \triangleq k\theta$ yields

$$\dot{v} = K_I \left[r_{\text{in}} h_1 \left(\max \left(\frac{v}{k}, 0 \right) \right) - w h_2 \left(\max \left(-\frac{v}{k}, 0 \right) \right) \right]. \tag{S39}$$

If the functions h_1 and h_2 are given by

$$\begin{cases} h_1(z_1) = \dfrac{1}{1 + \frac{1}{\alpha_1} \max(z_1 - \beta_1, 0)} \\ h_2(z_2) = \dfrac{1}{1 + \frac{1}{\alpha_2} \max(z_2 - \beta_2, 0)}, \end{cases} \tag{S40}$$

then the differential equations for v can be written as

$$\begin{cases} \dot{v} = K_I \left(r_{\text{in}} - \dfrac{k\alpha_2}{k(\alpha_2 - \beta_2) - v} w \right) & \text{for} \quad v \leq -k\beta_2 \\ \dot{v} = K_I (r_{\text{in}} - w) & \text{for} \quad -k\beta_2 \leq v \leq k\beta_1 \\ \dot{v} = K_I \left(\dfrac{k\alpha_1}{k(\alpha_1 - \beta_1) + v} r_{\text{in}} - w \right) & \text{for} \quad v \geq k\beta_1. \end{cases} \tag{S41}$$

These equations give rise to the block diagram depicted in Fig. 6(c) by defining the error $e \triangleq r_{\text{in}} - w$.

S5 Functional Realizations via Molecular Sequestration

Consider the two sequestration networks depicted in Fig. 7(a) and (b). The general dynamics for both can be written as

$$\begin{cases} \dot{v}_1 = u_0 - \eta v_1 v_2 - \delta_1 v_1 \\ \dot{v}_2 = u - \eta v_1 v_2 - \delta_2 v_2 \\ y = h(v_2), \end{cases} \tag{S42}$$

where u and y denote the input and output of the networks, respectively. The two networks differ by their output functions h where we have $h(v_2) = kv_2$ for Fig. 7(a); whereas $h(v_2) = k/(1 + v_2/\kappa)$ for Fig. 7(b). Note that h can take any other functional form. Applying Theorem 1 with $W_1(x, v_1, v_2) \triangleq u_0 - \delta_1 v_1$ and $W_2(x, v_1, v_2) \triangleq u - \delta_2 v_2$ yields the following reduced dynamics

$$\begin{aligned} \dot{v} &= u_0 - u + \delta_2 \max(-v, 0) - \delta_1 \max(v, 0) \\ y &= h\left(\max(-v, 0)\right), \end{aligned} \tag{S43}$$

where $v \triangleq v_1 - v_2$. Hence at steady-state, when it exists, we have

$$\bar{v} = \begin{cases} \dfrac{u_0 - \bar{u}}{\delta_2} & \text{if} \quad \bar{u} \geq u_0 \\ \dfrac{u_0 - \bar{u}}{\delta_1} & \text{if} \quad u < u_0. \end{cases} \tag{S44}$$

Therefore, invoking Theorem 2 yields the input/output steady-state map of these networks given by

$$\bar{y} = h\left(\frac{1}{\delta_2} \max(\bar{u} - u_0, 0) \right). \tag{S45}$$

Finally, substituting for the specific form of h yields the exact functional forms shown in Fig. 7. Note that if the inducible promoter driving $\mathbf{V_2}$ is leaky with rate u_ℓ, then the effective threshold will be $u_0 - u_\ell$ instead of u_0.

S6 Robustness of the Switching Threshold to Resource-Induced Coupling & Saturation

Consider a slight modification of the sequestration network, depicted in Fig. 7(a), that incorporates resource sharing in the production reactions. As such, the dynamics can be modeled using the framework developed in [33], which incorporates concepts from [34, 35, 79–81]. Specifically, the overall dynamics can now be written as

$$\begin{cases} \dot{v}_1 = u_0 c - \eta v_1 v_2 - \delta_1 v_1 \\ \dot{v}_2 = uc - \eta v_1 v_2 - \delta_2 v_2 \\ \ y = k v_2 c, \end{cases} \tag{S46}$$

where u and y denote the input and output of the networks, respectively, and c is a term that captures the coupling and saturation effects of the shared resources. Specifically, we have $c \triangleq \frac{R^{\mathrm{tot}}}{1+\sum_i s_i}$, where R^{tot} represents the total concentration of the shared resources and s_i denotes all the species connected to this network by the shared resources. Applying Theorem 1 with $W_1(x, v_1, v_2) \triangleq u_0 c - \delta_1 v_1$ and $W_2(x, v_1, v_2) \triangleq uc - \delta_2 v_2$ yields the following reduced dynamics

$$\begin{aligned} \dot{v} &= c(u_0 - u) + \delta_2 \max(-v, 0) - \delta_1 \max(v, 0) \\ y &= k \max(-v, 0) c, \end{aligned} \tag{S47}$$

where $v \triangleq v_1 - v_2$. Hence at steady-state, when it exists, we have

$$\bar{v} = \begin{cases} c\dfrac{u_0 - \bar{u}}{\delta_2} & \text{if} \quad \bar{u} \geq u_0 \\ c\dfrac{u_0 - \bar{u}}{\delta_1} & \text{if} \quad u < u_0. \end{cases} \tag{S48}$$

Therefore, invoking Theorem 2 yields the input/output steady-state map of these networks given by

$$\bar{y} = \frac{kc^2}{\delta_2} \max(\bar{u} - u_0, 0). \tag{S49}$$

Note that $\bar{y} = 0$ as long as $\bar{u} \leq u_0$, maintaining the switching threshold at u_0 despite the shared resources used in the production of $\mathbf{V_1}$, $\mathbf{V_2}$, and possibly other species. This beneficial attribute stems from the symmetry of the sequestration motif. However, it is crucial that the genetic components chosen to implement this motif respect this symmetry, ensuring that $\mathbf{V_1}$ and $\mathbf{V_2}$ utilize identical resources. This principle provides an essential design guideline: the sequestration pairs should be homogeneous, such as protein-protein or mRNA-mRNA interactions. This guideline is naturally respected when using split inteins to implement the anti-windup circuit.

When $\bar{u} > u_0$, the steady-state relationship between u and y may not be exactly linear, depending on how c is influenced by the various species within the closed-loop network. However, linearity is not essential here. The primary requirement is that when the input $\bar{u}$ surpasses the threshold u_0, the output $\bar{y}$ must activate at a sufficiently high level to prevent windup. If the limitations imposed by the resource allocation factor c prevent $\bar{y}$ from reaching this necessary level, the anti-windup circuitry may not function effectively. This limitation stems from the overall burden of shared resources within the cell. Despite this, the dynamic range of the AIF controller is significantly enhanced with the anti-windup circuitry compared to without it.

Next, we demonstrate that the anti-windup circuitry can mitigate windup-induced behaviors, even under burden imposed by limited shared resources. We conduct a numerical simulation study involving shared resources among all production reactions. In this example, we use the CRN realization of Topology I from Fig. 8(a), slightly modifying it to incorporate the effects of limited shared resources. The modified controller circuit is shown in Fig. 8, where $\mathbf{C}$ represents the shared resources

among the production reactions. The differential equations can thus be written as:

$$\begin{aligned}
\dot{x} &= f_\Delta(x, c) + h_a(z_1)ce_1 \\
\dot{z}_1 &= [\mu + h_1(v_2)]\, c - \eta z_1 z_2 \\
\dot{z}_2 &= [h_s(x_L) + h_2(w_2)]\, c - \eta z_1 z_2 \\
\dot{v}_1 &= v_0 c - \eta_v v_1 v_2 - \delta_{v_1} v_1 \\
\dot{v}_2 &= g_1(z_2)c - \eta_v v_1 v_2 - \delta_{v_2} v_2 \\
\dot{w}_1 &= w_0 c - \eta_w w_1 w_2 - \delta_{w_1} w_1 \\
\dot{w}_2 &= g_2(z_1)c - \eta_w w_1 w_2 - \delta_{w_2} w_2 \\
c &= \frac{c^{\text{tot}}}{1 + \frac{x_L}{\kappa_{x_L}} + \frac{z_1}{\kappa_{z_1}} + \frac{z_2}{\kappa_{z_2}} + \frac{v_2}{\kappa_{v_2}} + \frac{w_2}{\kappa_{w_2}}},
\end{aligned} \tag{S50}$$

where c^{tot} is a constant proportional to the total available resources.

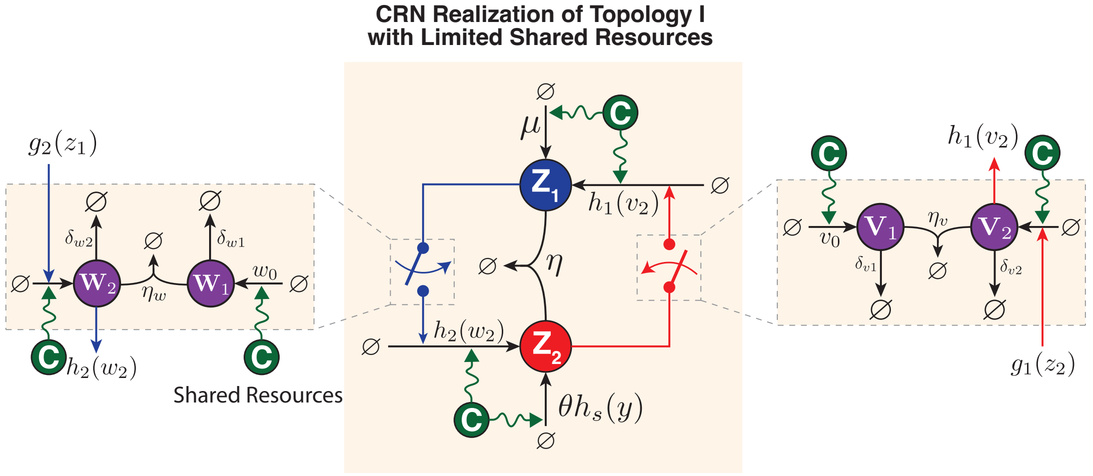

Figure S2: **Incorporating the effect of limited shared resources in the CRN realization of Topology I from Fig. 8(a)**. The shared resources, denoted by **C**, participate in all production reactions.

We consider the simple process from Fig. 2(b). To ensure a fair comparison, we keep all process and controller parameters identical to those in Fig. 10(a). The new parameters related to resource sharing are set as $c^{\text{tot}} = 1$ and $\kappa_{x_L} = \kappa_{z_1} = \kappa_{z_2} = \kappa_{v_2} = \kappa_{w_2} = \kappa$, where κ is kept as a free parameter that can be adjusted to reflect the degree of resource-induced burden. When $\kappa = \infty$, the burden-free scenario from Fig. 10(a) is restored, as repeated in Fig. S3(a) for convenience. In this scenario, $c(t)$ remains one at all times, indicating no burden.

Next, we impose burden by setting $\kappa = 200$. The simulation results, with and without anti-windup circuitry, are shown in Fig. S3(b). Compared to the burden-free case, windup effects are more pronounced, especially after the last disturbance where z_1 grows excessively and fails to return to normal levels within the time window, even after the disturbance ceases. The decrease in c levels indicates the imposed burden. In contrast, with the anti-windup circuitry, the dynamics remain well-behaved, and z_1 is prevented from excessive growth, imposing less burden as indicated by the smaller drops in c levels.

To exaggerate the burden effect, we set $\kappa = 50$ and present the results in Fig. S3(c). In this case, the absence of anti-windup circuitry leads to completely unstable dynamics after the last disturbance, with z_1 growing uncontrollably and c levels continuously dropping. In contrast, adding the anti-windup circuitry mitigates this effect and keeps z_1 levels bounded. Although this severe burden prevents the controller from restoring the setpoint after the disturbance, it still preserves stability. This extreme case illustrates that while high burden prevents the controller from maintaining the setpoint—which is anyway unattainable with a standalone integral controller—it still safeguards the dynamics from instability.

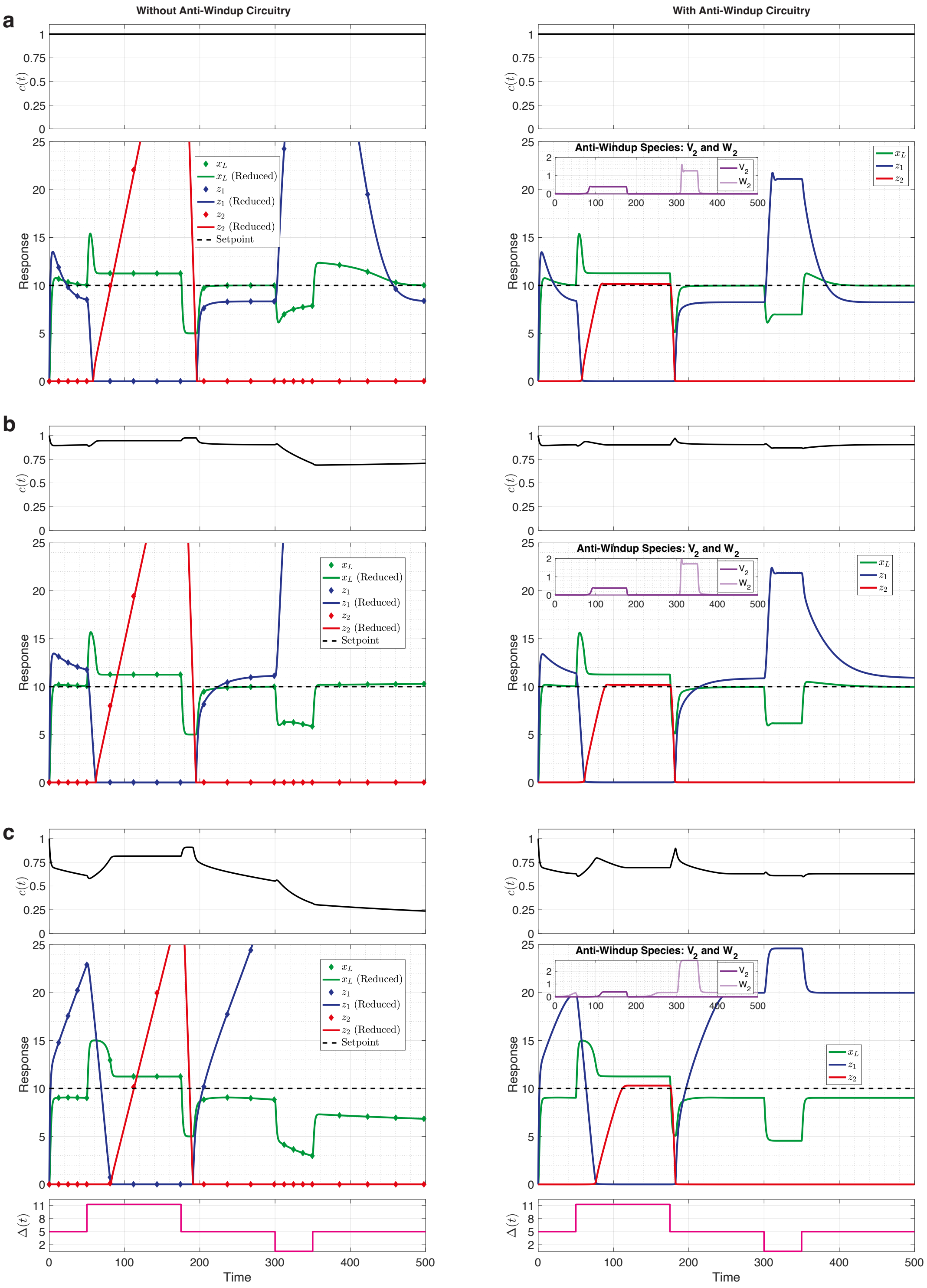

Figure S3: **Simulation results under burden imposed by resource sharing**. All panels depict responses under the same scenario as in the right plot of Fig. 10(a), with a disturbance profile (shown at the bottom) applied while incorporating the effect of shared resources. The dynamics are described by (S50), and the parameters are kept the same as in Fig. 10(a) for fair comparison. The degree of burden is represented by κ. In panel (a), we set $\kappa = 10^5$ to replicate the burden-free case of Fig. 10(a). In panel (b), we impose a moderate burden with $\kappa = 200$. In panel (c), we impose severe burden with $\kappa = 50$.

S7 Supplementary Figures

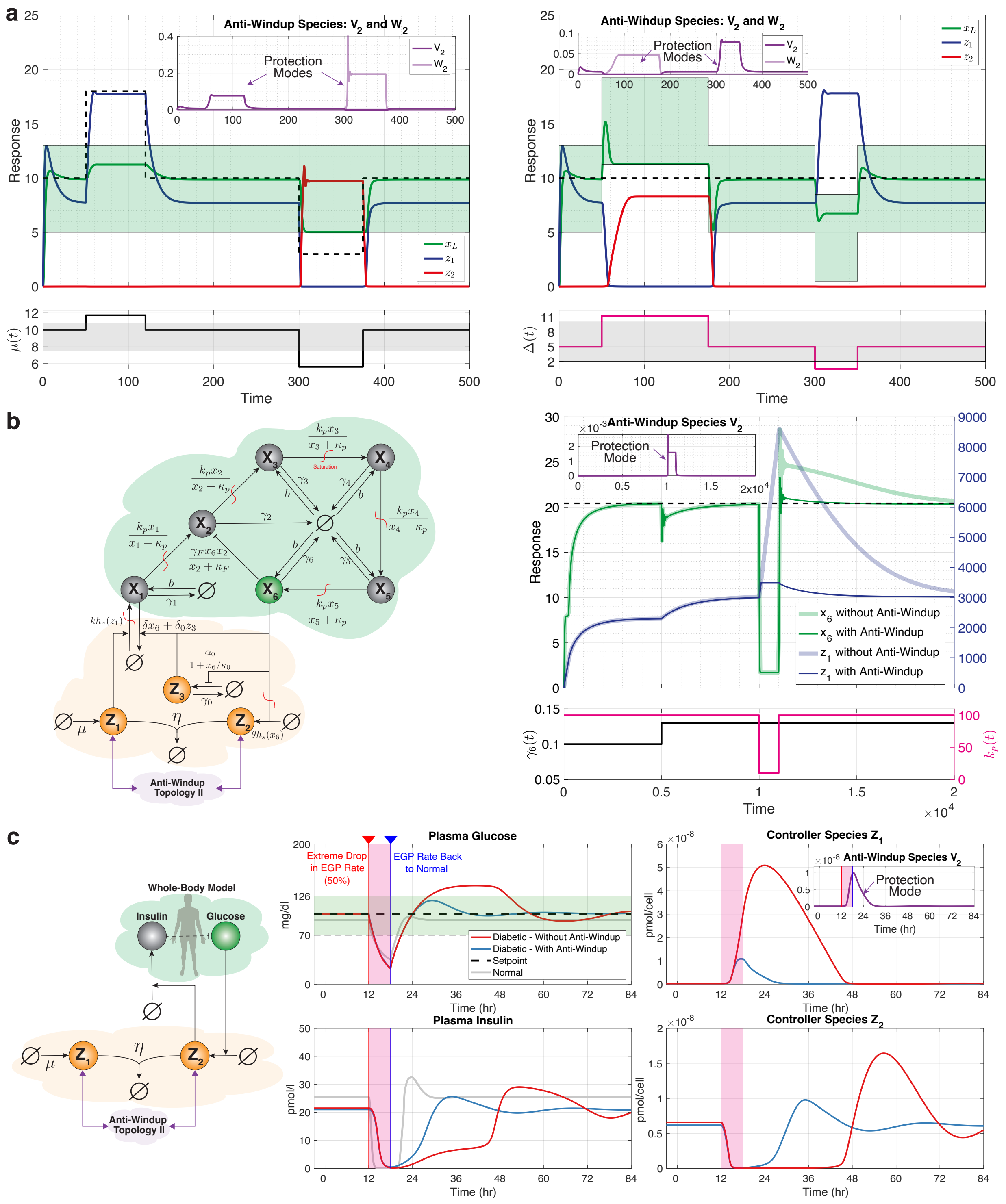

Figure S4: **Reproducing Fig. 10 using anti-windup topology II**. All parameters are the same as those in Fig. 10 except $v_0 = 20, w_0 = 10$ in panel (a), and the functions from Fig. 8 are given by $h_i(v_j) = bv_j$ with $b = 100\mu\text{mol}^{-1}\text{hr}^{-1}$.

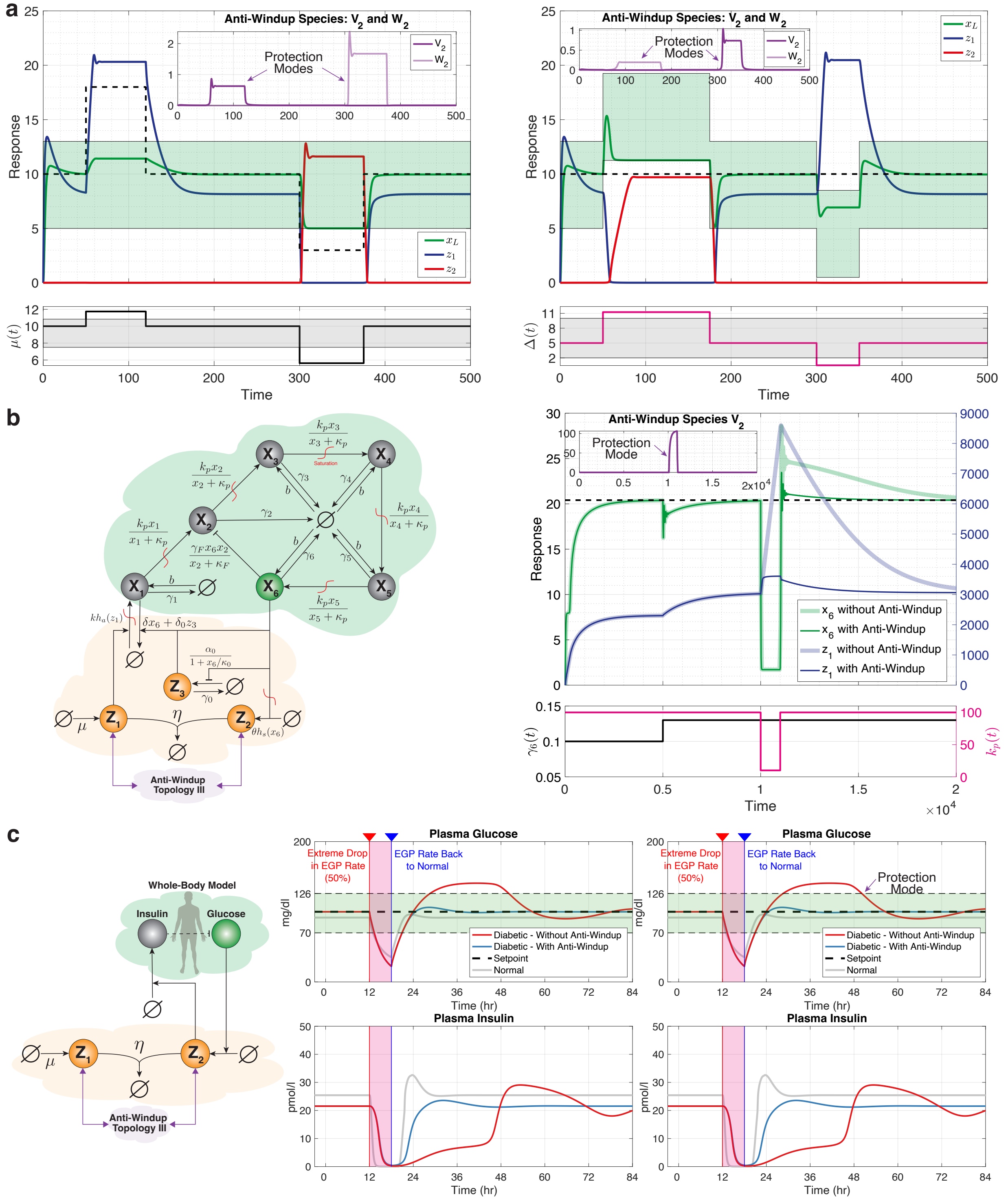

Figure S5: **Reproducing Fig. 10 using anti-windup topology III**. All parameters are the same as those in Fig. 10 except $v_0 = 20, w_0 = 10$ in panel (a). However, the functions from Fig. 8 are now given by $h_i(v_j) = \frac{\alpha}{1+v_j/\kappa}$ with $\alpha = 1, \kappa = 5$ in panel (a), $\alpha = 1, \kappa = 10$ in panel (b) and $\alpha = 1, \kappa = 0.01\mu\text{mol}$ in panel (c).

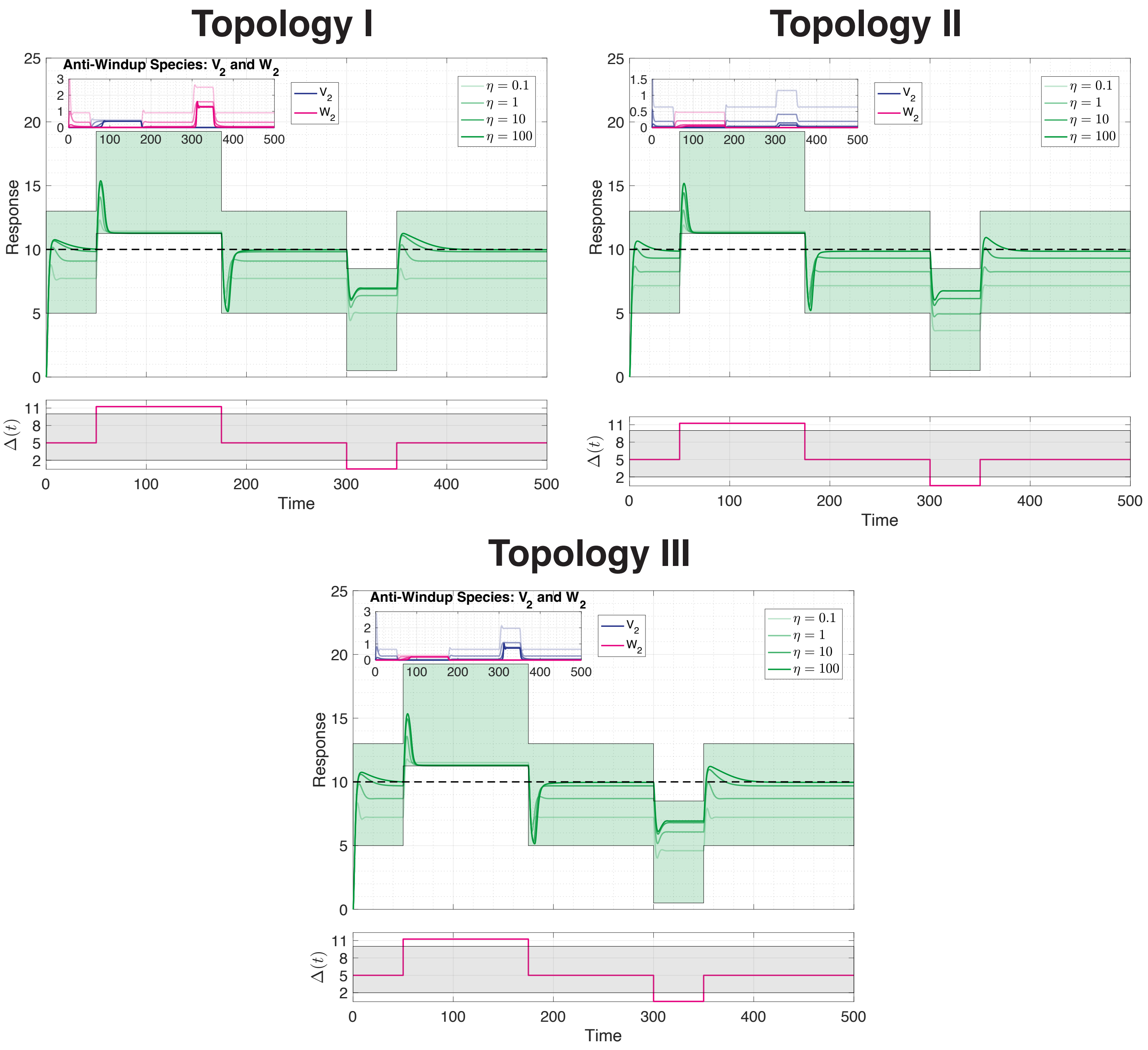

Figure S6: **The effect of the sequestration rates of the three anti-windup topologies**. All parameters are the same as those in Fig. 10(a) for Topology I, Fig. S4(a) for Topology II and Fig.S5(a) for Topology III. The only difference is that $\eta_v = \eta_w$ take the values $\{10^{-1}, 10^0, 10^1, 10^2\}$. For small sequestration rates η_v and η_w, the anti-windup species $\mathbf{V_2}$ and $\mathbf{W_2}$ will have non-zero values even in non-protection modes and thus may interfere with the integrator. As a result, steady-state errors may emerge even when windup is absent. This can be mitigated by having strong sequestration reactions. Observe that for all three topologies, the steady-state error in the non-protection mode decreases to become negligible as η_v and η_w are increased.

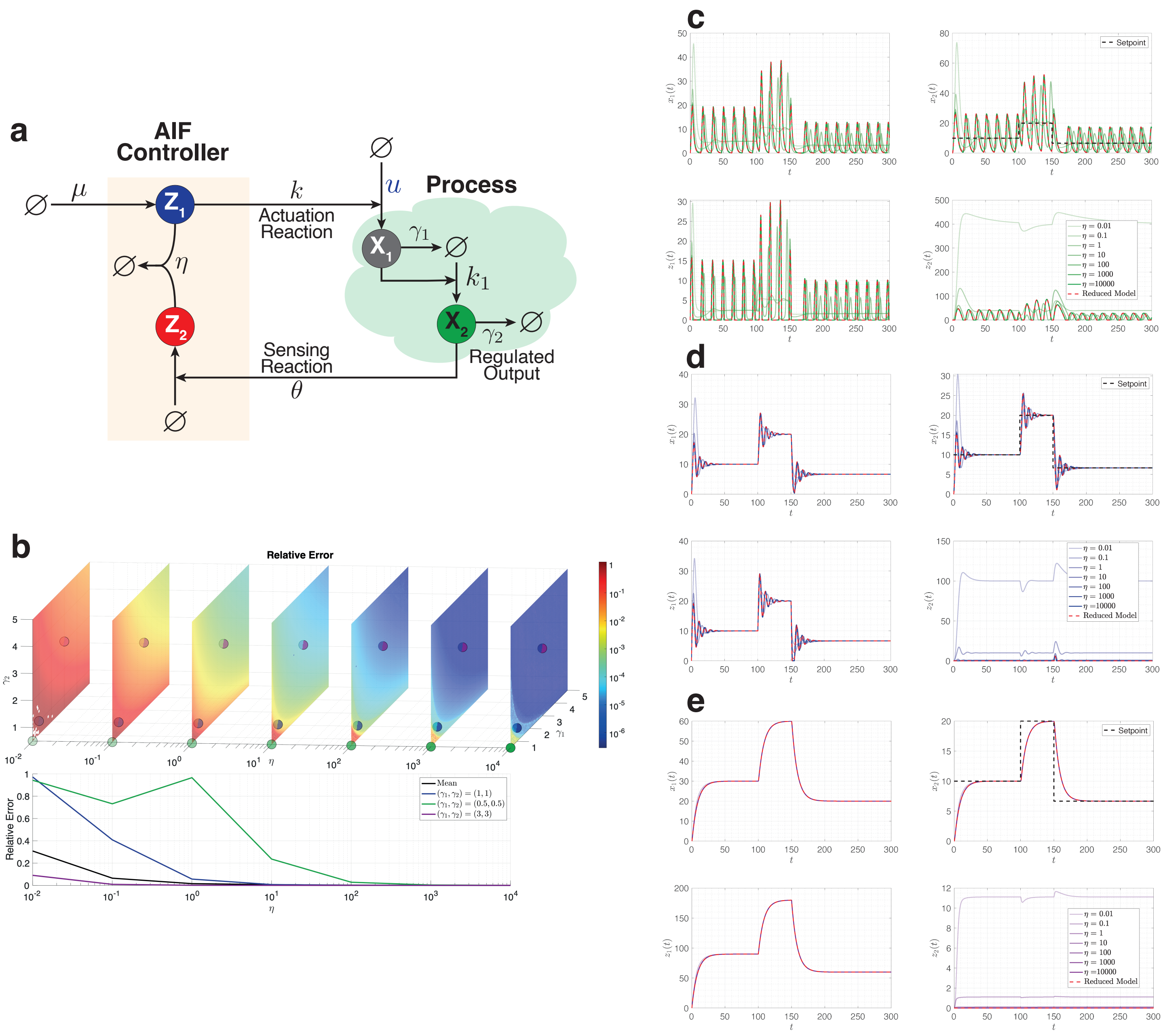

Figure S7: **Deterministic Model Reduction: A Numerical Validation.** (a) The simulations in this figure considers a closed-loop network that integrates the process highlighted in Example 1 with the antithetic integral controller. We set parameters as follows: $\mu = 10, k = \theta = k_1 = 1$. We adjust the values of γ_1 and γ_2 between 0.5 and 5, and increase η logarithmically, ranging from 0.01 to 10^4. (b) An examination of the relative error between the full and reduced model, as supported by Theorem 1. These errors, illustrated as intensity plots, span values of γ_1 and γ_2 for every η. Specifically, we calculate the L^2-norm over time and states (x_1, x_2, z_1, z_2) of the difference in response between the reduced and full model. This is subsequently normalized by the L^2-norm of the full model's response. The graph at the bottom presents the average relative error across (γ_1, γ_2) values and highlights three specific pairs of (γ_1, γ_2). Panels (c), (d), and (e) provide detailed response trajectories of the full model for these three pairs of (γ_1, γ_2). As we elevate η, these trajectories approach the response of the reduced model, which is depicted in red.

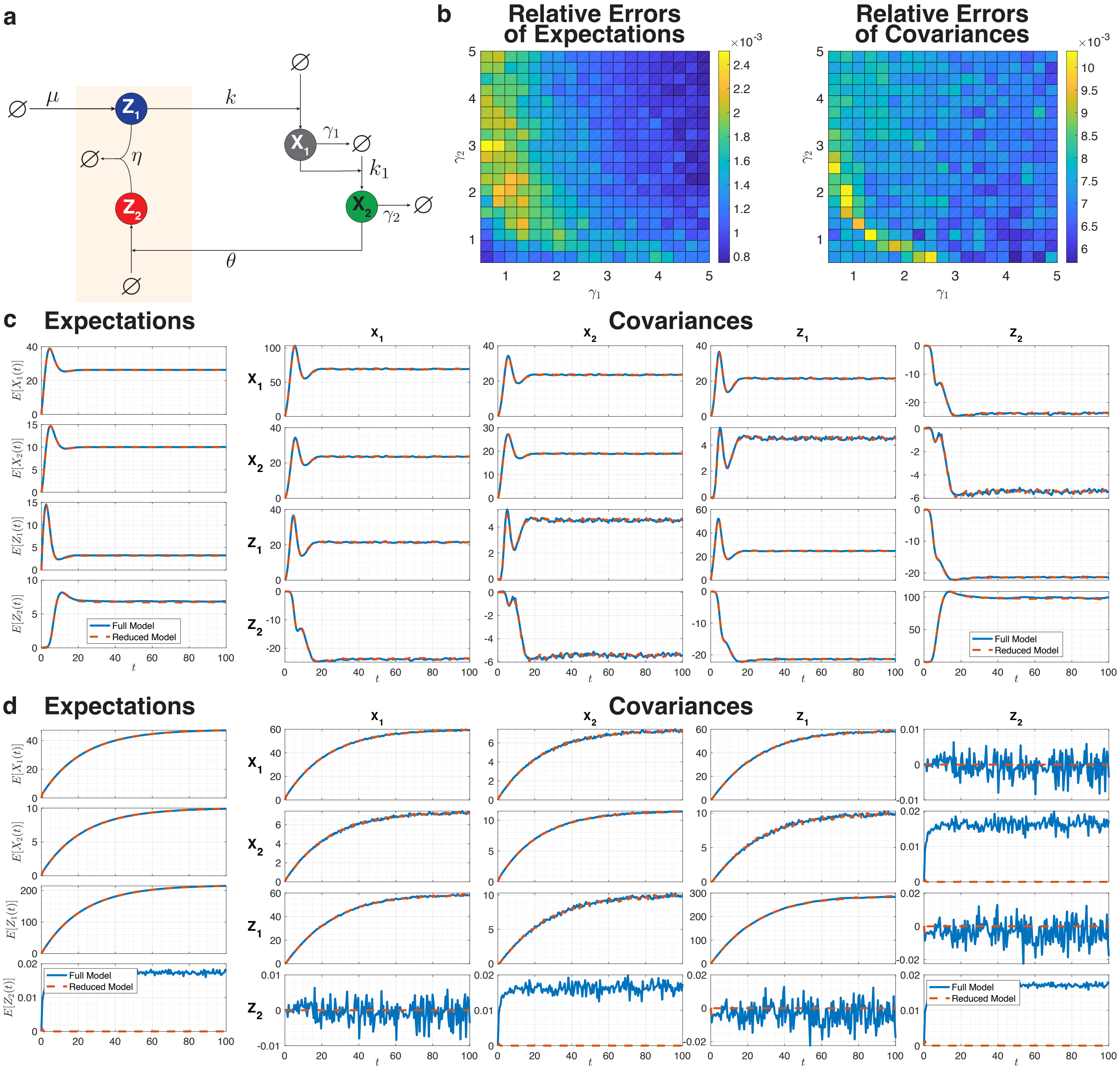

Figure S8: **Stochastic Model Reduction: A Numerical Validation.** (a) The stochastic simulations in this figure consider a closed-loop network that integrates the process highlighted in Example 1 with the antithetic integral controller. We set parameters as follows: $\mu = k_0 = 10, k = \theta = k_1 = 1, \eta = 10^4$. We adjust the values of γ_1 and γ_2 between 0.5 and 5. (b) An examination of the relative error between the full and reduced model at the level of the expectations (left) and covariances (right), as supported by Proposition S3. These errors, illustrated as intensity plots, span values of γ_1 and γ_2. Specifically, we calculate the L^2-norm over time and states (x_1, x_2, z_1, z_2) of the difference in means and covariances between the reduced and full model. This is subsequently normalized by the L^2-norm of the full model's mean and covariance. Panels (c) and (d) provide detailed response trajectories of the expectations and covariances of the full and reduced models for two pairs of $(\gamma_1, \gamma_2) = (0.5, 2.5)$ and $(\gamma_1, \gamma_2) = (4.5, 4.5)$. The simulations demonstrate that the reduced model captures the first two moments of the full model. The small remaining errors are due to a finite $\eta = 10^4$ and due to noise induced by a limited number of trajectories $(N = 10^5)$ generated by Gillespie's stochastic simulation algorithm to compute the dynamics of the moments.

REFERENCES AND NOTES

1. M. Filo, C.-H. Chang, M. Khammash, Biomolecular feedback controllers: From theory to applications. *Curr. Opin. Biotechnol.* **79**, 102882 (2023).

2. D. Del Vecchio, A. J. Dy, Y. Qian, Control theory meets synthetic biology. *J. R. Soc. Interface* **13**, 20160380 (2016).

3. I. Ruolo, S. Napolitano, D. Salzano, M. di Bernardo, D. di Bernardo, Control engineering meets synthetic biology: Foundations and applications. *Curr. Opin. Syst. Biol.* **28**, 100397 (2021).

4. V. Hsiao, A. Swaminathan, R. M. Murray, Control theory for synthetic biology: Recent advances in system characterization, control design, and controller implementation for synthetic biology. *IEEE Control Syst. Mag.* **38**, 32–62 (2018).

5. M. H. Khammash, Perfect adaptation in biology. *Cell Syst.* **12**, 509–521 (2021).

6. C. Briat, A. Gupta, M. Khammash, Antithetic integral feedback ensures robust perfect adaptation in noisy biomolecular networks. *Cell Syst.* **2**, 15–26 (2016).

7. S. K. Aoki, G. Lillacci, A. Gupta, A. Baumschlager, D. Schweingruber, M. Khammash, A universal biomolecular integral feedback controller for robust perfect adaptation. *Nature* **570**, 533–537 (2019).

8. A. Gupta, M. Khammash, Universal structural requirements for maximal robust perfect adaptation in biomolecular networks. *Proc. Natl. Acad. Sci. U.S.A.* **119**, e2207802119 (2022).

9. S. Anastassov, M. Filo, C.-H. Chang, A cybergenetic framework for engineering intein-mediated integral feedback control systems. M. Khammash, *Nat. Commun.* **14**, 1337 (2023).

10. T. Frei, C.-H. Chang, M. Filo, A. Arampatzis, M. Khammash, A genetic mammalian proportional–integral feedback control circuit for robust and precise gene regulation. *Proc. Natl. Acad. Sci. U.S.A.* **119**, e2122132119 (2022).

11. H.-H. Huang, Y. Qian, D. Del Vecchio, A quasi-integral controller for adaptation of genetic modules to variable ribosome demand. *Nat. Commun.* **9**, 5415 (2018).

12. A. Mallozzi, V. Fusco, F. Ragazzini, D. di Bernardo, A general strategy to engineer high-performance mammalian whole-cell biosensors. bioRxiv 582526 [Preprint] (2024). https://doi.org/10.1101/2024.02.28.582526.

13. D. K. Agrawal, R. Marshall, V. Noireaux, E. D. Sontag, In vitro implementation of robust gene regulation in a synthetic biomolecular integral controller. *Nat. Commun.* **10**, 5760 (2019).

14. S. Kumar, M. Rullan, M. Khammash, Rapid prototyping and design of cybergenetic single-cell controllers. *Nat. Commun.* **12**, 5651 (2021).

15. N. Olsman, F. Xiao, J. C. Doyle, Architectural principles for characterizing the performance of antithetic integral feedback networks. *iScience* **14**, 277–291 (2019).

16. N. Olsman, A.-A. Baetica, F. Xiao, Y. P. Leong, R. M. Murray, J. C. Doyle, Hard limits and performance tradeoffs in a class of antithetic integral feedback networks. *Cell Syst.* **9**, 49–63.e16 (2019).

17. C. Briat, A biology-inspired approach to the positive integral control of positive systems: The antithetic, exponential, and logistic integral controllers. *SIAM J. Appl. Dyn. Syst.* **19**, 619–664 (2020).

18. C. Briat, M. Khammash, Optimal and $H\infty$ control of stochastic reaction networks. *arXiv*. 2201.13375. (2022).

19. M. Filo, M. Khammash, Optimal parameter tuning of feedback controllers with application to biomolecular antithetic integral control, in *2019 IEEE 58th Conference on Decision and Control (CDC)* (IEEE, 2019), pp. 951–957.

20. Y. Qian, D. Del Vecchio, Realizing ‘integral control’ in living cells: How to overcome leaky integration due to dilution?. *J. R. Soc. Interface* **15**, 20170902 (2018).

21. C. Briat, A. Gupta, M. Khammash, Antithetic proportional-integral feedback for reduced variance and improved control performance of stochastic reaction networks. *J. R. Soc. Interface* **15**, 20180079 (2018).

22. M. Filo, S. Kumar, M. Khammash, A hierarchy of biomolecular proportional-integral-derivative feedback controllers for robust perfect adaptation and dynamic performance. *Nat. Commun.* **13**, 2119 (2022).

23. M. Chevalier, M. Gómez-Schiavon, A. H. Ng, H. El-Samad, Design and analysis of a proportional-integral-derivative controller with biological molecules. *Cell Syst.* **9**, 338–353.e10 (2019).

24. M. Filo, S. Kumar, S. Anastassov, M. Khammash, Exploiting the nonlinear structure of the antithetic integral controller to enhance dynamic performance, in *2022 IEEE 61st Conference on Decision and Control (CDC)* (IEEE, 2022), pp. 1294–1299.

25. M. Filo, M. Hou, M. Khammash, A hidden proportional feedback mechanism underlies enhanced dynamic performance and noise rejection in sensor-based antithetic integral control. *bioRxiv*. 2023.04.16.537062. (2023).

26. E. J. Hancock, D. A. Oyarzún, Stabilization of antithetic control via molecular buffering. *J. R. Soc. Interface* **19**, 20210762 (2022).

27. C. C. Samaniego, E. Franco, Ultrasensitive molecular controllers for quasi-integral feedback. *Cell Syst.* **12**, 272–288.e3 (2021).

28. C. C. Samaniego, E. Franco, An ultrasensitive biomolecular network for robust feedback control. *IFAC-PapersOnLine* **50**, 10950–10956 (2017).

29. V. Martinelli, D. Salzano, D. Fiore, M. di Bernardo, Multicellular PI control for gene regulation in microbial consortia. *IEEE Contr. Syst. Lett.* doi.org/10.1109/LCSYS.2022.3184922, (2023).

30. V. Martinelli, D. Salzano, D. Fiore, M. di Bernardo, Multicellular PI control for gene regulation in microbial consortia. *IEEE Contr. Syst. Lett.* **6**, 3373–3378 (2022).

31. D. Salzano, B. Shannon, C. Grierson, L. Marucci, N. J. Savery, M. d Bernardo, In-vivo multicellular control of gene expression in microbial consortia. *bioRxiv*. 2023.11.13.566807. (2023).

32. K. J. Åström, R. M. Murray, *Feedback Systems: An Introduction for Scientists and Engineers* (Princeton Univ. Press, 2021).

33. T. Frei, F. Cella, F. Tedeschi, J. Gutiérrez, G.-B. Stan, M. Khammash, V. Siciliano, Characterization and mitigation of gene expression burden in mammalian cells. *Nat. Commun.* **11**, 4641 (2020).

34. Y. Qian, H.-H. Huang, J. I. Jiménez, D. Del, Resource competition shapes the response of genetic circuits, *ACS Synthetic Biol.* **6**, 1263–1272 (2017).

35. Y. Rondelez, Competition for catalytic resources alters biological network dynamics. *Phys. Rev. Lett.* **108**, 018102 (2012).

36. 36. T . Plesa, A. Dack, T. E. Ouldridge, Integral feedback in synthetic biology: Negative-equilibrium catastrophe. *J. Math Chem*. **61**, 1980–2018 (2023).

37. S. Tarbouriech, M. Turner, Anti-windup design: An overview of some recent advances and open problems. *IET Contr. Theory Appl.* **3**, 1–19 (2009).

38. K. J. Astrom, L. Rundqwist, Integrator windup and how to avoid it, in *1989 American Control Conference* (IEEE, 1989), pp. 1693–1698.

39. K. Astrom, A.-B. Ostberg, A teaching laboratory for process control. *IEEE Control Syst. Mag.* **6**, 37–42 (1986).

40. L. Rundqwist, Anti-reset windup for PID controllers. *IFAC Proc. Vol.* **23**, 453–458 (1990).

41. A. Visioli, *Practical PID Control* (Springer Science & Business Media, 2006).

42. L. B. Carey, D. van Dijk, P. M. Sloot, J. A. Kaandorp, E. Segal, Promoter sequence determines the relationship between expression level and noise. *PLOS Biol.* **11**, e1001528 (2013).

43. J. F. Kugel, J. A. Goodrich, Promoter escape limits the rate of RNA polymerase II transcription and is enhanced by TFIIE, TFIIH, and ATP on negatively supercoiled DNA. *Proc. Natl. Acad. Sci. U.S.A.* **95**, 9232–9237 (1998).

44. A. D. Attie, R. T. Raines, Analysis of receptor-ligand interactions. *J. Chem. Educ.* **72**, 119 (1995).

45. B. R. Caré, H. A. Soula, Impact of receptor clustering on ligand binding. *BMC Syst. Biol.* **5**, 48 (2011).

46. B. D. Bennett, E. H. Kimball, M. Gao, R. Osterhout, S. J. Van Dien, J. D. Rabinowitz, Absolute metabolite concentrations and implied enzyme active site occupancy in *Escherichia coli*. *Nat. Chem. Biol.* **5**, 593–599 (2009).

47. C. D. Man, F. Micheletto, D. Lv, M. Breton, B. Kovatchev, C. Cobelli, The UVA/PADOVA type 1 diabetes simulator: New features. *J. Diabetes Sci. Technol.* **8**, 26–34 (2014).

48. C. Dalla Man, D. M. Raimondo, R. A. Rizza, C. Cobelli, Gim, simulation software of meal glucose–insulin model. *J. Diabetes Sci. Technol.* **1**, 323–330 (2007).

49. K. J. Åström, T. Hägglund, K. J. Astrom, *Advanced PID control* , vol. 461 (ISA-The Instrumentation, Systems, and Automation Society Research Triangle Park, 2006).

50. M. V. Kothare, P. J. Campo, M. Morari, C. N. Nett, A unified framework for the study of anti-windup designs. *Automatica* **30**, 1869–1883 (1994).

51. C. Briat, Sign properties of Metzler matrices with applications. *Linear Algebra Appl.* **515**, 53–86 (2017).

52. B. A. Francis, W. M. Wonham, The internal model principle of control theory. *Automatica* **12**, 457–465 (1976).

53. F. Nóbel, H. De Battista, S. Nuñez, J. Picó, Reference conditioning anti-windup for the biomolecular antithetic controller. *IFAC PapersOnLine* **52**, 156–162 (2019).

54. R. Hanus, M. Kinnaert, J.-L. Henrotte, Conditioning technique, a general anti-windup and bumpless transfer method. *Automatica* **23**, 729–739 (1987).

55. N. E. Buchler, F. R. Cross, Protein sequestration generates a flexible ultrasensitive response in a genetic network. *Mol. Syst. Biol.* **5**, 272 (2009).

56. C. Sarmiento, J. A. Camarero, Biotechnological applications of protein splicing. *Curr. Protein Pept. Sci.* **20**, 408–424 (2019).

57. B. Dassa, N. London, B. L. Stoddard, O. Schueler-Furman, S. Pietrokovski, Fractured genes: A novel genomic arrangement involving new split inteins and a new homing endonuclease family. *Nucleic Acids Res.* **37**, 2560–2573 (2009).

58. F. Pinto, E. L. Thornton, B. Wang, An expanded library of orthogonal split inteins enables modular multi-peptide assemblies. *Nat. Commun.* **11**, 1529 (2020).

59. C. W. Lennon, M. Belfort, Inteins. *Curr. Biol.* **27**, R204–R206 (2017).

60. N. H. Shah, T. W. Muir, Inteins: Nature's gift to protein chemists. *Chem. Sci.* **5**, 446–461 (2014),.

61. B. P. Kovatchev, M. Breton, C. Dalla Man, C. Cobelli, In silico preclinical trials: A proof of concept in closed-loop control of type 1 diabetes. *J. Diabetes Sci. Technol.* **3**, 44–55 (2009).

62. C. Cosentino, R. Ambrosino, M. Ariola, M. Bilotta, A. Pironti, F. Amato, On the realization of an embedded subtractor module for the control of chemical reaction networks. *IEEE Trans Automat Contr* **61**, 3638–3643 (2016).

63. K. Oishi, E. Klavins, Biomolecular implementation of linear I/O systems. *IET Syst. Biol.* **5**, 252–260 (2011).

64. C. C. Samaniego, G. Giordano, E. Franco, *2019 18th European Control Conference (ECC)* (IEEE, 2019), pp. 692–697.

65. C. C. Samaniego, J. Kim, E. Franco, Sequestration and delays enable the synthesis of a molecular derivative operator, in *2020 59th IEEE Conference on Decision and Control (CDC)* (IEEE, 2020), pp. 5106–5112.

66. E. Alexis, C. C. Schulte, L. Cardelli, A. Papachristodoulou, Biomolecular mechanisms for signal differentiation. *Iscience* **24**, 103462 (2021).

67. N. M. Paulino, M. Foo, J. Kim, D. G. Bates, PID and state feedback controllers using DNA strand displacement reactions. *IEEE Contr. Syst. Lett.* **3**, 805–810 (2019).

68. M. Whitby, L. Cardelli, M. Kwiatkowska, L. Laurenti, M. Tribastone, M. Tschaikowski, PID control of biochemical reaction networks. *IEEE Trans Automat Contr* **67**, 1023–1030 (2022).

69. E. Alexis, L. Cardelli, A. Papachristodoulou, On the design of a PID bio-controller with set point weighting and filtered derivative action. *IEEE Contr. Syst. Lett.* **6**, 3134–3139 (2022).

70. Y. Zhang, C. C. Samaniego, K. Carleton, Y. Qian, G. Giordano, E. Franco, Building molecular band-pass filters via molecular sequestration, in *2022 IEEE 61st Conference on Decision and Control (CDC)* (IEEE, 2022), pp. 3890–3895.

71. A. Moorman, C. C. Samaniego, C. Maley, R. Weiss, A dynamical biomolecular neural network, in *2019 IEEE 58th Conference on Decision and Control (CDC)* (IEEE, 2019), pp. 1797–1802.

72. C. C. Samaniego, N. A. Delateur, G. Giordano, E. Franco, Biomolecular stabilisation near the unstable equilibrium of a biological system, in *2019 IEEE 58th Conference on Decision and Control (CDC)* (IEEE, 2019), pp. 958–964.

73. C. C. Samaniego, Y. Qian, K. Carleton, E. Franco, Building subtraction operators and controllers via molecular sequestration. *IEEE Contr. Syst. Lett.* **7**, 3361–3366 (2023).

74. T. W. Grunberg, D. Del Vecchio, Time-scale separation based design of biomolecular feedback controllers, in *2019 IEEE 58th Conference on Decision and Control (CDC)* (IEEE, 2019), pp. 6616–6621.

75. A. M. Zand, M. S. Tavazoei, N. V. Kuznetsov, Chaos and its degradation-promoting-based control in an antithetic integral feedback circuit. *IEEE Contr. Syst. Lett.* **6**, 1622–1627 (2022).

76. Y. Qian, D. Del Vecchio, A singular singular perturbation problem arising from a class of biomolecular feedback controllers. *IEEE Contr. Syst. Lett.* **3**, 236–241 (2019).

77. P. Carvajal-Vallejos, R. Pallissé, H. D. Mootz, S. R. Schmidt, Unprecedented rates and efficiencies revealed for new natural split inteins from metagenomic sources. *J. Biol. Chem.* **287**, 28686–28696 (2012).

78. D. T. Gillespie, Stochastic simulation of chemical kinetics. *Annu. Rev. Phys. Chem.* **58**, 35–55 (2007).

79. M. Carbonell-Ballestero, E. Garcia-Ramallo, R. Montañez, C. Rodriguez-Caso, J. Macia, Dealing with the genetic load in bacterial synthetic biology circuits: Convergences with the Ohm's law. *Nucleic acids Res.* **44**, 496–507 (2016).

80. A. Y. Weiße, D. A. Oyarzún, V. Danos, P. S. Swain, Mechanistic links between cellular trade-offs, gene expression, and growth. *Proc. Natl. Acad. Sci. U.S.A.* **112**, E1038 (2015).

81. A. Gyorgy, J. I. Jiménez, J. Yazbek, H.-H. Huang, H. Chung, R. Weiss, D. Del Vecchio, Isocost lines describe the cellular economy of genetic circuits. *Biophys. J.* **109**, 639–649 (2015).

82. H. K. Khalil, Improved performance of universal integral regulators. *J. Optim. Theory Appl.* **115**, 571–586 (2002).

83. G. S. Katzenberger, *Solutions of a Stochastic Differential Equation Forced onto a Manifold by a Large Drift* (The University of Wisconsin-Madison, 1991).

84. H.-W. Kang, T. G. Kurtz, Separation of time-scales and model reduction for stochastic reaction networks. *Ann. Appl. Probab.* **23**, 529–583 (2013).
